# AP‐2 reduces amyloidogenesis by promoting BACE1 trafficking and degradation in neurons

**DOI:** 10.15252/embr.201947954

**Published:** 2020-04-23

**Authors:** Sujoy Bera, Santiago Camblor‐Perujo, Elena Calleja Barca, Albert Negrete‐Hurtado, Julia Racho, Elodie De Bruyckere, Christoph Wittich, Nina Ellrich, Soraia Martins, James Adjaye, Natalia L. Kononenko

**Affiliations:** ^1^ CECAD Research Center University of Cologne Cologne Germany; ^2^ Institute for Stem Cell Research and Regenerative Medicine Medical Faculty Heinrich Heine University Düsseldorf Germany; ^3^ Present address: Centre for Neuroscience and Regenerative Medicine Faculty of Science University of Technology Sydney Sydney NSW Australia

**Keywords:** amyloidogenesis, axonal transport, BACE1, endocytosis, neurodegeneration, Membrane & Intracellular Transport, Molecular Biology of Disease, Neuroscience

## Abstract

Cleavage of amyloid precursor protein (APP) by BACE‐1 (β‐site APP cleaving enzyme 1) is the rate‐limiting step in amyloid‐β (Aβ) production and a neuropathological hallmark of Alzheimer's disease (AD). Despite decades of research, mechanisms of amyloidogenic APP processing remain highly controversial. Here, we show that in neurons, APP processing and Aβ production are controlled by the protein complex‐2 (AP‐2), an endocytic adaptor known to be required for APP endocytosis. Now, we find that AP‐2 prevents amyloidogenesis by additionally functioning downstream of BACE1 endocytosis, regulating BACE1 endosomal trafficking and its delivery to lysosomes. AP‐2 is decreased in iPSC‐derived neurons from patients with late‐onset AD, while conditional AP‐2 knockout (KO) mice exhibit increased Aβ production, resulting from accumulation of BACE1 within late endosomes and autophagosomes. Deletion of BACE1 decreases amyloidogenesis and mitigates synapse loss in neurons lacking AP‐2. Taken together, these data suggest a mechanism for BACE1 intracellular trafficking and degradation via an endocytosis‐independent function of AP‐2 and reveal a novel role for endocytic proteins in AD.

## Graphical abstract

BACE1 promotes A• production and is a neuropathological hallmark of Alzheimer's disease. This study shows that the endocytic adaptor protein complex‐2 promotes trafficking and degradation of BACE1 in neurons and reduces amyloidogenesis.

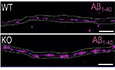

## Introduction

Alzheimer's disease (AD) is the most prominent neurodegenerative disorder and a leading cause of dementia in the aging population 1. It is well established that the increased amyloidogenic processing of amyloid precursor protein (APP), resulting in augmented production of either total amyloid‐β (Aβ) or a shift in the Aβ_1–40_:Aβ_1–42_ ratio toward formation of the more toxic Aβ_1–42_, are key features underlying the pathogenesis of AD 2, 3. Based on the amyloid cascade hypothesis, an imbalance between Aβ production and clearance 1 results in increased amounts of Aβ in the form of monomers, oligomers, insoluble fibrils, and plaques. High levels of Aβ induce tau hyperphosphorylation and formation of neurofibrillary tangles in specific brain regions in AD patients, triggering synaptic dysfunction, inflammation, and oxidative stress in affected cells 4, 5, 6. Thus, identifying cellular processes involved in the generation of Aβ peptide in neurons is of great importance for the development of novel drug targets or predictive biomarkers in AD.

Aβ peptide is liberated from the membrane‐spanning APP by sequential proteolytic cleavage, employing β‐ and γ‐secretases 7. APP is first cleaved by the β‐site APP cleaving enzyme 1 (BACE1) to generate the N‐terminal soluble APPβ (sAPPβ) and the membrane‐bound APP C‐terminal fragment C99, which is subsequently cleaved by the γ‐secretase to produce the Aβ. Conversely, APP can also be cleaved by α‐secretases, which releases the soluble ectodomain portion of APPα (sAPPα) and prevents Aβ formation 8, or by the η‐secretase, the alternative proteolytic processing pathway occurring under physiological conditions 9. The cleavage of APP by BACE1 represents the rate‐limiting step for Aβ generation 10. The absence of Aβ plaques is reported in BACE1 null mice engineered to overexpress human APP (Tg2576+) 11, while double‐transgenic mice obtained by crossing the mice that overexpress BACE1 and APP show enhanced Aβ generation and exacerbated Aβ pathology 12. These data, along with the fact that both the activity and the expression pattern of BACE1 are elevated in human sporadic AD patients 13, 14, provide a conceptual basis for a BACE1‐dependent mechanism of Aβ accumulation and suggest BACE1 as a primary drug target for AD therapy 10. However, BACE1 cleavage of several other substrates besides APP 15 is important for normal physiology 16, 17, putting the safety of BACE1 inhibitors in question. These data taken together with the recent finding that oral BACE1 inhibitor Verubecestat does not slow disease progression in AD patients as compared with placebo 18 raise the demand for novel therapeutic targets in AD. An alternative approach to BACE1 inhibitors is an indirect intervention with BACE1 protein levels in the brain through the regulation of BACE1 delivery to degradation organelles. However, despite two decades passing since the discovery of BACE1 19, 20, the precise regulation of BACE1 intracellular trafficking in neurons remains incompletely understood.

In neurons, BACE1 and APP are segregated during resting conditions, while neuronal activity induces their convergence into acidic microdomains (early and late endosomes) 21, providing an optimal environment for BACE1 enzymatic activity and APP cleavage 22. While APP trafficking from the plasma membrane to endosomes is well established, intracellular sorting of BACE1 remains highly controversial. On the one hand, it has been postulated that BACE1 via the di‐leucine motif in the cytoplasmic tail binds clathrin adaptor protein complex‐2 (AP‐2), which facilitates its endocytosis from the plasma membrane 23, 24. Conversely, the trafficking of BACE1 along a clathrin‐independent pathway, regulated by the small GTPase ADP ribosylation factor 6 (ARF6), has also been proposed 25. How these two pathways mechanistically contribute to the amyloidogenic processing of APP in neurons is currently unknown.

AP‐2 is a heterotetrameric complex comprised of α, β, μ, and σ subunits that links clathrin and other endocytic proteins to sites of clathrin‐mediated endocytosis (CME) 26. Knockout of AP‐2(μ) in mice causes early embryonic lethality 27, and depletion of AP‐2(μ) in mammalian non‐neuronal cells results in potent inhibition of CME due to loss of the entire AP‐2 complex 28. While the β, μ, and σ subunits of AP‐2 complex are made from a single gene in mammals, the α subunit is encoded by two isogenes termed αA and αC that undergo alternative splicing in the brain 29. Interestingly, AP‐2 is not absolutely required for cargo internalization via CME in neurons, but has a prominent role in the reformation of synaptic vesicles from endosome‐like vacuoles 30, 31 and in transport of autophagosomes via the association of the appendage domain of the AP‐2αA with autophagy modifier LC3 32. Loss of neuronal AP‐2 in mice impairs BDNF/TrkB signaling pathway and causes neurodegeneration 32. Here, we demonstrate that AP‐2 affects APP processing and Aβ production in neurons via the regulation of BACE1 intracellular trafficking. While not essential for BACE1 endocytosis, AP‐2 controls BACE1 protein levels by mediating its trafficking *en route* to lysosomes. Strikingly, AP‐2 is decreased in human iPSC‐derived neurons from patients with late‐onset AD. Taken together, our data identify a previously undescribed function of AP‐2 in regulation of BACE1 levels in the brain and suggest a novel role for endocytic adaptors in AD.

## Results

### Endosomal trafficking, but not BACE1 endocytosis, requires AP‐2

**Figure EV1:**
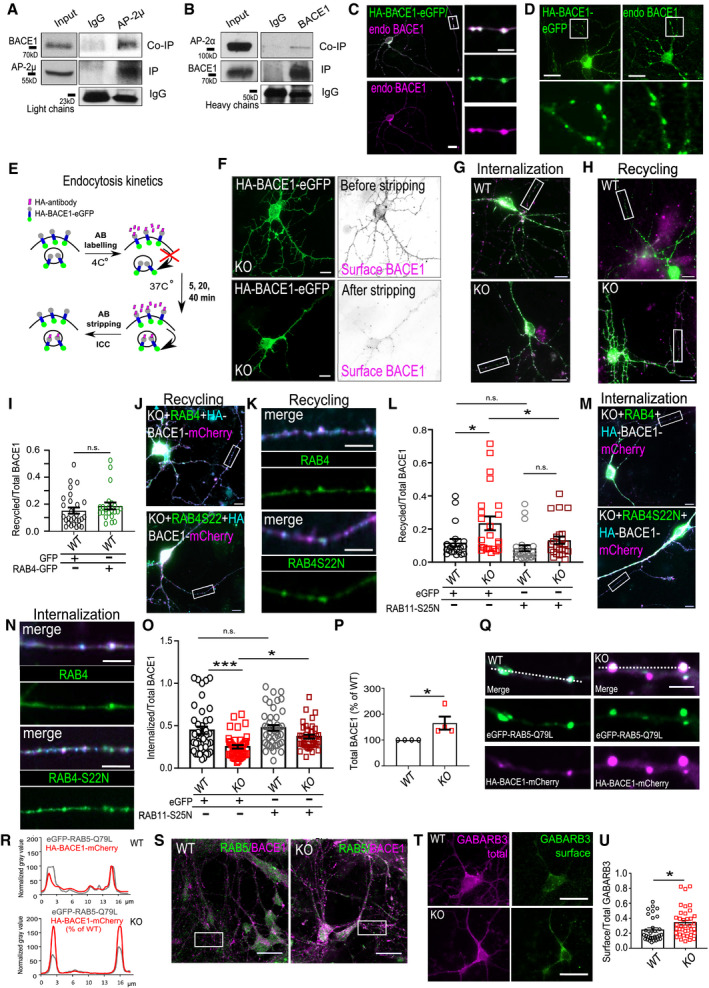
AP‐2 regulates the recycling of BACE1 in the brain ABACE1 is co‐immunoprecipitated (Co‐IP) by the AP‐2μ‐specific antibody (IP) from the mouse brain cortex (high exposure was used to show the input).BAP‐2α is co‐immunoprecipitated (Co‐IP) by the BACE1‐specific antibody (IP) from the mouse brain cortex (high exposure was used to show the input).CRepresentative fluorescence images of neurons expressing the HA‐BACE1‐eGFP and immunostained for endogenous BACE1. Scale bars: 20 μm (left panel), 5 μm (zoomed images).DRepresentative fluorescence images of neurons expressing the HA‐BACE1‐eGFP or non‐transfected neurons immunostained for endogenous BACE1. Scale bars: 20 μm.ESchematic illustration of the assay used in Fig 1A.FAcid‐stripping control for the removal of the membrane‐bound HA antibody in AP‐2μ KO neurons, overexpressing the HA‐BACE1‐eGFP. Scale bar: 10 μm.GOverview images of HA‐BACE1‐eGFP‐expressing neurons shown in Fig 1C. Scale bar: 15 μm.HOverview images of HA‐BACE1‐eGFP‐expressing neurons shown in Fig 1E. Scale bar: 15 μm.IRecycled‐to‐total BACE1 ratio in WT neurons overexpressing either eGFP or eGFP‐RAB4. Twenty‐eight eGFP‐expressing neurons and 22 eGFP‐RAB4‐expressing neurons, *N* = 2 biological replicates.J, KOverview images of HA‐BACE1‐mCherry‐expressing neurons, additionally co‐expressing either GFP‐RAB4 or GFP‐RAB4S22N, shown in Fig 1G. Magnified axonal fragments are shown in (K). Scale bars: 10 μm.LRecycled levels of BACE1 after 20 min of HA antibody chase in HA‐BACE1‐mCherry‐transfected WT and KO neurons, co‐expressing either eGFP or eGFP‐RAB11‐S25N, calculated as the HA(recycled)/mCherry(total) signal intensity ratio (WT^GFP^: 0.12 ± 0.02, KO^GFP^: 0.23 ± 0.04, WT^GFP‐RAB11‐S25N^: 0.08 ± 0.02, KO^GFP‐RAB11‐S25N^: 0.13 ± 0.02, pWT^GFP^ versus pKO^GFP^ = 0.024, pWT^GFP^ versus pWT^GFP‐RAB11‐S25N^ = 0.840, pKO^GFP^ versus pKO^GFP‐RAB11‐S25N^ = 0.047, pWT^GFP‐RAB11‐S25N^ versus pKO^GFP‐RAB11‐S25N^ = 0.587, 20–24 neurons per condition, *N* = 3 biological replicates).M, NOverview images of HA‐BACE1‐mCherry‐expressing neurons, additionally co‐expressing either GFP‐RAB4 or GFP‐RAB4S22N, shown in Fig 1J. Magnified axonal fragments are shown in (N). Scale bars: 10 μm.OLevels of internalized BACE1 are increased in AP‐2μ KO neurons overexpressing the RAB11‐S25N mutant (WT^GFP^: 0.46 ± 0.05, KO^GFP^: 0.26 ± 0.02, WT^GFP‐RAB11A‐S25N^: 0.49 ± 0.03, KO^GFP‐RAB11A‐S25N^: 0.38 ± 0.03, pWT^GFP^ versus pKO^GFP^ = 0.000, pWT^GFP^ versus pWT^GFP‐RAB11‐S25N^ = 0.919; pKO^GFP^ versus pKO^GFP‐RAB11‐S25N^ = 0.044, pWT^GFP‐RAB11‐S25N^ versus pKO^GFP‐RAB11‐S25N^ = 0.105, 40‐42 neurons for each condition, *N* = 5 biological replicates).PLevels of BACE1 are significantly increased in AP‐2μ KO‐cultured neuronal lysates compared to the WT set to 100% (KO: 165.42 ± 25.3, *P* = 0.040, also see Fig 1L). *N* = 4 biological replicates.Q, RLoss of AP‐2μ does not impair the trafficking of BACE1 toward early endosomes, marked by overexpression of RAB5‐Q79L in WT and AP‐2μ KO neurons. Line plot analysis in (R) indicates BACE1 distribution in early endosomes marked by dotted lines in (Q). Scale bar: 5 μm.SOverview images of neurons shown in Fig 1O. Scale bar, 50 μm.T, UOverview images of WT and AP‐2μ KO neurons immunostained for surface and total GABARB3. Ratio of surface to total GABARB3 is increased in KO neurons compared to WT (WT: 0.24 ± 0.03, KO: 0.34 ± 0.03, *P* = 0.024, 32 WT and 38 KO neurons, *N* = 3 biological replicates). Scale bar: 20 μm.Data information: Rectangles in Appendix Fig S1G, H, J and M indicate regions magnified in Fig 1. All graphs show mean ± SEM; statistical analysis was performed by unpaired two‐tailed Student's *t*‐test in (I, U), two‐way ANOVA in (L, O), and one‐sample Student's *t*‐test in (P). n.s.—non‐significant. * indicates *P* ≤ 0.05; ** indicates *P* ≤ 0.01; *** indicates *P* ≤ 0.001.

**Figure 1:**
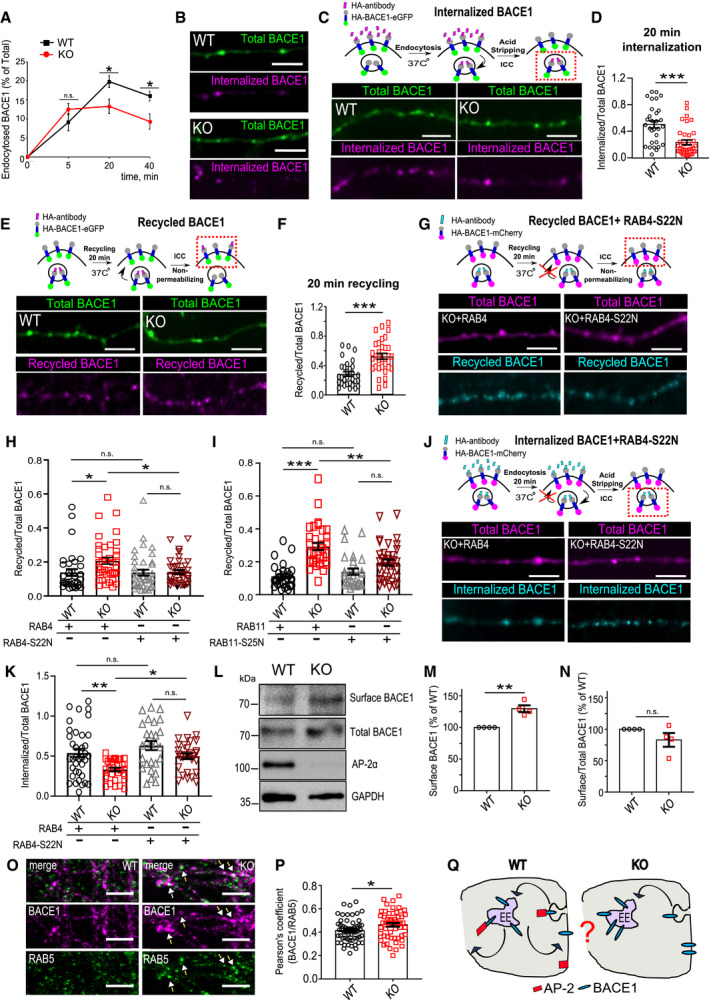
AP‐2 regulates BACE1 recycling and is mostly dispensable for BACE1 endocytosis ABACE1 internalization kinetics after 20 min (WT: 19.79 ± 1.89%, KO: 13.28 ± 1.57%, p^WT^ ^versus^ ^KO^ = 0.017) and 40 min of HA antibody chase (WT: 15.99 ± 2.04, KO: 9.29 ± 1.33, p^WT^ ^versus^ ^KO^ = 0.010) is significantly decreased in AP‐2μ KO neurons (27 WT and 29 KO neurons).BRepresentative examples of internalized BACE1 after 5 min of HA antibody pulse in HA‐BACE1‐eGFP‐overexpressing WT and KO neurons. Scale bar: 5 μm.C, DInternalized BACE1 levels after 20 min of HA antibody pulse in HA‐BACE1‐eGFP‐overexpressing WT and KO neurons, calculated as the HA (internalized)/eGFP (total) signal intensity ratio (WT: 0.50 ± 0.052, KO: 0.23 ± 0.036, *P* = 0.000, 30 WT and 36 KO neurons, *N* = 4 biological replicates). Scale bar: 5 μm.E, FRecycled BACE1 levels after 20 min of HA antibody pulse in HA‐BACE1‐eGFP‐overexpressing WT and KO neurons, calculated as the HA(recycled)/eGFP(total) signal intensity ratio (WT: 0.29 ± 0.031, KO: 0.53 ± 0.040, *P* < 000, 30 WT and 32 KO neurons, *N* = 4 biological replicates). Scale bar: 5 μm.G, HRecycled levels of BACE1 after 20 min of HA antibody pulse in HA‐BACE1‐mCherry‐transfected WT and KO neurons, co‐expressing either eGFP‐RAB4 or eGFP‐RAB4‐S22N, calculated as the HA(recycled)/mCherry(total) signal intensity ratio (WT^RAB4^: 0.14 ± 0.02, KO^RAB4^: 0.20 ± 0.02, WT^RAB4‐S22N^: 0.14 ± 0.02, KO^RAB4S22N^:0.14 ± 0.01, pWT^RAB4^ versus pKO^RAB4^ = 0.033, pKO^RAB4^ versus pKO^RAB4S22N^ = 0.035, pWT^RABS22N^ versus pKO^RABS22N^ = 0.999, pWT^RAB4^ versus pWT^RAB4S22N^ > 0.999, 34–38 neurons, *N* = 4 biological replicates). Scale bar: 5 μm.IRecycled levels of BACE1 after 20 min of HA antibody chase in HA‐BACE1‐mCherry‐transfected WT and KO neurons, co‐expressing either eGFP‐RAB11 or eGFP‐RAB11‐S25N, calculated as the HA(recycled)/mCherry(total) signal intensity ratio (WT^RAB11^: 0.11 ± 0.01, KO^RAB11^: 0.29 ± 0.02, WT^RAB11A‐S25N^: 0.14 ± 0.02, KO^RAB11A‐S25N^: 0.19 ± 0.02, pWT^RAB11^ versus pKO^RAB11^<0.0001, pWT^RAB11^ versus pWT^RAB11A‐S25N^ = 0.761, pKO^RAB11^ versus pKO^RAB11A‐S25N^ = 0.001, pWT^RAB11A‐S25N^ versus pKO^RAB11A‐S25N^ = 0.175, 28–37 neurons per condition, *N* = 3 biological replicates).J, KInternalized levels of BACE1 after 20 min of HA antibody uptake in HA‐BACE1‐mCherry‐transfected WT and KO neurons, co‐expressing either eGFP‐RAB4 or eGFP‐RAB4‐S22N, calculated as the HA(internalized)/mCherry(total) signal intensity ratio (WT^RAB4^: 0.54 ± 0.05, KO^RAB4^: 0.33 ± 0.02, WT^RAB4‐S22N^: 0.63 ± 0.06, KO^RAB4‐S22N^: 0.50 ± 0.04, pWT^RAB4^ versus pKO^RAB4^ = 0.004, pKO^RAB4^ versus pKO^RAB4S22N^ = 0.046, pWT^RAB4S22N^ versus pKO^RAB4S22N^ = 0.182, pWT^RAB4^ versus pWT^RAB4S22N^ = 0.450, 27–37 neurons per condition, *N* = 3 biological replicates). Scale bar: 5 μm.L, MBACE1 surface levels are increased in AP‐2μ KO neurons, when compared to the WT set to 100% (KO: 129.65 ± 5.30%, *P* = 0.005, *N* = 4 biological replicates).NSurface versus total BACE1 ratio is not altered in AP‐2μ KO neurons when normalized to the WT set to 100% (KO: 83.02 ± 10.93%, *P* = 0.109, *N* = 4 biological replicates).O, PAccumulation of endogenous BACE1 in RAB5‐positive early endosomes, quantified using Pearson's co‐localization coefficient (WT: 0.41 ± 0.01, KO: 0.46 ± 0.02, p^WT^ versus p^KO^ = 0.021, 58 WT and 54 KO neurons, *N* = 4 biological replicates). Scale bar: 10 μm. Arrows indicate BACE1 accumulation within RAB5‐positive endosomes.QSchematic illustration depicting the role of AP‐2 in BACE1 endosomal trafficking.Data information: Experimental designs are depicted in the top panels in (C, E, G, J), where analyzed BACE1 fraction is marked with the red square. All graphs show mean ± SEM; statistical analysis was performed by unpaired two‐tailed Student's *t*‐test in (A, D, F, P), two‐way ANOVA in (H, I, K), and one‐sample Student's *t*‐test in (M, N). n.s.—non‐significant. * indicates *P* ≤ 0.05; ** indicates *P* ≤ 0.01; *** indicates *P* ≤ 0.001.

Previous results identified that a substantial pool of BACE1 is delivered to endosomes by the AP‐2‐dependent internalization from the plasma membrane 23. Taken into account the fact that AP‐2 and BACE1 are found in a complex in the mouse brain (Fig EV1A and B), we asked whether AP‐2 regulates BACE1 endocytosis in neurons. To test this, we measured the kinetics of BACE1 endocytosis in primary neurons isolated from the cortex of AP‐2 knockout (KO) mice, where the loss of the entire AP‐2 heterotetramer without a compensatory increase in AP‐1 and AP‐3 protein levels is achieved by a tamoxifen‐inducible CAG‐Cre‐dependent recombination of floxed AP‐2μ allele (*Ap‐2μ* ^lox/lox^: *CAG‐*Cre) 32. To investigate the BACE1 trafficking, we took advantage of the previously characterized BACE1‐eGFP construct additionally carrying a single HA tag after the propeptide cleavage site at the N‐terminus 23. WT and KO neurons, transiently expressing the HA‐BACE1‐eGFP that has a localization similar to endogenous BACE1 (Fig EV1C and D), were pulsed with the HA antibody for 30 min at 4°C to label the fraction of BACE1 on the plasma membrane, and endocytosed BACE1 was detected after 5, 20, or 40 min of chase at 37°C using standard immunocytochemistry procedures combined with acid‐stripping of non‐internalized HA antibody (Figs 1A and EV1E and F). In agreement with previous results 23, we observed that the amount of endocytosed BACE1 was decreased in AP‐2μ KO neurons at 20 and 40 min after the HA antibody pulse (Fig 1A). Surprisingly, AP‐2μ was not required for BACE1 endocytosis 5 min after the beginning of the assay (Fig 1A and B), suggesting that BACE1 endosomal recycling, but not endocytosis, might be AP‐2‐dependent. To test this, we next investigated BACE1 postendocytic trafficking in HA‐BACE1‐eGFP‐expressing WT and AP‐2μ KO neurons by adopting a previously described protocol 33. The amount of internalized BACE1 in neurons pulsed with the HA antibody for 20 min was analyzed after acid‐stripping of non‐endocytosed HA antibody under permeabilizing conditions (schematic in Fig 1C). To analyze BACE1 recycling back to the plasma membrane, the neurons that had undergone the 20‐min pulse with the HA antibody were first acid‐stripped, and internalized BACE1 was further chased for 20 min at 37°C. Subsequently, recycled BACE1 was detected at the plasma membrane under non‐permeabilizing conditions (schematic in Fig 1E). The decrease in BACE1 internalization (Figs 1C and D, and EV1G) was accompanied by a substantial increase in BACE1 recycling back to the plasma membrane (Figs 1E and F, and EV1H), indicating that in the absence of AP‐2, BACE1 is mistrafficked between endosomes and the plasma membrane such that the protein is more rapidly recycled.

To directly test whether blocking endosomal recycling in neurons lacking AP‐2 restores BACE1 trafficking, we overexpressed in WT and AP‐2μ KO neurons a GTP binding‐deficient mutant of RAB4‐S22N, which is known to interfere with fast recycling of cargo proteins from the RAB4‐positive recycling endosomes to the plasma membrane 34. In agreement with the hypothesis above, we found that the overexpression of RAB4‐S22N significantly reduced the recycling of BACE1 in neurons lacking AP‐2μ (Figs 1G and H, and EV1I–K). We also prevented the slow RAB11‐dependent endosomal recycling of BACE1 by overexpression in control and AP‐2μ KO neurons of a dominant‐negative S25N form of RAB11A 35. The amount of BACE1 found under recycling conditions at the surface of AP‐2μ KO neurons overexpressing mutant eGFP‐RAB11A‐S25N was significantly reduced compared to either RAB11‐eGFP (Fig 1I)‐ or eGFP‐expressing KO cells (Fig EV1L), albeit with a slightly lower efficiency when compared to RAB4. Blocking either RAB4‐ or RAB11‐dependent endosomal recycling was sufficient to upregulate the internalized fraction of BACE1 in AP‐2μ KO neurons (Figs 1J and K, and EV1M–O). Taken together, these data strongly indicate that AP‐2 is not required for BACE1 endocytosis and regulates BACE1 endosomal trafficking. Without AP‐2, BACE1 is mistrafficked to a recycling compartment, a phenotype that results in more BACE1 being re‐routed to the plasma membrane in AP‐2 KO neurons. Since faster protein recycling should result in its accumulation on the plasma membrane surface, we performed biotinylation experiments to analyze the surface fraction of endogenous BACE1 in primary neurons lacking AP‐2μ. WT‐ and KO‐cultured neurons were incubated with non‐permeable biotin moieties at 4°C to tag protein domains at the extracellular level. After probing the extracted biotinylated proteins with the BACE1 antibody, we found that although a small fraction of BACE1 was localized at the plasma membrane of AP‐2μ KO neurons, this was not significant when normalized to total BACE1 protein levels (Figs 1L–N and EV1P), suggesting that faster recycling of BACE1 is accompanied by its intracellular retention in the KO condition. These data, together with the fact that more BACE1 was detected in AP‐2μ KO RAB5‐positive early endosomes (Figs 1O and P, and EV1Q–S), indicate that AP‐2μ regulates BACE1 endosomal trafficking via a previously undescribed mechanism (Fig 1Q). Interestingly, although the data described above reveal that AP‐2 is not absolutely required for BACE1 endocytosis, we found that it was indispensable for internalization of GABA_A_ receptor β3 subunit (GABARB3) (Fig EV1T and U), another known cargo of AP‐2 36, suggesting a cargo‐specific role for AP‐2 in endocytosis in neurons.

### BACE1 delivery to lysosomes is defective in AP‐2 KO neurons

**Figure EV2:**
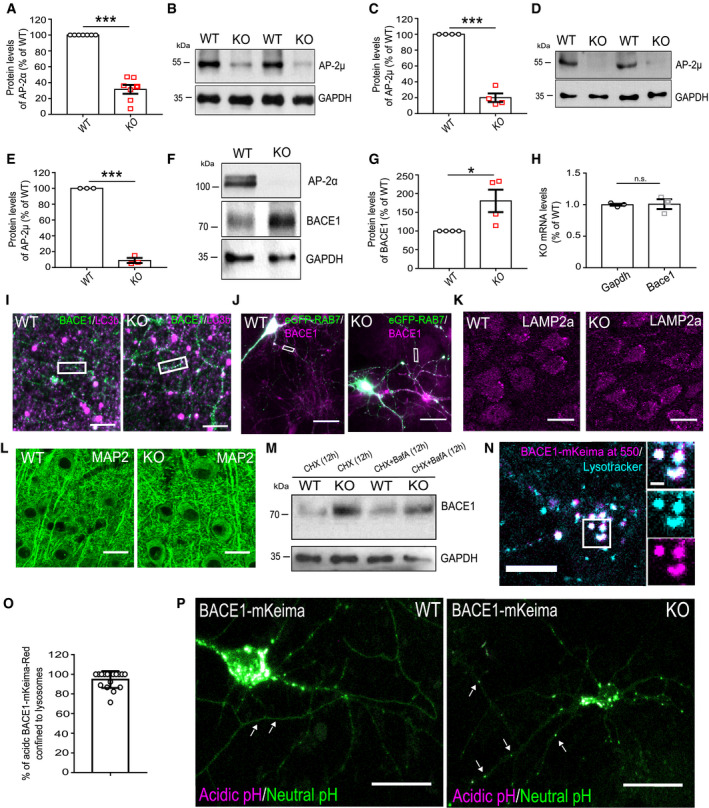
AP‐2μ regulates lysosomal BACE1 levels ASignificantly reduced AP‐2α levels in the cortex of AP‐2μ KO mice. Protein levels in the KO were normalized to the WT set to 100% (KO: 31.49 ± 5.54, *P* < 0.000, *N* = 7 biological replicates).B, CSignificantly reduced AP‐2μ levels in the cortex of AP‐2μ KO mice. Protein levels in the KO were normalized to the WT set to 100% (KO: 20.05 ± 5.39%, *P* = 0.000, *N* = 4 biological replicates).D, ESignificantly reduced levels of AP‐2μ in AP‐2μ KO‐cultured neurons. Protein levels in the KO were normalized to the WT set to 100% (KO: 8.87 ± 3.17%, *P* = 0.000, *N* = 3 biological replicates).F, GSignificantly increased BACE1 levels in AP‐2μ KO‐cultured neurons. Protein levels in the KO were normalized to the WT set to 100% (KO: 180.51 ± 25.99%, *P* = 0.037, *N* = 4 biological replicates).H
*Bace1* mRNA levels measured by qPCR are not significantly altered in AP‐2μ KO neurons (KO/WT^*Bace1*^: 1.00 ± 0.03, KO/WT^*Gapdh*^: 0.99 ± 0.02, *P* = 0.921, *N* = 3 biological replicates). mRNA levels in the KO were normalized to the WT set to 1.IOverview images of neurites represented in Fig 2G. Scale bar, 20 μm.JOverview images of neurites represented in Fig 2I. Scale bar, 20 μm.K, LHorizontal sections from WT and AP‐2μ KO entorhinal cortex immunostained with either LAMP2a (K) or MAP2 (L) antibodies. Scale bars: 20 μmMBACE1 levels are unaltered in lysates from cultured WT and AP‐2μ KO neurons, either treated with 5 μM cycloheximide alone or co‐treated with 80 nM bafilomycin A for 12 h.NRepresentative confocal images of BACE1‐mKeima‐Red‐expressing neurons incubated with LysoTracker Blue‐White for 15 min to label the lysosomes. Scale bar: 10 μm, 1 μm for zoomed images.OPercentage of BACE1‐mKeima‐Red puncta localized to lysosomes (94.61 ± 4.12), 17 cells from *N* = 2 biological replicates.PRepresentative confocal images of BACE1‐mKeima‐Red‐expressing WT and AP‐2μ KO neurons. In the KO, the majority of BACE1‐mKeima organelles at neutral pH was confined to axons located distally from the soma (arrows). Scale bars: 20 μm.Data information: All graphs show mean ± SEM; statistical analysis was performed by unpaired one‐sample Student's *t*‐test in (A, C, E, G, H). n.s.—non‐significant. * indicates *P* ≤ 0.05; ** indicates *P* ≤ 0.01; *** indicates *P* ≤ 0.001.

**Figure 2:**
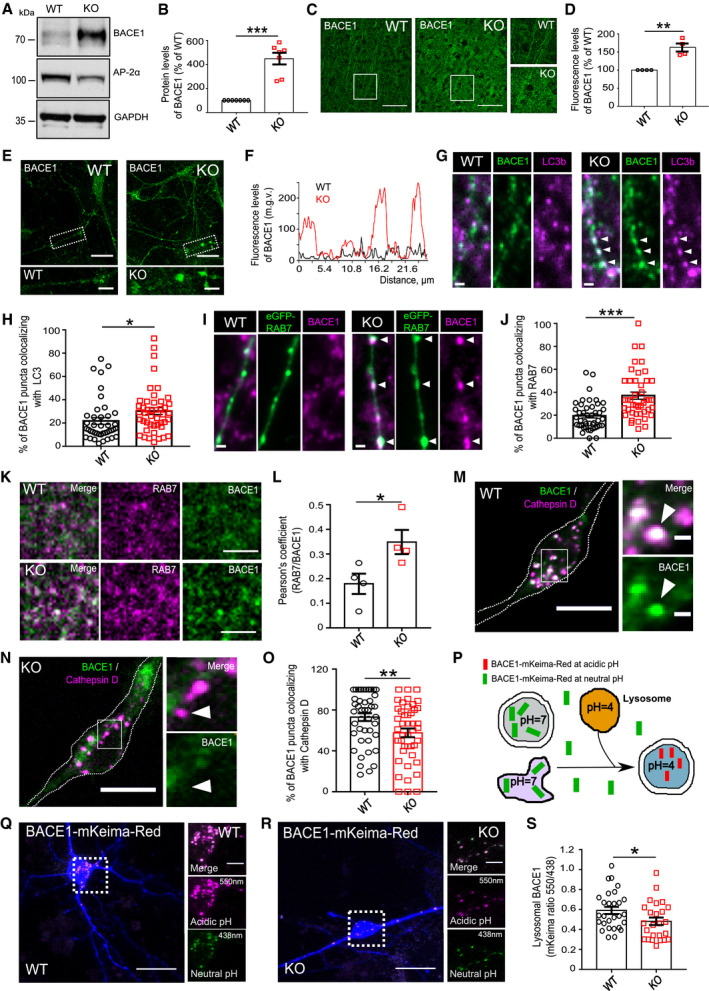
AP‐2 is required for lysosomal targeting of BACE1 A, BBACE1 protein levels in cortical lysates of WT and AP‐2μ KO mice. Protein levels in the KO were normalized to the WT set to 100% (KO: 447.64 ± 48.78%, *P* < 0.000, *N* = 7 biological replicates).C, DImmunofluorescence levels of BACE1 are significantly increased in the cortex of AP‐2μ KO mice when normalized to the WT set to 100% (KO: 162.714 ± 11.082%, *P* = 0.005, *N* = 4, biological replicates). Scale bar: 100 μm.ELocalization of BACE1 in cultured WT and AP‐2μ KO neurons. Scale bars: 20 μm, 5 μm (inserts).FLine plot of BACE1 immunofluorescence along the neurites indicated in (E).G, HPercentage of BACE1 puncta colocalizing with the LC3b (WT: 21.75 ± 2.88, KO: 30.28 ± 2.97, *P* = 0.043, WT = 41, KO = 45 neurons, *N* = 4 biological replicates). Scale bar: 2 μm. Arrowheads indicate BACE1 accumulation within LC3b‐positive organelles.I, JPercentage of BACE1 puncta colocalizing with the eGFP‐RAB7 (WT:19.54 ± 1.91, KO: 37.09 ± 3.13%, *P* < 0.000, WT = 44 and KO = 43 neurons, *N* = 4 biological replicates). Scale bar: 2 μm. Arrowheads indicate BACE1 accumulation within RAB7‐positive organelles.K, LCo‐localization of BACE1 and RAB7A in cortical sections of WT and AP‐2μ KO brains, quantified using Pearson's co‐localization coefficient (WT: 0.18 ± 0.04, KO: 0.35 ± 0.05, *P* = 0.038, *N* = 4 biological replicates). Scale bar: 10 μm.M, NRepresentative fluorescence images of WT and AP‐2μ KO neurons transfected with HA‐BACE1‐mCherry and immunostained for Cathepsin D. Channels were false color‐coded to better illustrate the co‐localization. Scale bars: 10 μm. Arrowheads indicate BACE1 accumulation in lysosomes in the WT, and its absence in the KO. Dotted line outlines the neuron.OLysosomal localization of BACE1 is decreased in KO neurons (WT: 73.05 ± 3.80, KO: 57.68 ± 4.28, *P* = 0.008, 45 WT and 44 KO neurons, *N* = 4 biological replicates).PSchematic illustration of BACE1 delivery to lysosomes monitored via BACE1‐mKeima‐Red.Q, RRepresentative fluorescence images of WT and AP‐2μ KO neurons transfected with BACE1‐mKeima‐Red and co‐transfected with eBFP to reveal the neuronal morphology. Scale bars: 10 μm, 2 μm in zoomed images.SThe 550 nm/438 nm signal intensity ratio of BACE1‐mKeima‐Red quantified over the entire cell is significantly reduced in the KO condition compared to the WT (WT: 0.59 ± 0.04, KO: 0.48 ± 0.04, *P* = 0.041, 28 WT and 25 KO neurons, *N* = 3 biological replicates).Data information: All graphs show mean ± SEM; statistical analysis was performed by unpaired two‐tailed Student's *t*‐test in (H, J, L, O, S) and one‐sample Student's *t*‐test in (B, D). n.s.—non‐significant. * indicates *P* ≤ 0.05; ** indicates *P* ≤ 0.01; *** indicates *P* ≤ 0.001.

To further analyze the role of AP‐2 in BACE1 intracellular trafficking, we examined BACE1 protein levels in cell lysates derived from the cortex of neuron‐confined AP‐2μ KO mice (*Ap2m1* ^lox/lox^: *Tubulin 1α* Cre) 32 (Fig EV2A–C). Using this model, we have previously shown that the levels of major endocytic proteins are unaltered in the absence of AP‐2 32. Since mice lacking AP‐2μ in neurons have been previously reported to die after postnatal day (p) 22, all subsequent *in vivo* experiments were performed with mice between p18 and p21 (Kononenko *et al*, 2017). In agreement with our data in cultured neurons (Figs EV1P and EV2D–G), we found that BACE1 protein levels were almost fivefold upregulated in cortical brain lysates lacking AP‐2μ (Fig 2A and B). BACE1 immunofluorescence levels were also significantly higher in the entorhinal cortex from AP‐2μ KO mice (Fig 2C and D). Moreover, our data revealed that while in control neurons, BACE1 was distributed longitudinally along the neurites, deletion of AP‐2μ resulted in large 3–5 μm spheroid‐like accumulations of BACE1 distally from the cell body (Fig 2E and F). These changes in the protein level were not accompanied by upregulation of BACE1 gene expression (Fig EV2H), indicating that AP‐2 regulates BACE1 proteins levels via a post‐translational mechanism. Since BACE1 is known to be degraded via the lysosomal pathway 37, we postulated that the intracellular accumulation of BACE1 in neurons lacking AP‐2μ might result from its inefficient targeting to lysosomes and, thus, impaired BACE1 degradation. To test this hypothesis, we first analyzed the amount of BACE1 stalled within autophagosomes and late endosomes in control and AP‐2μ KO neurons. In agreement with our previous data (Kononenko *et al*, 2017), we observed significantly more autophagosomes in neurons lacking AP‐2μ that contained significantly more BACE1 when compared to control neurons (Figs 2G and H, and EV2I). Furthermore, increased amount of BACE1 was associated with organelles expressing late‐endosomal marker RAB7A (Figs 2I and J, and EV2J). These data were also corroborated by significantly higher co‐localization of BACE1 with RAB7A in the entorhinal cortex of AP‐2μ KO mice when compared to their control littermates, while levels of lysosomal marker LAMP2a and neuronal marker MAP2 were not altered (Figs 2K and L, and EV2K and L). Since an increase in BACE1 protein levels in AP‐2μ KO neurons persisted upon inhibition of protein synthesis (Fig EV2M) and significantly less BACE1 was localized to AP‐2 KO lysosomes (Fig 2M–O), we directly tested the hypothesis of delayed delivery of BACE1 to lysosomes under the AP‐2μ KO condition. We engineered a construct expressing BACE1 C‐terminally tagged with monomeric Keima‐Red (mKeima). mKeima is a coral‐derived acid‐stable fluorescent protein emitting different colored signals at acidic and neutral pH 38 (Fig 2P), which can be used as a reporter of cargo delivery to lysosomes (Fig EV2N and O). We expressed BACE1‐mKeima‐Red in cultured WT and AP‐2μ KO neurons and analyzed the 550 nm (acid pH)/438 nm (neutral pH) signal intensity ratio using live‐cell confocal microscopy. The amount of BACE1 found in lysosomes of AP‐2μ KO neurons was significantly reduced when compared to control condition (Fig 2Q–S). Interestingly, in the absence of AP‐2 the majority of BACE1‐mKeima organelles with neutral pH was confined to distal processes (Fig EV2P). Taken together, our data indicate that AP‐2 regulates BACE1 delivery to lysosomes in neurons.

### AP‐2 regulates intracellular transport of BACE1 in axons

**Figure EV3:**
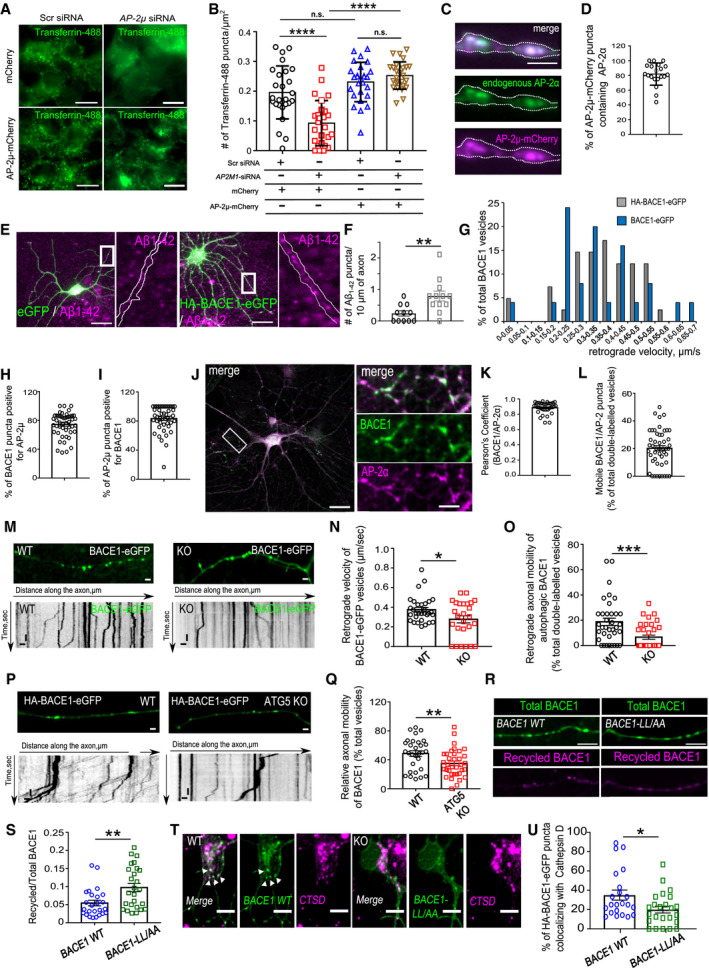
Functionality of AP‐2μ‐mCherry and HA‐BACE1‐eGFP constructs A, BOverexpressed AP‐2μ‐mCherry restores clathrin‐mediated endocytosis of transferrin in HEK cells depleted of endogenous AP‐2μ (mCherry^scr^: 0.19 ± 0.02, mCherry^AP−2μ‐siRNA^: 0.09 ± 0.01, AP‐2μ‐mCherry^scr^: 0.23 ± 0.01, AP‐2μ‐mCherry^AP−2μ‐siRNA^: 0.25 ± 0.01, p^mCherry‐scr^ versus _p_^mCherry‐AP−2μ‐siRNA^ < 0.000; p^mCherry‐scr^ versus p^AP−2μ‐mCherry‐scr^ = 0.289_,_ p^mCherry‐AP−2μ‐siRNA^ versus p^AP−2μ‐mCherry‐AP−2μ–siRNA^ < 0.000, p^AP−2μ‐mCherry‐scr‐siRNA^ versus p^AP−2μ‐mCherry‐AP−2μ–siRNA^  = 0.690, 26 cells for each condition, *N* = 1 biological replicates). Scale bars: 10 μm.C, DLarge portion (81.8 ± 3.37%) of overexpressed AP‐2μ‐mCherry associates with endogenous AP‐2α, 20 axons from 10 neurons. Scale bar: 2 μm.E, FΑβ_1–42_ levels are upregulated in neurons overexpressing the HA‐BACE1‐eGFP, comparing to cells expressing the eGFP only (GFP: 0.24 ± 0.8, BACE1: 0.79 ± 0.16, *P* = 0.006). Eleven eGFP‐ and 12 HA‐BACE1‐eGFP‐expressing neurons, *N* = 1 biological replicates. Scale bars: 20 μm.GHistogram representation of BACE1 retrograde velocity in control neurons overexpressing either the HA‐BACE1‐eGFP or the BACE1‐eGFP (axonal fragments from 28 to 31 neurons, *N* = 3 biological replicates).HPercentage of BACE1 puncta colocalizing with AP‐2μ in control neurons (74.22 ± 2.09%), 48 neurons, *N* = 4 biological replicates.IPercentage of AP‐2μ puncta colocalizing with BACE1 in control neurons (83.35 ± 2.56%), 46 neurons, *N* = 4.J, KCo‐localization of endogenous BACE1 and AP‐2μ, measured using Pearson's co‐localization coefficient (0.88 ± 0.01, 36 neurons, *N* = 3 biological replicates). Scale bars, 20 μm (left panel), 5 μm (zoomed images).LPercentage of mobile BACE1 carriers positive for AP‐2μ in control neurons, normalized to total double‐labeled puncta set to 100% (20.12 ± 1.95%, 48 neurons, *N* = 4 biological replicates).MRepresentative fluorescence images and corresponding kymographs from time‐lapse videos of WT and AP‐2μ KO neurons transfected with BACE1‐eGFP. Scale bars: 2 μm top panels, x = 2 μm, y = 5s bottom panels.NRetrograde velocity of BACE1‐eGFP carriers is significantly reduced in AP‐2μ KO neurons (WT^Retro^: 0.37 ± 0.03, KO^Retro^ = 0.25 ± 0.03, *P* = 0.002, 29 WT and 30 KO neurons, *N* = 3 biological replicates). In these experiments, live‐cell imaging was performed 24 h post‐transfection to exclude the overexpression artifact.ORetrograde mobility of autophagic BACE1 in WT and AP‐2μ KO neurons (WT: 18.74 ± 2.92%, KO: 6.60 ± 1.6%, *P* = 0.000), 36 WT and 36 KO neurons, *N* = 4 biological replicates.P, QSignificantly decreased retrograde mobility of BACE1 in HA‐BACE1‐eGFP‐expressing ATG5 KO neurons comparing to the WT (WT: 48.63 ± 3.94%, KO^ATG5^: 34.96 ± 3.22%, *P* = 0.008, 30 WT and 35 KO neurons, *N* = 3 biological replicates). Scale bars: 2 μm top panels, x = 2 μm, y = 5 s bottom panels.R, SIncreased recycling of BACE1‐LL/AA when compared to WT BACE1 (WT: 0.05 ± 0.00, LL/AA: 0.09 ± 0.01, *P* = 0.003, 25–26 neurons per condition, *N* = 4 biological replicates). Scale bar, 5 μm.TRepresentative images of neurons expressing either HA‐BACE1‐GFP or HA‐BACE1‐LL/AA‐GFP and immunostained for Cathepsin D (CTSD). Scale bar 5 μm.USignificantly decreased percentage of HA‐BACE1‐LL/AA puncta found in lysosomes compared to WT BACE1 (WT: 34.99 ± 5.22%, KO: 19.98 ± 3.49%, *P* = 0.019, 22‐24 neurons per group, *N* = 3 biological replicates.Data information: All graphs show mean ± SEM; statistical analysis was performed by unpaired two‐tailed Student's *t*‐test in (F, N, O, Q, S, U) and two‐way ANOVA in (B). n.s.—non‐significant.* indicates *P* ≤ 0.05; ** indicates *P* ≤ 0.01; *** indicates *P* ≤ 0.001.

**Figure 3:**
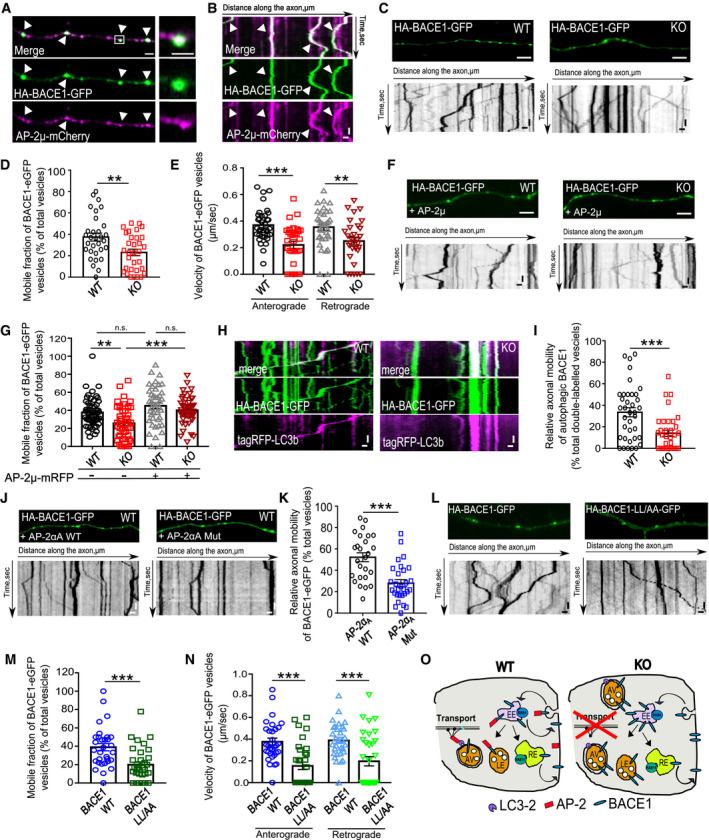
AP‐2 regulates axonal transport of BACE1 A, BRepresentative fluorescence images (A) and corresponding kymographs (B) from time‐lapse videos of control neurons transfected with HA‐BACE1‐eGFP and co‐transfected with AP‐2μ‐mCherry. Scale bars: (A) 2 μm, 1.6 μm inserts, (B) x = 2 μm, y = 5s. Arrowheads in (A) correspond to BACE1 carriers indicated by the same arrowheads in (B).CRepresentative fluorescence images and corresponding kymographs from time‐lapse videos of WT and AP‐2μ KO neurons transfected with HA‐BACE1‐eGFP. Scale bar: 5 μm top panels, x = 2 μm, y = 5s bottom panels.DLoss of AP‐2μ significantly decreases the BACE1 motility (WT: 37.42 ± 3.79%, KO: 23.07 ± 3.07%, *P* = 0.003, WT = 31, KO = 32 neurons, *N* = 4 biological replicates).ELoss of AP‐2μ significantly decreases the anterograde and retrograde BACE1 velocity (WT^Antero^: 0.37 ± 0.02 μm/s, KO^Antero^: 0.22 ± 0.03 μm/s, pWT^Antero versus^ pKO^Antero^ < 0.000; WT^Retro^: 0.35 ± 0.02 μm/s, KO^Retro^: 0.25 ± .03 μm/s, pWT^Retro versus^ pKO^Retro^  = 0.006, WT = 35 and KO = 32 neurons, *N* = 4 biological replicates).FRepresentative fluorescence images and corresponding kymographs from time‐lapse videos of WT and AP‐2μ KO neurons co‐transfected with HA‐BACE1‐GFP and AP‐2μ‐mRFP. Scale bar: 5 μm top panels, x = 2 μm, y = 5s bottom panels.GBi‐directional mobility of BACE1 carriers in WT and AP‐2μ KO neurons, co‐expressing the AP‐2μ‐mRFP (WT: 37.60 ± 2.20%, KO: 25.61 ± 2.67%, WT^AP−2μ‐mRFP^: 45.04 ± 3.27%, KO^AP−2μ‐mRFP^: 40.07 ± 2.37%, p^WT versus KO^ = 0.005, p^KO versus KO+AP−2μ‐mRFP^ < 0.000, p^WT versus WT+AP−2μ‐mRFP^ = 0.186, p^WT+AP−2μ‐mRFP versus KO+AP−2μ‐mRFP^ = 0.570, 43–53 neurons per condition, *N* = 4 biological replicates).HRepresentative kymographs of WT and AP‐2μ KO neurons co‐expressing the HA‐BACE1‐GFP and tagRFP‐LC3B. Scale bars: *x* = 2 μm, *y* = 5 s.IReduced bi‐directional mobility of BACE1‐containing autophagosomes in AP‐2μ KO neurons (WT: 33.53 ± 4.19%, KO: 19.45 ± 5.14%, *P* = 0.000, WT = 37 and KO = 35 neurons, *N* = 4 biological replicates).JRepresentative kymographs of WT neurons co‐expressing HA‐BACE1‐GFP and AP2‐α_A_ WT or AP2–α_A_ Mut. Scale bar: 5 μm top panels, *x* = 2 μm, μ = 5 s bottom panels.KMobility of BACE1 carriers is significantly decreased in neurons overexpressing the AP2‐α_A_ Mut compared to neurons expressing the AP2‐α_A_ WT (WT: 52.23 ± 4.16%, Mut: 27.95 ± 3.31, *P* = < 0.000, WT = 26 and Mut = 29 neurons, *N* = 4 biological replicates).LRepresentative fluorescence images and corresponding kymographs from time‐lapse videos of WT neurons transfected with either HA‐BACE1‐GFP or HA‐BACE1‐LL/AA‐GFP. Scale bar: 5 μm top panels, x = 2 μm, *y* = 5 s bottom panels.MReduced mobility of BACE1‐LL/AA‐positive vesicles, when compared to WT BACE1 (WT: 38.97 ± 1.40%, LL/AA: 19.89 ± 1.77%, *P* < 0.000, WT = 31 and LL/AA = 33 neurons, *N* = 4 biological replicates).NReduced velocity of BACE1‐LL/AA‐positive vesicles, when compared to WT BACE1 (WT^Antero^: 0.38 ± 0.03 μm/s, LL/AA^Antero^: 0.15 ± 0.03 μm/s, pWT^Antero versus^ pLL/AA^Antero^ < 0.000; WT^Retro^: 0.39 ± 0.03 μm/s, LL/AA^Retro^: 0.25 ± .03 μm/s, pWT^Retro versus^ pLL/AA^Retro^  = 0.000, WT = 31 and LL/AA = 33 neurons, *N* = 4 biological replicates).OSchematic illustration of how AP‐2 regulates BACE1 intracellular trafficking and transport in neurons.Data information: All graphs show mean ± SEM; statistical analysis was performed by unpaired two‐tailed Student's *t*‐test in (D, E, I, K, M, N) and two‐way ANOVA in (G). n.s.—non‐significant. * indicates *P* ≤ 0.05; ** indicates *P* ≤ 0.01; *** indicates *P* ≤ 0.001.

What is the mechanism by which AP‐2 controls lysosomal targeting of BACE1? We previously identified a novel non‐canonical function of AP‐2 in retrograde trafficking of TRKB receptors in the axon 32. To test whether AP‐2 also regulates the transport of cargo containing BACE1 vesicles, we first monitored the dynamics of co‐trafficking of AP‐2μ‐mCherry and HA‐BACE1‐eGFP in cultured neurons by live imaging. Tagged AP‐2μ and BACE1 23, 39 were shown functional, since (i) AP‐2μ‐mCherry was able to rescue clathrin‐mediated endocytosis of transferrin in cells depleted for endogenous AP‐2μ (Fig EV3A and B), (ii) AP‐2μ‐mCherry was substantially colocalized with endogenous AP‐2α upon its overexpression in neurons (Fig EV3C and D), (iii) HA‐BACE1‐eGFP increased β‐site cleavage of APP, monitored by the levels of Aβ in HA‐BACE1‐eGFP‐overexpressing cultured neurons (Fig EV3E and F), and iv) kinetics of retrograde BACE1 transport obtained in neurons overexpressing the HA‐BACE1‐eGFP were similar to velocities in neurons overexpressing the BACE1‐eGFP (Fig EV3G). Using these constructs, we observed a close co‐localization and co‐trafficking of AP‐2μ with BACE1 in axons (Figs 3A and B, and EV3H and I). Strong co‐localization was also observed for endogenous proteins, labeled with specific antibodies (Fig EV3J and K). Next, we probed whether AP‐2 is functionally required for BACE1 transport in neurons by analyzing the transport of BACE1‐positive carriers in axons of WT and AP‐2μ KO neurons expressing the HA‐BACE1‐eGFP or BACE1‐eGFP. In WT, axons around 40% of BACE1 puncta displayed bi‐directional movement with an average velocity of about 0.4 μm/s, consistent with previously described kinetics of BACE1 trafficking *in vivo* 40 (Fig 3C). In contrast, both the axonal motility and the velocity of BACE1 vesicles were greatly reduced in AP‐2μ KO axons (Figs 3D and E, and EV3M and N). This phenotype was specific since the re‐expression of AP‐2μ restored the defective transport of BACE1 carriers (Fig 3F and G). Our previously published data indicate that AP‐2 mediates the retrograde transport of axonal cargo via the association of its large brain‐specific isoform AP‐2α_A_ with the autophagy modifier protein LC3B (microtubule‐associated protein 1A/1B light chain 3) and that its absence impairs retrograde transport of LC3‐containing cargo and autophagosome processing in neurons 32. Since a fraction of axonal BACE1 is known to be retrogradely transported in autophagosomes 41, we hypothesized that defective degradation of BACE1 in AP‐2μ KO neurons might result from its impaired axonal transport in autophagosomes. Live‐cell imaging experiments revealed that axonal mobility and specifically the retrograde movement of BACE1‐containing carriers positive for LC3B were greatly reduced in AP‐2μ KO neurons (Figs 3H and I, and EV3O). A similar result was obtained by the overexpression of LC3 binding‐deficient mutant of AP‐2α_A_ in control neurons (Fig 3J and K). In agreement with recently published data 41, autophagosomes were required for efficient transport of BACE1, since significantly reduced axonal mobility of BACE1 was also observed in neurons lacking ATG5, an E3‐like ligase mediating autophagosome expansion and maturation 42 (Fig EV3P and Q). Finally, to directly test whether BACE1 requires AP‐2 for its trafficking in neurons, we overexpressed AP‐2 binding‐deficient mutant of BACE1 (23, LL/AA mutant) in control neurons and measured BACE1 velocity using live‐cell imaging. Interestingly, both the axonal mobility and the velocity of BACE1 vesicles were significantly reduced in neurons overexpressing BACE1 di‐leucine mutant, when compared to neurons expressing the wild‐type BACE1 (Fig 3L–N). Furthermore, this defect was accompanied by increased recycling of endocytosed BACE1 to the plasma membrane, in agreement with 22, 25, and impaired BACE1 delivery to lysosomes, similar to the phenotype described in neuroglioma cells 43 (Fig EV3R–U). Together, these data suggest that AP‐2 regulates BACE1 protein levels in primary neurons via functioning downstream of BACE1 endocytosis, regulating BACE1 endosomal trafficking and its delivery to lysosomes (Fig 3O).

### AP‐2 prevents amyloidogenic processing of APP

**Figure EV4:**
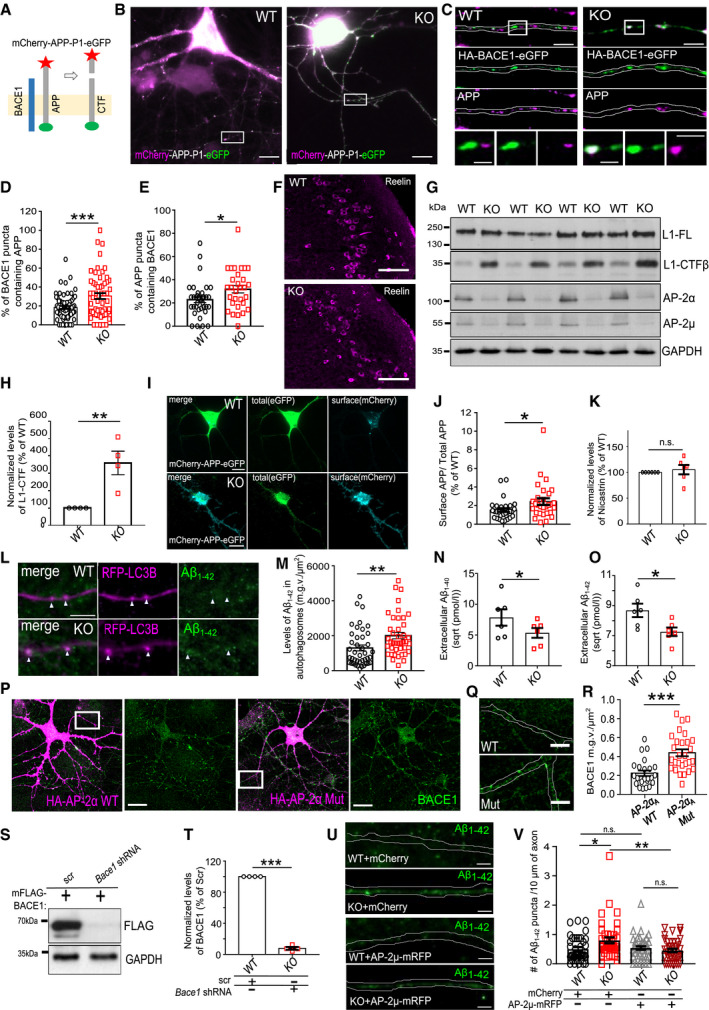
Regulation of APP processing by the AP‐2μ in neurons ASchematic illustration of BACE1 activity monitored using the APP Lys612Val mutant (mCherry‐APP‐P1‐eGFP).BRepresentative fluorescence images of mCherry‐APP‐P1‐eGFP‐expressing WT and AP‐2μ KO neurons, immunostained for mCherry and GFP. Scale bar, 10 μm.CRepresentative fluorescence images of HA‐BACE1‐eGFP‐expressing WT and AP‐2μ KO neurons, immunostained for APP. Scale bars: 5 μm (upper panels), 2 μm (lower panels).DPercentage of BACE1 puncta colocalizing with APP in WT and AP‐2μ KO neurons (WT: 17.27 ± 1.66%, KO: 29.19 ± 3.57%, *P* = 0.003, 40 WT and 40 KO neurons, *N* = 4 biological replicates).EPercentage of APP puncta colocalizing with BACE1 in WT and AP‐2μ KO neurons (WT: 22.78 ± 2.52%, KO: 31.78 ± 3.18%, *P* = 0.030, 36 WT and 30 KO neurons, *N* = 4 biological replicates).FWT and AP‐2μ KO entorhinal cortex of mice at p21 immunostained for Reelin. Scale bars: 50 μm.G, HLevels of L1‐CTFβ are significantly increased in AP‐2μ KO cortex compared to the WT set to 100% (KO^C99^: 359.87 ± 95.77%, *P* = 0.015, *N* = 4 biological replicates).I, JThe mCherry (surface APP)/eGFP (total APP) signal intensity ratio is significantly increased in AP‐2μ KO condition compared to the WT (WT: 1.52 ± 0.19, KO: 2.43 ± 0.36, *P* = 0.028, 30 WT and 29 KO neurons, *N* = 3 biological replicates). Scale bars: 10 μm.KUnaltered levels of Nicastrin in cortical lysates from AP‐2μ KO mice. Protein levels in KO condition were normalized to the WT set to 100% (KO: 105.03 ± 9.02%, *P* = 0.300, *N* = 6 biological replicates).LRepresentative fluorescence images of axons from RFP‐LC3B‐expressing WT and AP‐2μ KO neurons immunostained for Aβ_1–42_. Scale bar: 4 μm. Arrowheads indicate localization of Aβ_1–42_ puncta to LC3B‐positive autophagosomes.MAutophagosomal levels of Aβ_1–42_ are increased in AP‐2μ KO neurons (WT: 1299.58 ± 166.36, KO: 2002.6 ± 185.72, *P* = 0.006, 41 WT and 41 KO neurons, *N* = 3 biological replicates). Scale bar: 5 μm.N, OLevels of secreted Aβ_1–40_ (N) and Aβ_1–42_ (O) are significantly decreased in the media of AP‐2μ KO neurons compared to the WT (WT^Aβ1–40^: 7.85 ± 1.33, KO^Aβ1–40^: 5.35 ± 0.80, *P* = 0.011, *N* = 6 biological replicates; WT^Aβ1–42^: 8.69 ± 0.45, KO^Aβ1–42^: 7.26 ± 0.29, *P* = 0.037, *N* = 6 biological replicates). Values represent pmol/l raw data, which were square‐root‐transformed due to the lack of normality.P–RLevels of BACE1 are increased in control neurons overexpressing the HA‐AP2Mut compared to HA‐AP2α WT‐expressing cells (WT: 0.22 ± 0.03, Mut: 0.44 ± 0.04, *P* < 0.000, 25 WT and 28 Mut neurons, *N* = 3 biological replicates). Rectangles in (P) indicate the area magnified in (Q). Scale bars, 10 μm in (P), 2.5 μm in (Q).S, TAnalysis of BACE1 KD efficiency in HEK cells, expressing the mouse FLAG‐BACE1. *Bace1* shRNA significantly reduces BACE1 levels compared to scr controls set to 100% (*Bace1* ^*sh*RNA^: 7.86 ± 1.78%, *P* < 0.000, *N* = 4 biological replicates).U, VNumber of Aβ_1–42_ puncta is reduced in AP‐2μ KO neurons overexpressing the AP‐2μ‐mRFP, when compared to KO neurons expressing the mCherry (WT^mChery^: 0.49 ± 0.07, KO^mCherry^: 0.79 ± 0.10, WT^AP−2μ‐mRFP^: 0.54 ± 0.07, KO^AP−2μ‐mRFP^: 0.46 ± 0.06, pWT^mCherry^ versus pKO^mCherry^ = 0.026, pWT^mCherry^ versus pWT^AP−2μ‐mRFP^ = 0.963, pKO^mCherry^ versus pKO^AP−2μ‐mRFP^ = 0.008, pWT^AP−2μ‐mRFP^ versus pKO^AP−2μ‐mRFP^ = 0.840, 38‐40 neurons per condition, *N* = 4 biological replicates). Scale bar: 2 μm.Data information: All graphs show mean ± SEM; statistical analysis was performed by unpaired two‐tailed Student's *t*‐test in (D, E, J, M, R), two‐way ANOVA in (V), and one‐sample Student's *t*‐test in (H, K, T). Non‐normally distributed data (N,O) were transformed using square root transformation and analyzed using paired two‐tailed Student's *t*‐test. n.s.—non‐significant. * indicates *P* ≤ 0.05; ** indicates *P* ≤ 0.01; *** indicates *P* ≤ 0.001.

**Figure 4:**
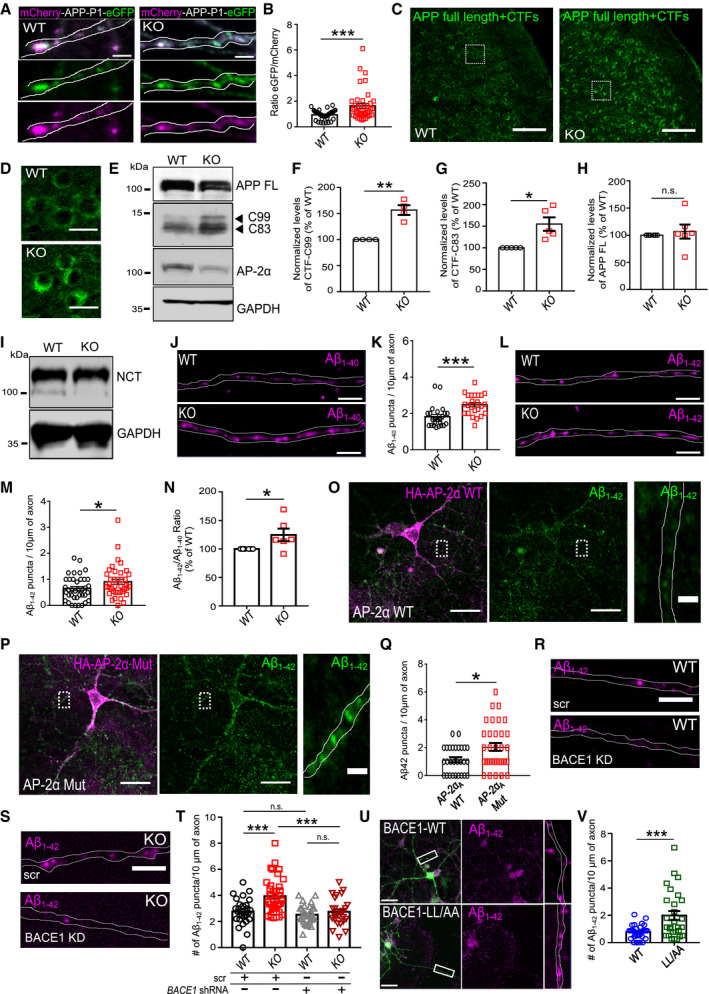
AP‐2 prevents amyloidogenic processing of APP in neurons A, BThe eGFP/mCherry signal intensity ratio is significantly increased in mCherry‐APP‐P1‐eGFP‐expressing KO neurons comparing to the WT (WT: 0.94 ± 0.06%, KO: 1.65 ± 0.18, *P* = 0.000, WT = 39 and KO = 41 neurons, *N* = 5 biological replicates). Scale bars: 2 μm.C, DConfocal images of WT and AP‐2μ KO entorhinal cortex immunostained with antibody recognizing total APP and APP‐CTFs. Scale bars: 100 μm (C), 20 μm (D).E–GLevels of APP‐CTFs are significantly increased in AP‐2μ KO cortex compared to the WT set to 100% (KO^C99^: 156.65 ± 8.37%, *P* = 0.004, *N* = 4 biological replicates; KO^C83^: 154.79 ± 15.6%, *P* = 0.012; *N* = 5 biological replicates).HLevels of full‐length (FL) APP are unaltered in AP‐2μ KO cortex (KO^APP FL^: 106.64 ± 12.96%, *P* = 0.315, *N* = 6 biological replicates).ILevels of Nicastrin (NCT) are unaltered in the AP‐2μ KO cortex (see also Fig EV4K).J, KIntracellular levels of Aβ_1–40_ are significantly increased in AP‐2μ KO axons compared to the WT (WT^Aβ1‐40^: 1.82 ± 0.12, KO^Aβ1–40^: 2.45 ± 0.12, *P* = 0.000, WT = 24 and KO = 24 neurons, *N* = 3 biological replicates). Scale bars, 5 μm.L, MIntracellular levels of Aβ_1–42_ are significantly increased in AP‐2μ KO axons (WT^Aβ1–42^: 0.65 ± 0.08, KO^Aβ1–42^: 0.91 ± 0.10, *P* = 0.046, WT = 38 and KO = 38 neurons, *N* = 3 biological replicates). Scale bar: 5 μm.NSignificantly increased ratio of Aβ_1–42_/Aβ_1–40_ measured by ELISA in the media of cultured AP‐2μ KO neurons compared to the WT set to 100% (KO:124.57 ± 10.97%, *P* = 0.037, *N* = 6 biological replicates).O–QAccumulation of intracellular Aβ_1–42_ in neurons overexpressing the AP2‐α‐Mut comparing to neurons expressing the AP2‐α–WT (WT: 1.14 ± 0.18, Mut: 2.06 ± 0.29, *P* = 0.011, WT = 28 and Mut = 33 neurons, *N* = 4 biological replicates). Scale bars: 20 μm, 2 μm (inserts).R–TAccumulation of intracellular Aβ_1–42_ is rescued upon *BACE1* knockdown in AP‐2μ KO neurons (WT^Scr^: 2.76 ± 0.20, KO^Scr^: 3.94 ± 0.21; WT^sh*BACE1*^: 2.52 ± 0.12; KO^sh*BACE1*^: 2.74 ± 0.21; pWT^Scr^ versus pKO^Scr^ = 0.000, pKO^scr^ versus pKO^sh*BACE1*^ = 0.000; pWT^scr^ versus pWT^sh*BACE1*^ = 0.826; pWT^sh*BACE1*^ versus pKO^sh*BACE1*^ = 0.863; 27–39 neurons per condition, *N* = 3 biological replicates). Scale bar: 10 μm.U, VLevels of intracellular Aβ_1–42_ are significantly upregulated in neurons overexpressing the HA‐BACE1‐LL/AA‐GFP comparing to neurons expressing the HA‐BACE1‐GFP (BACE1: 0.78 ± 0.90, BACE1‐LL/AA: 1.99 ± 0.33, *P* = 0.000, 28–29 neurons per condition, *N* = 3 biological replicates). Scale bar, 50 μm.Data information: All graphs show mean ± SEM; statistical analysis was performed by unpaired two‐tailed Student's *t*‐test in (B, K, M, N, Q, V), two‐way ANOVA in (T), and one‐sample Student's *t*‐test in (F‐H). n.s.—non‐significant. * indicates *P* ≤ 0.05; ** indicates *P* ≤ 0.01; *** indicates *P* ≤ 0.001.

Given the significant defect in transport and lysosomal targeting of BACE1 in AP‐2μ KO neurons, we next explored the functional importance of AP‐2 for amyloidogenic processing of APP. First, we tested whether BACE1 retains its β‐secretase activity in AP‐2μ KO neurons by taking advantage of Lys612Val mutant of APP (mCherry‐APP‐P1‐eGFP), which can be cleaved almost exclusively by BACE1 due to its inefficient cleavage by α‐secretase 44. This construct has been previously shown to be correctly expressed and targeted to the plasma membrane and intracellular organelles in neurons 45. Once BACE1 cleaves the mCherry‐containing N‐terminal domain of the APP, the fluorescence intensity ratio between the green and the red signals increases (Fig EV4A). By analyzing the eGFP/mCherry signal intensity ratio in WT and AP‐2μ KO neurons, we found that BACE1‐dependent APP cleavage was significantly higher in neurons lacking AP‐2μ (Figs 4A and B, and EV4B), indicating that stalled BACE1 retains its β‐secretase activity. These data, taken together with the fact that significantly more APP puncta contained BACE1 in AP‐2μ KO neurons (Fig EV4C–E), suggest that increased amount of intracellular BACE1 might facilitate APP processing in AP‐2‐deficient brains. In agreement with this hypothesis, increased levels of C‐terminal APP fragments (CTFs) were detected in brain sections from the entorhinal cortex of AP‐2 KO mice, while the levels of Reelin (used as a marker of entorhinal cortex layer II and a negative control) were unaltered (Figs 4C and D, and EV4F). Increased activity of BACE1 in brains lacking AP‐2μ was also confirmed by analyzing the cleavage of another BACE1 substrate L1 46, whose processing was significantly elevated in AP‐2 KO mice (Fig EV4G and H).

To precisely characterize the pathway responsible for increased CTF generation in AP‐2 KO brains, we quantified the processing of APP in brain lysates from neuronal‐confined AP‐2μ KO mice by Western blotting. In agreement with published data 44, we found that under physiological conditions, the majority of APP was cleaved by the α‐secretase pathway, with CTF 83 being the predominant form of APP C‐terminal fragments in WT cortical lysates (Fig 4E). Interestingly, in the cortex of AP‐2μ KO mice more APP was undergoing β‐secretase‐dependent cleavage, monitored by the levels of CTF 99 fragment (Fig 4F). These changes were, however, accompanied by an upregulation in CTF 83 (Fig 4G), likely as a consequence of increased α‐secretase cleavage due to slightly higher amounts of APP present on the surface of AP‐2μ KO neurons (Fig EV4I and J). This accumulation of APP‐CTFs was not a result of an increase in total APP levels (Fig 4H) and was unlikely mediated by the γ‐secretase, because no change in the level of γ‐secretase component Nicastrin was detected in AP‐2μ KO brains (Figs 4I and EV4K).

The inhibition of clathrin‐mediated APP endocytosis by mutation of its C‐terminally located internalization motif “YENPTY” increases soluble sAPPα secretion and reduces Aβ production 47. To investigate whether defective endocytosis of APP in AP‐2μ‐deficient neurons might result in a decrease in Aβ_1–40_ and Aβ_1–42_ peptide production, we monitored the levels of intracellular Aβ_1–40_ and Aβ_1–42_ in AP‐2μ KO axons using mouse monoclonal 12F4 and AB40.1 antibodies recognizing Aβ_1–42_ and Aβ_1–40_, respectively 48, 49. In contrary to the prediction above, we observed a significant rise of intracellular Aβ_1–40_ and Aβ_1–42_ peptides in neurons depleted for AP‐2μ (Fig 4J–M), a phenotype accompanied by a specific accumulation of Aβ_1–42_ within AP‐2 KO autophagosomes (Fig EV4L and M). These changes were paralleled by an increase in the ratio of extracellular Aβ_1–42_/Aβ_1–40_, likely due to a significant decrease in Aβ_1‐40_ secretion (Figs 4N and EV4N and O), strongly suggesting that AP‐2 functions not only upstream at the APP endocytosis level, but also downstream of the production of C99. With its ability to bind and traffic BACE1 toward lysosomal organelles (Figs 2 and 3), we next asked whether AP‐2 mediates Aβ generation via BACE1‐dependent pathway. Overexpression of fully endocytosis‐competent LC3 binding‐deficient mutant of AP‐2α_A_ 32 in control neurons produced an increase in BACE1 protein levels and a concomitant upregulation of Aβ_1–42_ peptide (Figs EV4P–R and 4O–Q,). On the other hand, shRNA‐mediated *Bace1* knockdown (KD) (Fig EV4S and T) significantly reduced Aβ_1–42_ peptide levels in AP‐2μ KO neurons, indicating that elevated levels of BACE1 in KO condition were directly responsible for increased amyloidogenic processing of APP (Fig 4R–T). Of note, Aβ_1–42_ peptide levels were not significantly altered by BACE1 KD in WT neurons. This is in agreement with a small effect of BACE1 KO on CTF99 levels 50, likely due to insensitivity of conventional protein detection techniques in analyzing the Aβ_1–42_ picogram range changes in the control condition 51. The Aβ_1–42_ peptide accumulation in AP‐2 KO neurons was due to the lost interaction of BACE1 with the AP‐2, since elevated Aβ_1–42_ levels were detected in control neurons overexpressing AP‐2 binding‐deficient mutant of BACE1 (LL/AA) (Fig 4U and V) and were rescued upon re‐expression of AP‐2μ in AP‐2 KO neurons (Fig EV4U and V). Collectively, these data indicate that AP‐2 regulates BACE1 trafficking in neurons to prevent amyloidogenic processing of APP.

### Downregulation of BACE1 rescues amyloidogenesis and mitigates synapse loss in AP‐2 KO neurons

**Figure 5:**
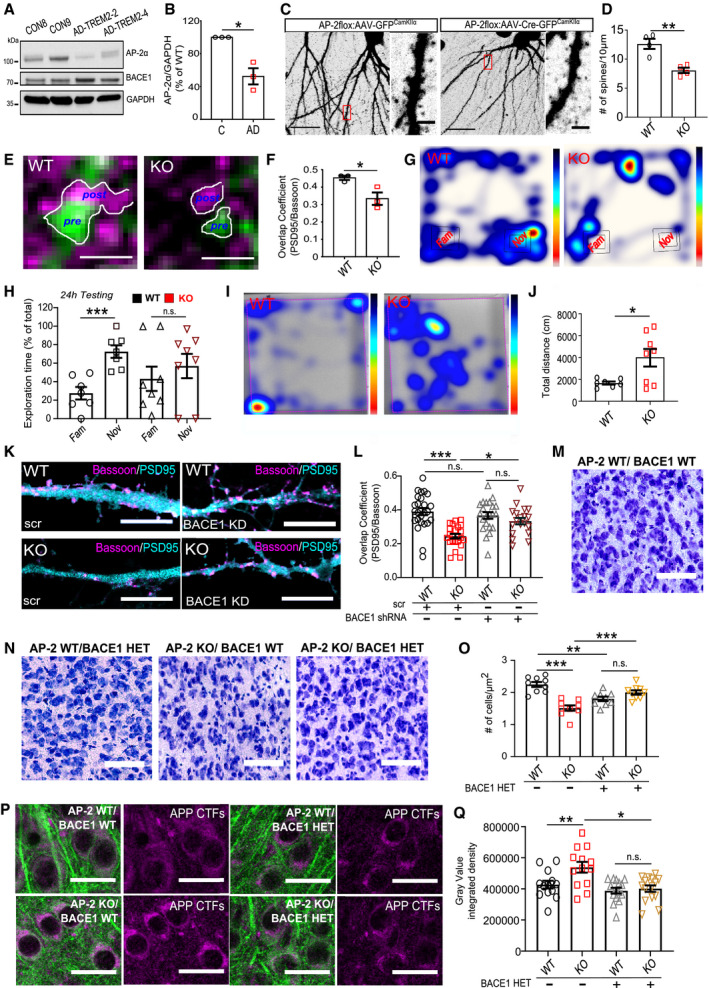
Synaptic dysfunctions in AP‐2μ KO mice are mitigated by BACE1 haploinsufficiency. A, BAP‐2α levels normalized to GAPDH are significantly decreased in lysates of iPSC‐derived neurons from late‐onset AD patients carrying the *TREM2 p.R47H* (AD‐*TREM2*‐2 and AD‐*TREM2*‐4) variant compared to healthy controls (CON8 and CON9) set to 100% (AD^AP−2α^: 52.42 ± 8.04%, *P* = 0.020, *N* = 3 biological replicates).CConfocal images of either AP‐2 WT (AAV‐GFP^CamkIIα^‐labeled) or AP‐2 KO (AAV‐Cre‐GFP^CamkIIα^‐labeled) granule neurons in the dentate gyrus of 13‐week‐old mice. Scale bars: 20 μm, 2 μm (inserts).DLoss of AP‐2μ significantly decreases the number of spines on granule neurons (WT: 12.63 ± 0.88, KO: 8.07 ± 0.45, *P* = 0.004, *N* = 4 biological replicates).E, FOverlap coefficient between the presynaptic marker Bassoon and the postsynaptic marker PSD95 is significantly decreased in the entorhinal cortex of AP‐2μ KO mice compared to the WT (WT: 0.45 ± 0.02, KO: 0.33 ± 0.06, *P* = 0.034, *N* = 3 biological replicates). Scale bar, 750 nm.G, HLost preference for the novel object (nov) comparing to the familiar object (fam) in AP‐2μ KO mice tested 24 h after training in NOR (WT^fam^: 27.51 ± 6.77, WT^nov^: 72.49 ± 6.77, pWT^fam^ versus WT^nov^ = 0.000; KO^fam^: 43.9 ± 13.12, KO^nov^: 59.91 ± 13.12, pKO^fam^ versus KO^nov^ = 0.469, *N* = 7 WT and 8 KO mice). Representative heatmaps are shown in (G).I, JAP‐2μ KO mice are significantly more active in the open field compared to the WT (WT: 1663 ± 139.08%, KO: 3976 ± 802.30%, *P* = 0.020, *N* = 7 WT and 8 KO mice). Representative heatmaps are shown in (I).K, LOverlap coefficient between Bassoon and PSD95 is rescued upon *BACE1* KD in AP‐2μ KO neurons (WT^scr^: 0.39 ± 0.02; KO^scr^: 0.24 ± 0.01; WT^sh*BACE1*^: 0.37 ± 0.02; KO^sh*BACE1*^: 0.33 ± 0.02; pWT^scr^ versus pKO^scr^ < 0.000; pKO^scr^ versus pKO^sh*BACE1*^ = 0.010; pWT^scr^ versus pWT^sh*BACE*1^ = 0.813; pWT^sh*BACE1*^ versus pKO^sh*BACE1*^ = 0.623, 22–24 neurons for each condition, *N* = 3). Scale bars: 10 μm.M–ONeuronal loss is rescued in the entorhinal cortex of AP‐2μ KO mice haploinsufficient for BACE1 (AP‐2^WT^/BACE^WT^: 2.25 ± 0.08, AP‐2^KO^/BACE^WT^: 1.51 ± 0.08, AP‐2^WT^/BACE^HET^: 1.80 ± 0.07, AP‐2^KO^/BACE^HET^: 2.00 ± 0.07; p^AP−2WT/BACE1WT^ versus p^AP−2KO/BACE1WT^ < 0.000_;_ p^AP−2WT/BACE1WT^ versus p^AP−2WT/BACE1HET^ = 0.001; p^AP−2KO/BACE1WT^ versus p^AP−2KO/BACE1HET^ = 0.000; p ^AP−2WT/BACE1HET^ versus ^AP−2KO/BACE1HET^ = 0.268, *N* = 9 sections from *N* = 3 for each genotype). Scale bar: 100 μm.P, QAPP‐CTF levels are rescued in the entorhinal cortex of AP‐2μ KO mice haploinsufficient for BACE1 (AP‐2^WT^/BACE^WT^:427574 ± 24021, AP‐2^KO^/BACE^WT^: 538895 ± 34662, AP‐2^WT^/BACE^HET^: 389870 ± 19842, AP‐2^KO^/BACE^HET^: 395389 ± 21853; p^AP−2WT/BACE1WT^ versus p^AP−2KO/BACE1WT^ = 0.017_;_ p^AP−2KO/BACE1WT^ versus p^AP−2KO/BACE1HET^ = 0.001; p ^AP−2WT/BACE1HET^ versus ^AP−2KO/BACE1HET^ = 0.979; 13–15 sections, *N* = 3 mice for each genotype). Scale bars: 40 μm.Data information: All graphs show mean ± SEM; statistical analysis was performed by one‐sample Student's *t*‐test in (B), unpaired two‐tailed Student's *t*‐test in (D, F, H, J) and two‐way ANOVA in (L, O, Q). n.s.—non‐significant.* indicates *P* ≤ 0.05; ** indicates *P* ≤ 0.01; *** indicates *P* ≤ 0.001.

**Figure EV5:**
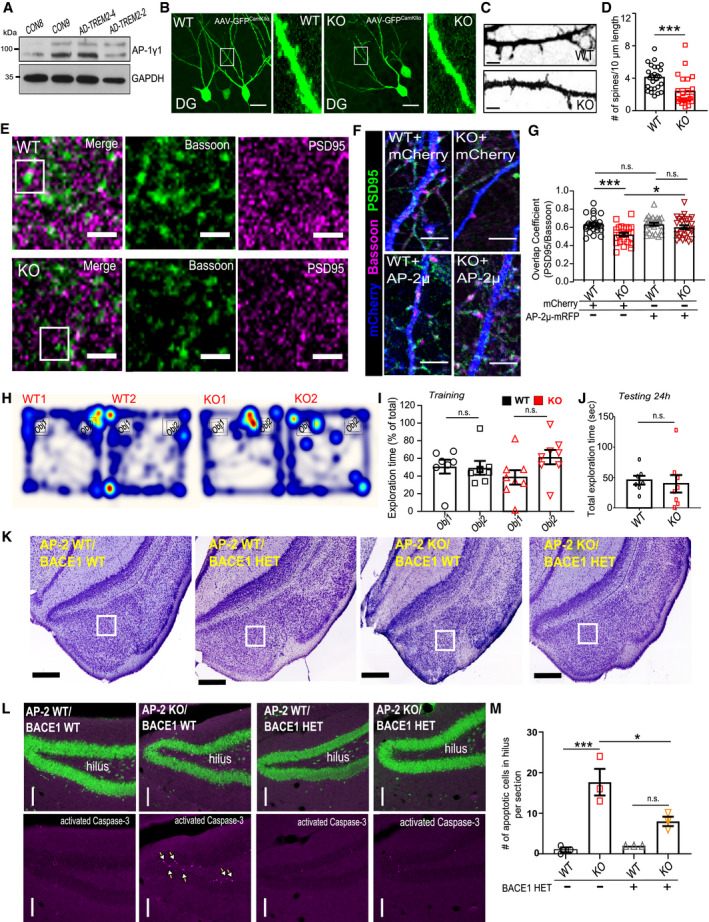
AP‐2‐dependent BACE1 trafficking is required to prevent the neurodegeneration in the brain AAP‐1γ1 levels in lysates of iPSC‐derived neurons from late‐onset AD patients carrying the *TREM2 p.R47H* (AD‐*TREM2*‐2 and AD‐*TREM2*‐4) variant compared to healthy controls (CON8 and CON9).BConfocal images of AAV‐GFP^*CamKIIα*^ *‐*labeled WT and AP‐2μ KO dentate gyrus (DG) granule cells. Scale bars: 20 and 5 μm.C, DDendritic spine number is significantly reduced in eGFP‐expressing AP‐2μ KO‐cultured neurons when compared to the WT (WT: 4.08 ± 0.30, KO: 2.36 ± 0.38, *P* = 0.000), 26 WT and 24 KO neurons, *N* = 3 biological replicates. Scale bar: 2 μm.EOverview images of WT and AP‐2μ KO entorhinal cortex shown in Fig 5E. Scale bar: 5 μm.FRepresentative images of dendritic fragments of WT and AP‐2μ KO neurons either expressing mCherry or AP‐2μ‐mRFP and immunostained for PSD95 and Bassoon. Scale bar, 5 μm.GOverlap coefficient between Bassoon and PSD95 is increased in AP‐2μ KO neurons expressing the AP‐2μ‐mRFP, compared to KO neurons expressing the mCherry (WT^mChery^: 0.63 ± 0.02, KO^mCherry^: 0.52 ± 0.02, WT^AP−2‐μ‐mRFP^: 0.63 ± 0.02, KO^AP−2‐μ‐mRFP^: 0.60 ± 0.02, pWT^mCherry^ versus pKO^mCherry^ = 0.0003, pWT^mCherry^ versus pWT^AP−2‐μ‐mRFP^>0.999, pKO^mCherry^ versus pKO^AP−2‐μ‐mRFP^ = 0.015, pWT^AP−2‐μ‐mRFP^ versus pKO^AP−2‐μ‐mRFP^ = 0.609, 26–27 neurons per condition, *N* = 4 biological replicates).HRepresentative heatmaps, showing the position of the WT and AP‐2μ KO mouse in the experimental arena in the NOR training session.INo significant difference in the exploration time toward neither object 1 (Obj 1) nor Obj 2 was observed for WT and AP‐2μ KO mice during the training period (WT^obj1^: 50.71 ± 7.34, WT^obj2^: 49.29 ± 7.85, pWT^obj1^ versus WT^obj2^ = 0.901; KO^obj1^: 38.48 ± 8.12, KO^obj2^: 61.52 ± 8.68, pKO^obj1^ versus KO^obj2^ = 0.065; 7 WT and 8 KO mice).JTotal exploration time in NOR test is not altered in AP‐2μ KO mice (WT: 45.55 ± 7.24%, KO: 39.63 ± 14.48%, *P* = 0.733, 7 WT and 8 KO mice).KNissl‐stained brain sections of WT and AP‐2μ KO mice shown in Fig 5M and N. Scale bars: 500 μm. White boxes indicate the area magnified in Fig 5M and N.LRepresentative confocal images of WT and AP‐2μ KO brains haploinsufficient for BACE1 immunostained for activated caspase‐3 and NeuN. Scale bar: 200 μm.MThe number of apoptotic cells is reduced in the hilus of AP‐2μ KO mice haploinsufficient for BACE1 (AP‐2^WT^/BACE^WT^: 1.00 ± 0.58, AP‐2^KO^/BACE^WT^: 17.67 ± 3.28, AP‐2^WT^/BACE^HET^: 2.00 ± 0, AP‐2^KO^/BACE^HET^: 8.00 ± 1.54; p^AP−2WT/BACE1WT^ versus p^AP−2KO/BACE1WT^ = 0.000_;_ p^AP−2KO/BACE1WT^ versus p^AP−2KO/BACE1HET^ = 0.020; p ^AP−2WT/BACE1HET^ versus ^AP−2KO/BACE1HET^ = 0.153). Three sections from *N* = 3 mice for each genotype.Data information: All graphs show mean ± SEM; statistical analysis was performed by unpaired two‐tailed Student's *t*‐test in (D, J, I) and two‐way ANOVA in (G, M). n.s.—non‐significant. * indicates *P* ≤ 0.05; ** indicates *P* ≤ 0.01; *** indicates *P* ≤ 0.001.

Accumulations of Aβ are a hallmark of AD, and a recent transcriptome‐wide association study identifies AP‐2α subunits as late‐onset AD‐associated genes 52. Since decreased levels of AP‐2α, but not the AP‐1γ1, were detected in iPSC‐derived neurons from patients with late‐onset AD (Figs 5A and B, and EV5A), we next asked whether increased amyloidogenic processing of APP in neurons lacking AP‐2 is relevant for AD‐associated synaptic pathology. Previously, we have shown that AP‐2 is not required for brain development and functions to maintain the neuronal complexity 32. To directly address the role of AP‐2 in spine morphology *in vivo*, we adopted the AAV‐mediated gene KD approach. To this aim, we stereotactically delivered either control AAV‐GFP^CamKIIα^ vector or vector expressing Cre‐GFP^CamKIIα^ into the dentate gyrus of 9‐week‐old AP‐2μlox/lox mice. We observed that 4 weeks postinjection, AAV‐Cre‐GFP^CamKIIα^‐expressing granule neurons possessed significantly less dendritic spines, suggesting that AP‐2 regulates synaptic density in mature brain (Fig 5C and D). In fact, dendritic spine density was also reduced in granule cells of neuronal‐confined AP‐2 KO (*Ap2m1* ^lox/lox^: *Tubulin 1α* Cre) mice described earlier (Fig EV5B) and in primary hippocampal–cortical KO neurons (Fig EV5C and D). This phenotype was accompanied by a significant reduction in the co‐localization of pre‐ and postsynaptic markers (Figs 5E and F, and EV5E) and was specific to AP‐2 loss, since the re‐expression of AP‐2μ restored synaptic density in AP‐2μ KO neurons (Fig EV5F and G). Next, we asked whether the loss of AP‐2μ in neurons causes cognitive dysfunction akin to changes reported for transgenic AD mouse models 53 and human patients 54. We performed the novel object recognition task, which is widely used to assess the recognition memory decline in mice 55. During the training session, both control and KO mice did not show a preference for either of two identical objects (Fig EV5H and I). Twenty‐four hours later, control animals clearly favored the novel object compared to the familiar object, while AP‐2μ KO mice showed a significantly decreased preference toward the novel object, an indicator of impaired memory on novelty preference (Figs 5G and H, and EV5J). Remarkably, AP‐2μ KO mice were also significantly more active in the open field (Fig 5I and J), in agreement with the reported hyperactivity of AD mouse models 56, 57.

Is increased amyloidogenic processing of APP due to the elevated levels of BACE1 directly responsible for synaptic dysfunctions observed in AP‐2μ KO mice? To answer this question, we used the shRNA‐mediated *Bace1* knockdown (KD) approach. We found that reduced synaptic density (Fig 5K and L) was rescued in AP‐2μ KO neurons deficient for BACE1. Lastly, to study the role of BACE1‐mediated APP processing in AP‐2μ KO brains *in vivo*, we quantified the cell number and levels of APP‐CTFs in the entorhinal cortex of AP2‐2μ KO mice haploinsufficient for BACE1. Strikingly, BACE1 haploinsufficiency was enough to prevent the neurodegeneration, cell death, and APP‐CTF accumulation in AP‐2μ KO brains (Figs 5M–Q and EV5K–M). These results confirm our observations from cultured AP‐2μ KO neurons and suggest that neuronal AP‐2 prevents amyloidogenesis via regulating the intracellular levels of BACE1 in the brain.

**Figure 6:**
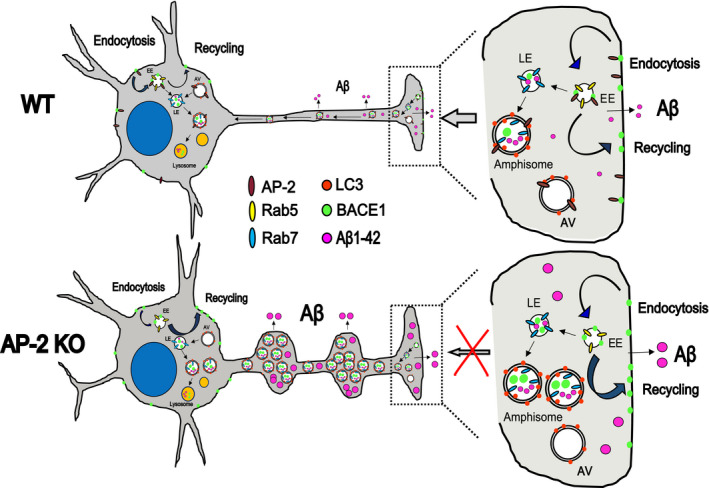
Hypothetical model explaining the role of AP‐2 in intracellular trafficking of BACE1 In WT neurons, AP‐2μ is required for transport of BACE1‐containing autophagosomes and late endosomes *en route* to lysosomes. Absence of AP‐2μ causes defective axonal trafficking of BACE1 leading, which results in increased amyloidogenic processing of APP.

Taken together, our results reveal a novel function for AP‐2 in the regulation of APP processing and Aβ generation in neurons via promoting the intracellular trafficking and degradation of BACE1 (Fig 6). This function of AP‐2 is independent of its role in BACE1 endocytosis and requires its association with LC3‐containing autophagosomes, suggesting a combined pharmacological targeting of endocytic adaptors and autophagy as a new therapy for reducing the levels of Aβ in the AD brain.

## Discussion

Our data reported here reveal an unexpected function for endocytic adaptor protein AP‐2 in neurons, where it functions as a critical regulator of amyloidogenesis. We show that AP‐2 controls neuronal APP processing by regulating the intracellular trafficking of BACE1. Loss of AP‐2μ causes stalling of BACE1‐containing autophagosomes in axons, a phenotype that facilitates the intracellular generation of Aβ_1–40_ and Aβ_1–42_.

Neuronal loss in AD is accompanied by the progressive accumulation of Aβ 1 and the presence of autophagosomes 58. In neurons, unlike non‐neuronal cells, the formation of autophagosomes and their turnover are spatially segregated, since autophagosomes are generated in distal axons and retrogradely transported along microtubules to the cell soma, which contains the majority of lysosomes 59. Neuronal autophagy is regulated by a variety of cues, including endocytic adaptor proteins 60, which supply the plasma membrane for autophagosome formation 61 and regulate autophagosomal transport 32. However, whether autophagosome trafficking mediated by endocytic adaptors regulates Aβ levels in the brain was previously unknown. Here, we report that endocytic adaptor AP‐2 prevents amyloidogenic processing of APP via regulating *en route* trafficking of BACE1 toward lysosomes. Our data are in agreement with several studies indicating autophagosomes as an alternative site of Aβ generation in neurons 58, 62, 63 and suggest that autophagosome stalling observed in AD mouse models and human patients might be responsible for Aβ deposition in the brain.

Our results support the hypothesis according to which dysfunctional endosomal trafficking might underlie the earliest pathology of AD. Indeed, the presence of enlarged early endosomes precedes the Aβ peptide inclusions in patients with sporadic late‐onset AD 64, 65, while substantial number of the newly identified AD risk loci encodes proteins that function predominantly in endocytic trafficking, including PICALM, BIN1, and SORL1 66. To date, only a few studies examined the precise role of endocytic proteins in AD 33, 67, 68, and whether abnormal endosomal recycling in late‐onset AD patients is a consequence of endocytic protein loss‐of‐function is currently unknown. We find that the endocytic complex AP‐2, a clathrin adaptor, previously shown to be not essential for CME in neurons 30, 31, but to function in synaptic vesicle reformation 30 and autophagosome transport 32, regulates intracellular trafficking of BACE1. We show that endocytic adaptor AP‐2 is downregulated in iPSC‐derived neurons from AD patients with carrying the *TREM2* ^R47H^ risk variant, which is one of the strongest single allele genetic risk factor for sporadic late‐onset AD 69. These results taken together with a recent finding, revealing endocytosis among seven most downregulated gene sets in the hippocampus of late‐onset AD *TREM2* ^R47H^ patients [preprint: 70], suggest an existing hypothesis that *TREM2* ^R47H^‐induced immune response in microglial cells might transcriptionally regulate AP‐2 levels in brains of AD patients. Our data demonstrate that AP‐2 prevents Aβ generation by controlling the degradation of endosomal BACE1 and this function of AP‐2 is of particular relevance for recognition memory in mice which is akin to the phenotype described for sporadic AD patients 54. Our results are in line with a recent transcriptome‐wide association study, which identifies both α subunits of the AP‐2 complex as late‐onset AD‐associated genes 52. Together with an earlier report indicating decreased expression levels of AP‐2α in the AD brain 71, our findings suggest that AP‐2 changes observed in *TREM2* ^R47H^‐carrying AD patients could partially be responsible for increased amyloidogenic processing of APP in the AD brain. However, further studies are needed to better delineate whether there is a relationship between *TREM2* ^R47H^‐induced inflammatory response, AP‐2 levels, and neuritic plaque deposition in human AD patients.

Several lines of evidence suggest that the role of AP‐2 in trafficking of BACE1 is independent of its established role in endocytosis and requires the recently described interaction of AP‐2 with autophagosomes 32. First, we find that AP‐2 is not essential for BACE1 endocytosis, but is required for its endosomal delivery to lysosomes (Figs 1 and 2). Second, AP‐2 is associated and co‐trafficked with BACE1‐positive carriers in axons (Fig 3). Third, we show that defective BACE1 trafficking is phenocopied by overexpression of LC3 binding‐deficient AP‐2α_A_ mutant (Fig 3), which is fully functional with respect to endocytosis 32. Finally, in contrary to previously published data indicating the benefit of clathrin‐mediated endocytosis inhibition in AD 72, 73, we find that AP‐2 deletion in neurons facilitates amyloidogenic APP processing and causes synaptic dysfunction (Figs 4 and 5). Our findings further strengthen the endocytosis‐independent role for endocytic adaptors in neurons 32, 33, 74, 75 and suggest that extremely polarized morphology of neuronal cells, where endocytic cargo has to be transported over long distances, might largely be responsible for the brain‐confined phenotype of AD patients at the initial stages of the disease 76. In fact, since in *C. elegans* AP‐2 has been reported to control the anterograde delivery of glutamate receptor GLR1 to the plasma membrane 77, our data suggest that the generalized defect of AP‐2 loss on BACE1 trafficking might be due to additional, yet undescribed AP‐2 functions in the secretory pathway in mammalian neurons.

Based on our data, the function of AP‐2 in APP processing requires BACE1 endocytosis from the plasma membrane. The endocytosis of BACE1 is known to depend on the DISLL sequence located in its cytosolic tail 22, a sorting signal recognized by a variety of the adaptor proteins 78, including AP‐2 23. In the absence of the di‐leucine motif, BACE1 is recycled from the endosomal system to the cell surface at twice its normal rate 22, a phenotype also observed in AP‐2 KO neurons in the current study. Thus, our results agree with the hypothesis that AP‐2 is not required for BACE1 endocytosis (in agreement with Ref. 25), but instead regulates the trafficking of BACE1 toward degradation compartments and its absence facilitates its recycling back to the plasma membrane. The AP‐2‐dependent sorting of BACE1 toward lysosomes is consistent with previously described role for AP‐2 in clathrin coat assembly on these organelles 79. We suggest that the function of AP‐2 in BACE1 degradation and APP processing is of a special importance in neurons, where the synaptic activity triggers the convergence of usually distinctly located APP and BACE1 into amyloidogenic acidic microdomains 21. This phenomenon can explain why AP‐2‐dependent amyloidogenesis might have been overlooked in the past 23. In fact, recent data highlight BACE1 enrichment in synaptic vesicles 80, a presynaptic organelle, which is regenerated upon neuronal activity in AP‐2‐dependent manner 30. Thus, our data propose a model where AP‐2 functions at presynaptic membrane compartments to sort BACE1 to late endosomes and autophagosomes, thus regulating its degradation during neuronal activity. Lack of AP‐2 could trigger a positive feedback loop between BACE1 internalization and Aβ production in highly active neurons, which in turn might lead to hyperexcitability due to intrinsic Aβ effect on expression of voltage‐gated sodium channels 81. Whether the degradation route of the APP is also directly mediated by the AP‐2 remains to be determined, although autophagy‐mediated degradation of APP‐CTFs dependent on the neuronal‐enriched AP‐2α_A_ has previously been reported 82. Furthermore, a recent study revealed that neurotoxic Aβ levels in microglia are non‐canonically regulated by autophagy, which mediates the LC3 recruitment to Aβ‐containing clathrin‐positive endosomes 83. Irrespective of the precise mechanisms involved, results presented here identify a previously undescribed role for AP‐2 in regulation of amyloidogenic processing of APP in neurons and lay the groundwork for the identification of novel therapeutic targets in AD.

## Materials and Methods

### Animals

C57/BL/6 mice were housed in polycarbonate cages at standard 12/12 day–night cycles, and water and food were provided *ad libitum*. All animal experiments were approved by the ethics committee of LANUV Cologne and were conducted according to the committee's guidelines. Conditional tamoxifen‐inducible (*Ap2m1* ^lox/lox^ × inducible CAG‐Cre) and neuron‐confined (*Ap2m1* ^lox/lox^ × *Tubulin1α‐*Cre) AP‐2μ KO mice, as well as conditional tamoxifen‐inducible ATG5 KO (*Atg5* ^flox/flox^: B6.Cg‐Tg(CAG‐Cre/Esr1*)5Amc/J), were described previously 32. Neuron‐confined AP‐2μ KO mice were crossbred with BACE1 KO mice (*Bace1* ^*tm1Pcw*^, Jackson Laboratory, Stock #: 004714) to obtain the AP‐2μ KO: BACE1 HET mice.

### Stereotaxic injection of AAV9‐eGFP

Stereotactic injections were performed either on 14‐day‐old WT and *Ap2m1* ^lox/lox^ x *Tubulin1α*‐Cre mice or on 9‐week‐old *Ap2m1* ^lox/lox^ mice. Mice were anesthetized with a mixture of ketamin (100 mg/kg)/xylazin (20 mg/kg)/acepromazine (3 mg/kg) and mounted in the Kopf stereotactic frame. Local anesthetic was applied before opening of the scalp. A small craniotomy was performed above the point of injections. For 9‐week‐old animals, 200 nl of AAV9*‐*eGFP^*CamKIIα*^ or AAV9*‐*Cre*‐*eGFP^*CamKIIα*^ was injected with a Hamilton syringe into the dentate gyrus (AP—3.5; ML—2.5; DV—2.5) with a 5‐min delay after the penetration, and waiting another 15 min before withdrawing of the syringe. For the injections in 14‐day‐old animals, a1 μl Hamilton syringe filled with 300 nl of AAV9*‐*eGFP^*CamKIIα*^ was lowered into the thalamus, using following coordinates: AP—1.05; DL—1.32; depth—3.00. A volume of 150 nl AAV was delivered during 5 min, with a 5‐min delay after the penetration, and waiting another 15 min before withdrawing of the syringe. The animal was given a dose of carprofen to reduce postsurgical pain before the end of the surgery. Suturing the skin over the wound completed the surgery, and the animal was allowed to recover. Mice were sacrificed by transcardial perfusion either 5–7 days after the surgery for 14‐day‐old animals or 4 weeks after the surgery for 9‐week‐old animals, and eGFP expression was analyzed by confocal microscopy (see Immunohistochemical analysis of brain sections).

### Preparation of primary neurons and transfections

Primary neurons were isolated from cortex and hippocampus of p1‐5 mice, according to the previously described protocol 84. Homologous recombination of conditional AP‐2μ KO allele was induced by the addition of 0.4 μM (Z)‐4‐hydroxytamoxifen (Sigma) immediately after plating. During medium renewal after 1 and 24 h, cells were treated with 0.2 and 0.4 μM of tamoxifen, respectively. Ethanol was added to control neurons (WT) in an equal amount as tamoxifen. For most of the experiments, neurons were transfected at DIV 7–8 using optimized calcium phosphate protocol described previously 84 and always analyzed at DIV 10‐12.

### Immunocytochemistry on cultured neurons

Neurons were fixed on DIV 11–12 in 4% paraformaldehyde (PFA) in phosphate‐buffered saline (PBS containing 137 mM NaCl, 2.7 mM KCl, 10 mM Na_2_HPO_4_, 1.8 mM, K_2_HPO_4_, pH 7.4) for 15 min at room temperature (RT), washed three times with PBS, and blocked for 1 h at RT with blocking buffer containing 0.3% saponin (SERVA Electrophoresis GmbH) and 5% normal goat serum (NGS) in PBS. Neurons were then incubated with primary antibodies (Table EV1) in the blocking buffer for 1 h at RT. Coverslips were rinsed three times with PBS (5 min each) and incubated with corresponding secondary antibodies (Table EV1) for 30–60 min at RT in blocking solution. Subsequently, coverslips were washed three times in PBS and mounted in Immu‐Mount (Thermo Scientific).

### Kinetics of BACE1 endocytosis

To analyze the kinetics of BACE1 endocytosis, WT and AP‐2μ KO neurons were transfected with either HA‐BACE1‐eGFP or BACE1‐eGFP plasmids at DIV 7–8. At DIV 11, neurons were incubated with the HA antibody for 30 min (pulse) at 4°C in osmolarity‐adjusted NBA medium to label the surface BACE1. After that, the cells were transferred to CO_2_ incubator at 37°C and kept for 5, 20, or 40 min to allow the endocytosis of HA antibody‐bound BACE1 (chase). Afterward, the neurons were acid‐stripped (0.5 M NaCl, 0.2 M acetic acid) for 4 s 33 to remove the surface‐bound non‐endocytosed HA antibody, washed two times with PBS, and fixed and immunostained under permeabilizing condition as described above. The levels of internalized BACE1 were quantified by determining the fluorescence ratio of the HA signal (endocytosed BACE1) to the eGFP expression (total BACE1) in WT and KO conditions.

### BACE1 pulse‐chase assay

To label the internalized fraction of BACE1, WT and AP‐2μ KO neurons, transfected either with HA‐BACE1‐eGFP or with HA‐BACE1‐mCherry plasmids and co‐transfected either with eGFP, eGFP‐RAB4, eGFP‐RAB4S22N, eGFP‐RAB11 or eGFP‐RAB11S25N (see Table EV2), were first incubated with the HA antibody (pulse) in osmolarity‐adjusted NBA medium in CO^2^ incubator at 37°C for 20 min. Afterward, the neurons were acid‐stripped (0.5 M NaCl, 0.2 M acetic acid) for 4 s 33 to remove the surface‐bound non‐endocytosed HA antibody and washed two times with PBS. Following the stripping, neurons were either fixed with 4% PFA (= internalized BACE1 fraction) or kept for 20 min (chase) at 37°C in the CO2 incubator to allow the recycling of BACE1 back to the plasma membrane (= recycled BACE1 fraction) and subsequently fixed in 4% PFA. To visualize the internalized BACE1 fraction, fixed neurons were incubated in blocking buffer containing 0.3% saponin and 5% NGS in PBS and immunostained as described above. To reveal the recycled BACE1 fraction, fixed neurons were first blocked with 10% NGS in PBS (non‐permeabilizing buffer) for 1 h at RT, rinsed three times with PBS, incubated with secondary antibody in PBS for 30 min, washed three times with PBS, and mounted using Immu‐mount. The levels of internalized or recycled BACE1 were quantified by determining the fluorescent ratio of the HA signal (internalized or recycled BACE1) to the eGFP or mCherry expression (total BACE1) in WT and KO conditions (see details under Analysis of immunofluorescence). For surface analysis of APP, WT and AP‐2μ KO neurons overexpressing mCherry‐APP‐eGFP were fixed and immunostained under non‐permeabilizing condition (10% NGS in PBS) using mCherry antibody. The surface levels of APP were quantified by determining the mCherry (surface)/eGFP (total) fluorescence ratio for individual neurons.

### Surface fraction analysis of GABARB3 receptors

WT and AP‐2μ KO neurons were fixed with 4% PFA and blocked with 10% NGS in PBS followed by immunostaining with surface GABARB3 antibody (recognizing the N‐terminus, see Table EV1) under non‐permeabilizing condition. Following the surface staining, neurons were postfixed with 2% PFA on ice and permeabilized with 0.1% Triton‐X in PBS. Neurons were incubated in blocking buffer containing 0.3% Triton‐X and 10% NGS in PBS. Following blocking, neurons were immunostained for antibody to detect the total GABARB3 (recognizing the C‐terminus, see Table EV1).

### Analysis of immunofluorescence

Neurons were analyzed using Zeiss Axiovert 200M microscope equipped with 63×/1.4 oil DIC objective and the Micro‐Manager software (Micro‐Manager1.4, USA). For quantitative analysis, the area of the neuron, including the cell body and the axon, was manually selected using the ImageJ selection tools (ROI Manager) and the mean gray value was quantified within the ROI after the background subtraction. To quantify the levels of endocytosed and recycled BACE1, the HA signal (endocytosed or recycled fractions) and the GFP or mCherry signals (total levels of BACE1 expression) were individually quantified for each neuron. Next, the ratio of HA immunofluorescence to either eGFP or mCherry was generated for WT and KO conditions. To quantify the surface levels of GABARB3, the surface and total GABARB3 levels were individually quantified for each neuron and a ratio of surface to total was generated for WT and KO conditions. Axons were identified based on the distinct morphology, and the percentage of co‐localization between BACE1 and LC3, APP, or RAB7 in cultured neurons was manually quantified for different axonal fragments. The percentage of co‐localization between BACE1 and Cathepsin D was quantified in the soma. Co‐localization between endogenous BACE1/RAB5 and BACE1/AP‐2α was determined by Pearson's correlation coefficient using ImageJ Coloc 2 plugin. Aβ puncta were manually quantified for each axonal fragment and represented as a number of puncta per 10 μm of axonal length. To determine the levels of Aβ in the autophagosomes, first the axonal autophagosomes were selected as region of interest (ROI) and the amount of Aβ was quantified within the selected ROI. For the mCherry‐APP‐P1‐eGFP cleavage assay, mean gray values of mCherry and GFP signals were quantified separately and the ratio of eGFP/mCherry was determined.

### Analysis of the spine density

Cultured WT and AP‐2μ KO neurons were transfected with eGFP at DIV 7–8 to visualize the neuronal morphology and fixed between DIV 11–12 (the latest possible time point before occurrence of cell death in AP‐2 KO neurons, Kononenko *et al*, 2017). To quantify the spine number, 2–3 small ROIs were selected along the primary dendrites for each neuron. The number of spines was quantified manually per 10 μm of dendritic length. Similar analysis was also performed with *in vivo* samples.

### Aβ rescue experiments using BACE1 knockdown in cultured neurons

WT and AP‐2μ KO neurons were transfected with eGFP and co‐transfected either with BACE1 shRNA‐NLS‐RFP (Tables EV2 and EV3, 85) or with scrambled (scr) shRNA‐NLS‐RFP at DIV 7. At DIV 11–12, the neurons were fixed in 4% PFA in PBS for 15 min at RT, washed three times, and then permeabilized and blocked for 1 h with the solution containing 5% NGS and 0.1% saponin (blocking buffer). Next, the neurons were incubated with the primary antibody (Aβ42, Table EV1), diluted in the blocking buffer for 1 h at RT. Following the incubation, the coverslips were rinsed four times with PBS (2–5 min each) and incubated with Alexa 647‐conjugated secondary antibody (1:500) in the blocking buffer for 30 min. Subsequently, the coverslips were washed three times in PBS and mounted using Immu‐mount. Fluorescence images were acquired using Zeiss Axiovert 200M microscope equipped with 63×/1.4 oil DIC objective and the Micro‐Manager software (Micro‐Manager1.4, USA). Transfected neurons were identified by co‐expression of eGFP and NLS‐RFP. To quantify the number of Aβ puncta, 30‐ to 40‐μm‐long axonal fragment was derived from individual neuron using ImageJ ROI manager plugin. Aβ puncta were manually quantified from each fragment per 10 μm of axonal length.

### Aβ rescue experiments upon AP‐2 overexpression in cultured neurons

WT and AP‐2μ KO neurons were first transfected with mCherry or AP‐2μ‐mRFP at DIV 6. At DIV 11–12, the neurons were fixed in 4% PFA in PBS for 15 min at RT and immunostained with primary antibody against Aβ42 as described above. Fluorescence images were acquired using Zeiss Axiovert 200M microscope equipped with 63×/1.4 oil DIC objective and the Micro‐Manager software (Micro‐Manager1.4, USA). Transfected neurons were identified by the expression of mCherry or mRFP. To quantify the number of Aβ puncta, 30‐ to 40‐μm‐long axonal fragment were randomly selected from individual neuron using ImageJ ROI manager plugin. Aβ puncta were manually quantified from each axonal fragment and represented as a number of puncta per 10 μm of axonal length.

### Live imaging

Cultured WT and AP‐2μ KO neurons were either transfected with HA‐BACE1‐eGFP and BACE1‐eGFP alone or co‐transfected with AP‐2μ‐mCherry, AP‐2μ‐mRFP, RFP‐LC3B‐G120, HA‐AP‐2α WT, or HA‐AP‐2α Mut at DIV 7‐8. In case of HA‐BACE1‐eGFP, a single HA tag was introduced after the propeptide cleavage site 23. In control experiments, it was confirmed that BACE1 chimeric protein is able to process APP with efficiencies similar to WT BACE1 23. Transfected neurons were imaged at 37°C at DIV 9–11 in basic buffer (170 mM NaCl, 3.5 mM KCl, 0.4 mM KH_2_PO_4_, 20 mM N‐Tris[hydroxyl‐methyl]‐methyl‐2‐aminoethane‐sulphonic acid (TES), 5 mM NaHCO_3_, 5 mM glucose, 1.2 mM Na_2_SO_4_, 1.2 mM MgCl_2_, 1.3 mM CaCl_2_, pH 7.4) using Zeiss Axiovert 200M microscope (Observer. Z1, Zeiss, USA) equipped with 63×/1.40 oil DIC objective, a pE‐4000 LED light source (CoolLED), and Hamamatsu Orca‐Flash4.O V2 CMOS digital camera. Time‐lapse images of neurons were acquired every second for 60 s using Micro‐Manager software (Micro‐Manager1.4, USA). To quantify the motility of axonal carriers, small axonal fragments were generated using ImageJ ROI manager and kymographs were derived by Kymomaker software 86. The number of moving and stationary carriers was manually quantified from kymographs.

### Rescue experiments using the HA‐BACE1‐LL/AA‐eGFP construct

Cultured control neurons were transfected either with HA‐BACE1‐eGFP or HA‐BACE1‐LL/AA‐eGFP plasmids at DIV 5–6, and experiments were performed at DIV 10–12. To determine the motility of BACE1 vesicles, live‐cell imaging was performed using the protocol for AP‐2μ KO neurons, described above. For the quantification of Aβ puncta, the neurons were fixed with 4% PFA, followed by immunostaining with Aβ antibody (see Table EV1), and the quantification of Aβ puncta per 10 μm of axonal length was using the protocol for AP‐2μ KO neurons, described above. For the Cathepsin D immunostaining, the neurons were fixed and immunostained with Cathepsin D antibody (see Table EV1). To determine the recycling of BACE1, the neurons were pulsed with HA antibody for 20 min at 37°C followed by acid‐stripping, 20 min of chase and surface staining of HA (= recycled fraction) under non‐permeabilizing condition using the protocol, described above for AP‐2μ KO neurons.

### Imaging of BACE1‐mKeima‐Red in cultured neurons

To determine the functionality of mKeima fluorescence probe, BACE1‐mKeima‐Red‐overexpressing neurons were incubated with 75 nM of LysoTracker Blue‐White (Molecular Probes, Cat #L12490) for 15 min. Visualization of BACE1‐mKeima in LysoTracker‐positive organelles was performed in live neurons using confocal microscopy, and the extent of BACE1‐mKeima localization to lysosomes was quantified by ImageJ. To analyze the delivery of BACE1 to lysosomes in AP‐2μ KO condition, WT and KO neurons were co‐transfected with BACE1‐mKeima and eBFP2‐N1 plasmids at DIV 7–8. Neurons were imaged at DIV 11–12 using Leica SP8 confocal microscope (Leica Microsystems), equipped with a 63×/1.32 oil DIC objective and a pulsed excitation white light laser (WLL; < 80‐ps pulse width, 80 MHz repetition rate; NKT Photonics). The temperature of live‐cell imaging chamber was always maintained at 37°C. mKeima was sequentially excited at 438 and 550 nm with the pulsed WLL, and the emission at 620 nm was detected with a hybrid detector. The BFP was excited at 383 nm using an UV lamp and detected at 445 nm. For the quantification, the entire area of the neuron was manually selected using the ImageJ ROI manager, and the mean gray values at 438 nm and 550 nm of excitation were measured in the ROI after the background subtraction. The data were represented as the 550/438 ratio in WT and KO condition.

### Quantitative RT–PCR

RNA isolation from DIV 11‐12 cultured neurons was performed using TRIzol (Thermo Fisher Scientific) 32. Twenty nanogram of total RNA was used for reverse transcription using the High Capacity cDNA Reverse Transcription Kit (Applied Biosystems). The qPCR was performed with qPCR BIO SyGreen Mix Kit (Applied Biosystems) in a 7500 Fast System. mRNA levels of *Bace1* were quantified by normalizing to *Gapdh* 87.

### ELISA

Aβ_1–40_ and Aβ_1–42_ peptides in the media of cultured neurons were analyzed at DIV 11–12 using WAKO kit (# 294‐64701 and # 292‐64501, respectively) following the manufacturer's instructions.

### Immunohistochemistry

Mice were sacrificed by transcardial perfusion either on postnatal day (p) 18–21 or at 13 weeks as previously described 32. Brains were carefully taken out of the skull, postfixed overnight in the same fixative, and placed in a mixture of 20% glycerol and 2% dimethyl sulfoxide in 0.1 M PBS for 24 h for cryoprotection. Frozen horizontal or coronal 40 μm sections were collected in five series and stored at ‐80°C. Immunohistochemistry was performed according to a previously described protocol 32. Images were acquired with Leica SP8 confocal microscopy equipped with 63×/1.32 oil DIC objective and pulse excitation white laser (WLL; 80‐ps pulse width, 80 MHz repetition rate; NKT Photonics). Quantification of mean gray value was performed using ImageJ software. Co‐localization between endogenous BACE1 and RAB7 was determined using Pearson's correlation coefficient. The levels of CTFs were determined by quantifying the integrated density of the immunofluorescence. Co‐localization between PSD95 and Bassoon was determined by Mander's overlap coefficient. For Nissl staining, sections were mounted in 0.2% gelatine solution in 250 mM Tris–HCl and processed as previously described 32. For analysis of cell number, three 40‐μm‐thick consecutive sections containing the entorhinal cortex were selected for each genotype and scaled using Aperio ImageScope v12.3.2.8013. Neurons were counted in a fixed sized rectangle using ImageJ v.1.52a, and the cell density was determined by the normalization of the number of cells to the rectangle area.

### Western blot

#### Analysis of lysates from cultured neurons

The lysates of cultured neurons were prepared by harvesting the neurons at DIV 11‐12 using radioimmunoprecipitation assay buffer (RIPA) containing 50 mM Tris (pH = 8.0), 150 mM NaCl, 1.0% IGEPAL, 0.5% sodium deoxycholate, 0.1% SDS, and 1× protease inhibitor cocktail (Roche). Supernatants were collected after centrifugation at 14,000 *g* for 10 min, and protein concentration was measured by the Bradford assay. Samples were analyzed by SDS–PAGE on 10% Tris‐Glycine gels followed by blotting on the nitrocellulose membrane. The membranes were blocked for 1 h at RT in 5% skim milk in TBS buffer (20 mM Tris pH = 7.6, 150 mM NaCl) containing 0.1% Tween (TBS‐T) and incubated with primary antibodies (Table EV1) overnight at 4°C. Next, the membranes were washed two times (10 min each) with TBS‐T and one time with TBS buffer and incubated with secondary antibody for 1.5 h at RT with 5% skim milk in TBS‐T buffer. Afterward, the membranes were washed three times as above and subsequently developed using ECL‐based autoradiography film system. Analysis was performed using ImageJ.

#### Analysis of lysates from the cortical tissues

Cortices from WT and *Ap2m1* ^lox/lox^ × *Tubulin1α*‐Cre KO mice were dissected at p20‐21 and homogenized using RIPA buffer, and protein concentration was measured by the Bradford assay. The samples were then processed with 2× Tris‐Tricine sample buffer (8% SDS, 24% Glycerol, 100 mM Tris, 100 mM Tricine, 0.2 M DTT, 0.02% Coomassie brilliant blue G250, pH = 8.25) and separated on 9–16% Tris‐Tricine Gel (3 M Tris, 0.3% SDS, pH = 8.45) using cathode (0.1 M Tris, 0.1 M Tricine, 0.1% SDS, pH = 8.25) and anode (0.2 M Tris, pH = 8.9) buffers, where cathode buffer was used in the inner chamber and anode buffer was used in the outer chamber for separation of samples. Following separation, proteins were blotted using semi‐dry transfer on the nitrocellulose membrane. The membranes were then processed as above and probed with corresponding antibodies (Table EV1).

### Co‐immunoprecipitation

Cortices from 8‐week‐old WT mice were dissected and homogenized in RIPA buffer. Proteins were extracted for 45 min on ice, followed by the centrifugation of lysates at 17,000 *g* for 20 min at 4°C. Equivalent amount of rabbit BACE1 antibody or non‐specific rabbit IgG was coupled to Protein G Dynabeads (Invitrogen). Antibody‐coupled Dynabeads were incubated with the supernatant for 1 h at 4°C on the shaker. Following the incubation, Dynabeads were washed three times with RIPA buffer, and proteins were eluted using SDS–PAGE sample buffer and analyzed by Western blotting. For immunoprecipitation of AP‐2μ the cortices were homogenized in Co‐IP buffer containing 50 mM Tris–HCl pH = 7.4, 100 mM NaCl, 1% NP‐40, 2 mM MgCl_2_, and 1× protease inhibitor. Proteins were extracted as above and incubated with mouse AP‐2μ or equivalent amount of mouse non‐specific IgG‐coupled Protein G Dynabeads. Following incubation, proteins were eluted and analyzed by Western blotting using antibodies described in Table EV1.

### Surface biotinylation assay

Surface biotinylation assay was performed on WT‐ and AP‐2μ KO‐cultured neurons at DIV 11–12. Neurons were first incubated with NHS‐Sulfo‐linked biotin (1 mg/ml in PBS; EZ‐linked™‐Sulfo‐NHS‐Biotin, Thermo Fisher Scientific) for 15 min on ice under gentle shaking. Next, the neurons were washed twice with quenching solution (10 mM Glycine in PBS) to remove the excess biotin and one time with ice‐cold PBS. Following the washing steps, the neurons were harvested in RIPA buffer and protein extraction was performed as above. After the centrifugation (15,000 *g* for 20 min at 4°C), supernatants were collected and protein concentrations were measured. A portion of the supernatant was collected and used as input. To precipitate the biotinylated proteins, 60–80 μg of the supernatant was incubated with Neutravidin agarose beads (Thermo Fisher Scientific) for 1 h at room temperature. Following the incubation, the beads were washed in RIPA buffer and proteins were eluted from the streptavidin beads by boiling in reducing sample buffer and then resolved by SDS–PAGE and immunoblotted using corresponding antibodies (BACE1, GAPDH, and AP‐2α, Table EV1).

### HEK cells

HEK293T cells were maintained in DMEM (GIBCO), containing 10% FCS, penicillin (255 Units/ml), and streptomycin (255 μg/ml). Twenty‐four hours after seeding, the cells were transfected with mouse FLAG‐BACE1 plasmid and co‐transfected with either BACE1 shRNA or scrambled shRNA using the Lipofectamine 3000 (Invitrogen) (Tables EV2 and EV3). Ninety hours post‐transfection, the cells were harvested and lysed in RIPA buffer. Extracted proteins were loaded onto a 10% SDS–PAGE gel, transferred onto the PVDF membranes, and subsequently probed with corresponding primary and HRP‐conjugated secondary antibodies (Table EV1). Blots were developed using the ECL‐based autoradiography film system and analyzed using ImageJ (NIH).

### Transferrin uptake assay

HEK293T cells were transfected either with AP‐2μ 88 or scrambled siRNA 89 (see Table EV3) using Lipofectamine RNAiMax Reagent (Invitrogen) on day 1 and co‐transfected with mCherry or AP‐2μ‐mCherry on day 2, followed by transfection with siRNA on day 3. Transferrin uptake was performed at day 5. Briefly, cells were incubated with starvation medium (DMEM containing 20 mM HEPES and 5 mg/ml BSA) for 30 min at 37°C. After the starvation, the cells were incubated with 50 μg/ml Transferrin‐Alexa‐488 (Invitrogen) for 10 min in the same media at 37°C in CO_2_ incubator. Cells were then washed three times with ice‐cold PBS, and fixed and stained with mCherry antibody (Table EV1).

### iPSC‐derived neurons

#### iPSC lines

We used previously published iPSC lines derived from AD patients, as well as control individuals without dementia for the current study 90, 91, 92, 93 (Table EV4). The iPSCs were maintained on Matrigel‐coated (Corning) plates in StemMACS culture medium (Miltenyi Biotec). Medium was changed every day and cells passaged every 5–6 days using PBS lacking calcium and magnesium (Life Technologies).

#### Neural differentiation of the iPSC lines

For the neuronal differentiation, the embryoid body‐based protocol was employed 94. Briefly, on day 0, cells were harvested and re‐cultivated in suspension in neural induction medium (NIM) (DMEM/F‐12 (Thermo Scientific), 1% NEAA (Lonza), 1% N2 supplement (Thermo Scientific), 2 μg/ml of Heparin (Sigma‐Aldrich), and 1% P/S) supplemented with 1 μM purmorphamine (Tocris Bioscience). At day 5, formed aggregates were harvested and re‐plated as adherent cells in the same medium with purmorphamine. From days 10 to 18, primitive neuro‐epithelia structures formed. Neural rosettes were selected with STEMdiff Neural Rosette Selection Reagent (Stem Cell Technologies) and re‐cultured using NIM supplemented with B27 lacking retinoic acid (Thermo Scientific). After 10 days, the cells maintained as aggregates (neurospheres) were dissociated into single cells using accutase (Invitrogen), which were re‐plated using neural differentiation medium (NDM) (Neurobasal 1% NEAA, 1% N2 supplement, 1% P/S supplemented with 1 μM of cAMP (Sigma‐Aldrich), and 10 ng/mL of BDNF, GDNF, and IGF‐1 (all Immuno Tools)) on Matrigel (Corning). The iPSCs‐derived neurons were then cultivated for approximately 2 months and harvested in RIPA buffer, and protein extraction was performed as described above. Ten microgram of supernatants was loaded on to 10% gel and analyzed by immunoblotting using antibodies against AP‐2α. The levels of AP‐2α were normalized against GAPDH (Table EV1).

### Novel object recognition test (NOR)

The novel object recognition was performed on 19‐ to 20‐day‐old WT and AP‐2μ KO animals in a white painted square box (50 × 50 × 40 cm) illuminated with > 20lux. Objects used were a blue‐colored cool pack, a diverse colored cube, and a flask filled with sand and stones. Objects differed in height, width, color, and shape and previous experiments demonstrated no spontaneous preference for none of the objects. Mice were monitored by a digital camera placed above the arena and connected to a video tracking system (EthoVision^®^XT, Noldus). Animals were first handled for 4 min on three successive days prior to the habituation phase. Habituation to the environment was performed on the fourth day, exposing the mice to the open field for 10 min. On the fifth day, the training session was performed exposing the mice for 12 min to two copies of the same object (blue‐colored cool pack), placed in the back left and right corners of the box. Animals were placed individually at the mid‐point of the wall opposite to the sample objects with the nose pointing away from the objects. The positioning of the animals and objects was similar during the training and test sessions. One hour and a half after the training session, the first test session was performed. Mice were exposed for 10 min to one of the objects used in the training session and to a novel object (diverse colored cube). Twenty‐four hours after the training session, the second test was performed by exposing the mice for 10 min to one of the objects used in the training session and a second novel object (flask). The animals were taken into the behavioral room 1 h before the habituation, training, and test sessions to avoid the stress. Before and after the testing, the box was always cleaned with water and soap first, then with water only and finally with 70% ethanol, followed by waiting period of at least 10 min. Males were always tested first.

### Statistical analysis

Statistically significant estimates were obtained from total number of neurons or mice collected from minimum three independent experiments (N) (exceptions are control experiments Figs EV1I and EV3B and F). The statistical significance between two groups for all normally distributed values was evaluated with a two‐tailed unpaired Student's *t*‐test or one‐sample *t*‐test for normalized data. The statistical significance between more than two groups for all normally distributed values was evaluated using two‐way ANOVA (Tukey's post hoc test was used to determine the statistical significance between the groups). Significant differences were accepted at *P* < 0.05. All data shown represent the mean ± SEM from cells or axonal fragments acquired from N independent experiments. Power analysis was used to calculate the sample size (alpha 0.05). Non‐normally distributed data (as in Fig EV4N and O) were transformed using square root transformation and analyzed using paired two‐tailed *t*‐test.

## Author contributions

SB and NLK contributed to the conception of the study and experimental design. SB, SC‐P, ECB, AN‐H, JR, EB, CW, NE, SM, and NLK performed the experiments, data analysis, and participated in the acquisition of data. JA provided reagents. SB and NLK prepared the manuscript. All the authors critically revised the manuscript and provided help with the interpretation of data. The final manuscript was read and approved by all authors.

## Conflict of interest

The authors declare that they have no conflict of interest.

## Supporting information



Expanded View Figures PDF

Table EV1

Table EV2

Table EV3

Table EV4

Movie EV1

Movie EV2

Source Data for Expanded View

Review Process File

Source Data for Figure 1

Source Data for Figure 2

Source Data for Figure 3

Source Data for Figure 4

Source Data for Figure 5
