# Supplementary material for: AP‐2 reduces amyloidogenesis by promoting BACE1 trafficking and degradation in neurons
Source: EMBO Rep. 2020 Apr 23;21(6):e47954. doi: 10.15252/embr.201947954 (PMC7271323; doi:10.15252/embr.201947954)
Supplement: Supplementary file 1 — Expanded View Figures PDF [file EMBR-21-e47954-s001.pdf]

Expanded View Figures

Figure EV1. AP-2 regulates the recycling of BACE1 in the brain.

A BACE1 is co-immunoprecipitated (Co-IP) by the AP-2μ-specific antibody (IP) from the mouse brain cortex (high exposure was used to show the input).
B AP-2α is co-immunoprecipitated (Co-IP) by the BACE1-specific antibody (IP) from the mouse brain cortex (high exposure was used to show the input).
C Representative fluorescence images of neurons expressing the HA-BACE1-eGFP and immunostained for endogenous BACE1. Scale bars: 20 μm (left panel), 5 μm (zoomed images).
D Representative fluorescence images of neurons expressing the HA-BACE1-eGFP or non-transfected neurons immunostained for endogenous BACE1. Scale bars: 20 μm.
E Schematic illustration of the assay used in Fig 1A.
F Acid-stripping control for the removal of the membrane-bound HA antibody in AP-2μ KO neurons, overexpressing the HA-BACE1-eGFP. Scale bar: 10 μm.
G Overview images of HA-BACE1-eGFP-expressing neurons shown in Fig 1C. Scale bar: 15 μm.
H Overview images of HA-BACE1-eGFP-expressing neurons shown in Fig 1E. Scale bar: 15 μm.
I Recycled-to-total BACE1 ratio in WT neurons overexpressing either eGFP or eGFP-RAB4. Twenty-eight eGFP-expressing neurons and 22 eGFP-RAB4-expressing neurons, N = 2 biological replicates.
J, K Overview images of HA-BACE1-mCherry-expressing neurons, additionally co-expressing either GFP-RAB4 or GFP-RAB4S22N, shown in Fig 1G. Magnified axonal fragments are shown in (K). Scale bars: 10 μm.
L Recycled levels of BACE1 after 20 min of HA antibody chase in HA-BACE1-mCherry-transfected WT and KO neurons, co-expressing either eGFP or eGFP-RAB11-S25N, calculated as the HA(recycled)/mCherry(total) signal intensity ratio (WT^{GFP}: 0.12 ± 0.02, KO^{GFP}: 0.23 ± 0.04, $WT^{GFP\text{-}RAB11\text{-}S25N}$: 0.08 ± 0.02, $KO^{GFP\text{-}RAB11\text{-}S25N}$: 0.13 ± 0.02, pWT^{GFP} versus pKO^{GFP} = 0.024, pWT^{GFP} versus $pWT^{GFP\text{-}RAB11\text{-}S25N}$ = 0.840, pKO^{GFP} versus $pKO^{GFP\text{-}RAB11\text{-}S25N}$ = 0.047, $pWT^{GFP\text{-}RAB11\text{-}S25N}$ versus $pKO^{GFP\text{-}RAB11\text{-}S25N}$ = 0.587, 20–24 neurons per condition, N = 3 biological replicates).
M, N Overview images of HA-BACE1-mCherry-expressing neurons, additionally co-expressing either GFP-RAB4 or GFP-RAB4S22N, shown in Fig 1J. Magnified axonal fragments are shown in (N). Scale bars: 10 μm.
O Levels of internalized BACE1 are increased in AP-2μ KO neurons overexpressing the RAB11-S25N mutant (WT^{GFP}: 0.46 ± 0.05, KO^{GFP}: 0.26 ± 0.02, $WT^{GFP\text{-}RAB11A\text{-}S25N}$: 0.49 ± 0.03, $KO^{GFP\text{-}RAB11A\text{-}S25N}$: 0.38 ± 0.03, pWT^{GFP} versus pKO^{GFP} = 0.000, pWT^{GFP} versus $pWT^{GFP\text{-}RAB11\text{-}S25N}$ = 0.919; pKO^{GFP} versus $pKO^{GFP\text{-}RAB11\text{-}S25N}$ = 0.044, $pWT^{GFP\text{-}RAB11\text{-}S25N}$ versus $pKO^{GFP\text{-}RAB11\text{-}S25N}$ = 0.105, 40-42 neurons for each condition, N = 5 biological replicates).
P Levels of BACE1 are significantly increased in AP-2μ KO-cultured neuronal lysates compared to the WT set to 100% (KO: 165.42 ± 25.3, P = 0.040, also see Fig 1L). N = 4 biological replicates.
Q, R Loss of AP-2μ does not impair the trafficking of BACE1 toward early endosomes, marked by overexpression of RAB5-Q79L in WT and AP-2μ KO neurons. Line plot analysis in (R) indicates BACE1 distribution in early endosomes marked by dotted lines in (Q). Scale bar: 5 μm.
S Overview images of neurons shown in Fig 1O. Scale bar, 50 μm.
T, U Overview images of WT and AP-2μ KO neurons immunostained for surface and total GABARB3. Ratio of surface to total GABARB3 is increased in KO neurons compared to WT (WT: 0.24 ± 0.03, KO: 0.34 ± 0.03, P = 0.024, 32 WT and 38 KO neurons, N = 3 biological replicates). Scale bar: 20 μm.

Data information: Rectangles in Appendix Fig S1G, H, J and M indicate regions magnified in Fig 1. All graphs show mean ± SEM; statistical analysis was performed by unpaired two-tailed Student's t-test in (I, U), two-way ANOVA in (L, O), and one-sample Student's t-test in (P). n.s.—non-significant. * indicates $P \leq 0.05$; ** indicates $P \leq 0.01$; *** indicates $P \leq 0.001$.

© 2020 The Authors

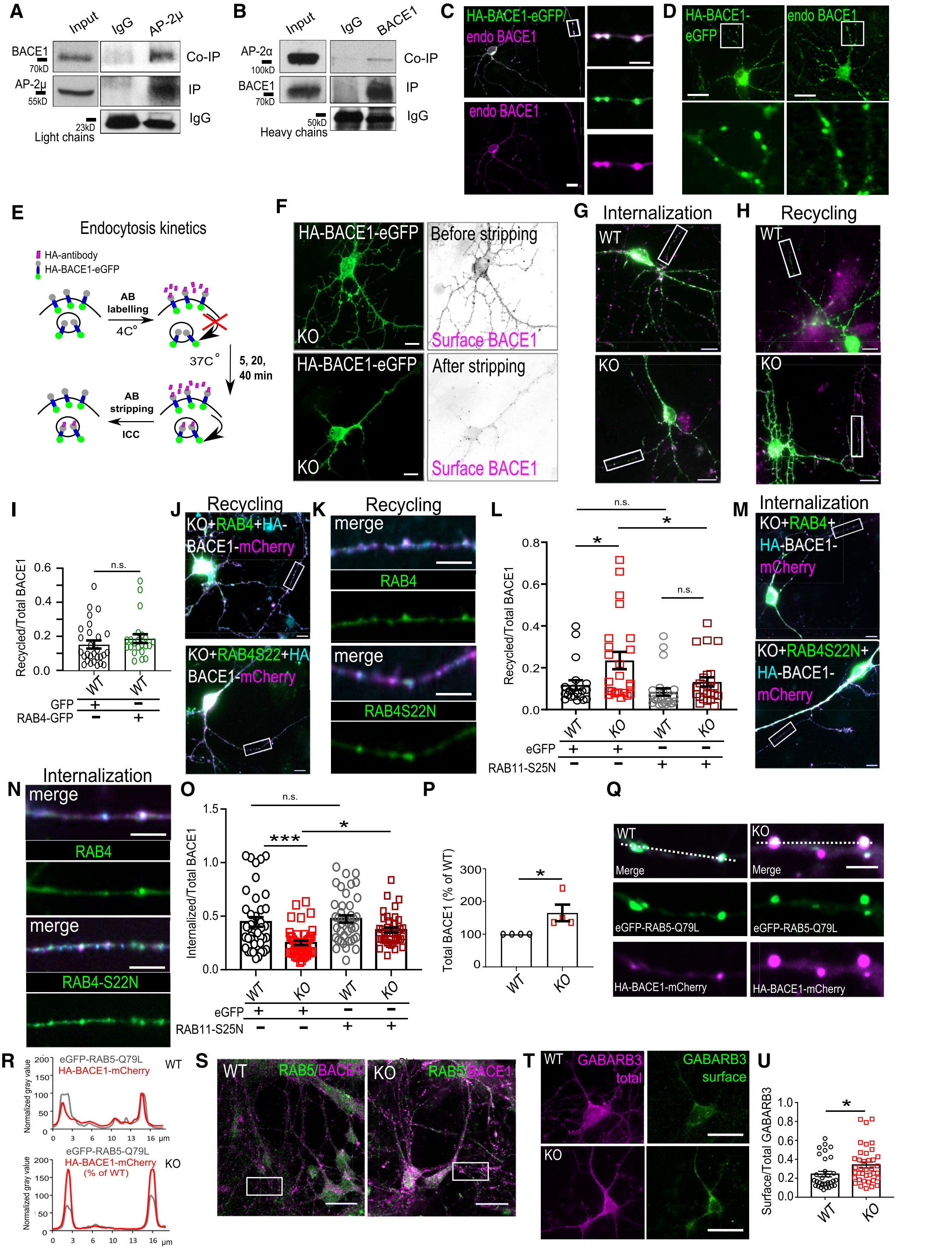
A
Input
IgG
AP-2μ
BACE1
70kD
AP-2μ
55kD
23kD
Light chains
Co-IP
IP
IgG
B
Input
IgG
BACE1
AP-2α
100kD
BACE1
70kD
50kD
Heavy chains
Co-IP
IP
IgG
C
HA-BACE1-eGFP/
endo BACE1
endo BACE1
D
HA-BACE1-
eGFP
endo BACE1
E
Endocytosis kinetics
HA-antibody
HA-BACE1-eGFP
AB
labelling
4C°
37C°
5, 20,
40 min
AB
stripping
ICC
F
HA-BACE1-eGFP
Before stripping
KO
Surface BACE1
HA-BACE1-eGFP
After stripping
KO
Surface BACE1
G
Internalization
WT
KO
H
Recycling
WT
KO
I
Recycled/Total BACE1
n.s.
WT
WT
GFP
RAB4-GFP
J
Recycling
KO+RAB4+HA-
BACE1-mCherry
KO+RAB4S22+HA
BACE1-mCherry
K
Recycling
merge
RAB4
merge
RAB4S22N
L
n.s.
Recycled/Total BACE1
eGFP
RAB11-S25N
WT
KO
WT
KO
M
Internalization
KO+RAB4+
HA-BACE1-
mCherry
KO+RAB4S22N+
HA-BACE1-
mCherry
N
Internalization
merge
RAB4
merge
RAB4-S22N
O
n.s.
Internalized/Total BACE1
eGFP
RAB11-S25N
WT
KO
WT
KO
P
Total BACE1 (% of WT)
WT
KO
Q
WT
KO
Merge
Merge
eGFP-RAB5-Q79L
eGFP-RAB5-Q79L
HA-BACE1-mCherry
HA-BACE1-mCherry
R
eGFP-RAB5-Q79L
HA-BACE1-mCherry
WT
Normalized gray value
eGFP-RAB5-Q79L
HA-BACE1-mCherry
(% of WT)
KO
μm
S
WT
RAB5/BACE1
KO
RAB5/BACE1
T
WT
GABARB3
total
GABARB3
surface
KO
U
Surface/Total GABARB3
WT
KO

Figure EV1.

Figure EV2. AP-2μ regulates lysosomal BACE1 levels.

A Significantly reduced AP-2α levels in the cortex of AP-2μ KO mice. Protein levels in the KO were normalized to the WT set to 100% (KO: 31.49 ± 5.54, $P < 0.000$, $N = 7$ biological replicates).

B, C Significantly reduced AP-2μ levels in the cortex of AP-2μ KO mice. Protein levels in the KO were normalized to the WT set to 100% (KO: 20.05 ± 5.39%, $P = 0.000$, $N = 4$ biological replicates).

D, E Significantly reduced levels of AP-2μ in AP-2μ KO-cultured neurons. Protein levels in the KO were normalized to the WT set to 100% (KO: 8.87 ± 3.17%, $P = 0.000$, $N = 3$ biological replicates).

F, G Significantly increased BACE1 levels in AP-2μ KO-cultured neurons. Protein levels in the KO were normalized to the WT set to 100% (KO: 180.51 ± 25.99%, $P = 0.037$, $N = 4$ biological replicates).

H *Bace1* mRNA levels measured by qPCR are not significantly altered in AP-2μ KO neurons (KO/WT^{Bace1}: 1.00 ± 0.03, KO/WT^{Gapdh}: 0.99 ± 0.02, $P = 0.921$, $N = 3$ biological replicates). mRNA levels in the KO were normalized to the WT set to 1.

I Overview images of neurites represented in Fig 2G. Scale bar, 20 μm.

J Overview images of neurites represented in Fig 2I. Scale bar, 20 μm.

K, L Horizontal sections from WT and AP-2μ KO entorhinal cortex immunostained with either LAMP2a (K) or MAP2 (L) antibodies. Scale bars: 20 μm

M BACE1 levels are unaltered in lysates from cultured WT and AP-2μ KO neurons, either treated with 5 μM cycloheximide alone or co-treated with 80 nM bafilomycin A for 12 h.

N Representative confocal images of BACE1-mKeima-Red-expressing neurons incubated with LysoTracker Blue-White for 15 min to label the lysosomes. Scale bar: 10 μm, 1 μm for zoomed images.

O Percentage of BACE1-mKeima-Red puncta localized to lysosomes (94.61 ± 4.12), 17 cells from $N = 2$ biological replicates.

P Representative confocal images of BACE1-mKeima-Red-expressing WT and AP-2μ KO neurons. In the KO, the majority of BACE1-mKeima organelles at neutral pH was confined to axons located distally from the soma (arrows). Scale bars: 20 μm.

Data information: All graphs show mean ± SEM; statistical analysis was performed by unpaired one-sample Student's *t*-test in (A, C, E, G, H). n.s.—non-significant. * indicates $P \leq 0.05$; ** indicates $P \leq 0.01$; *** indicates $P \leq 0.001$.

 © 2020 The Authors

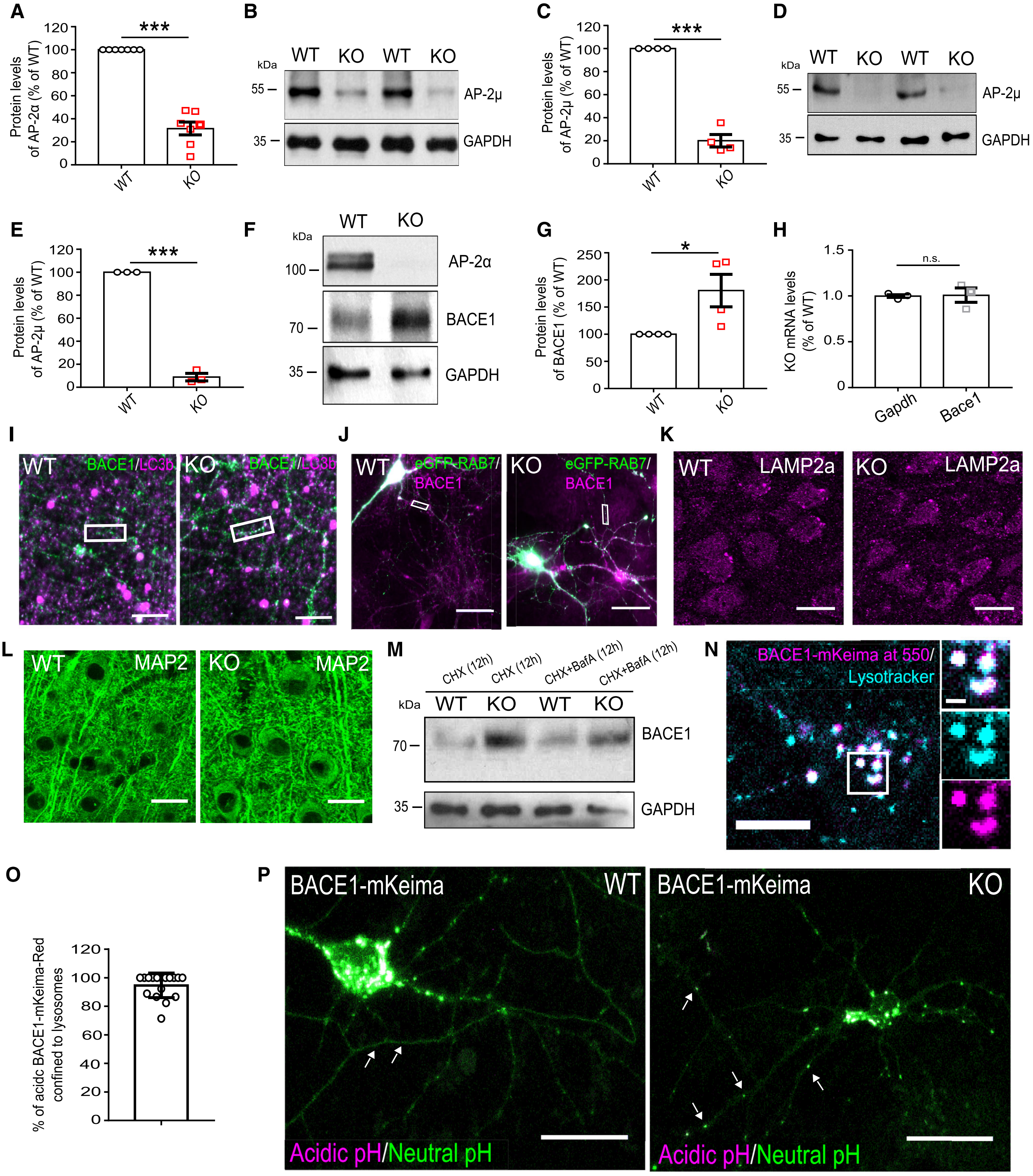
A
Protein levels of AP-2α (% of WT)
***
WT
KO
B
kDa
WT KO WT KO
55
35
AP-2μ
GAPDH
C
Protein levels of AP-2μ (% of WT)
***
D
E
Protein levels of AP-2μ (% of WT)
F
AP-2α
BACE1
G
Protein levels of BACE1 (% of WT)
*
H
KO mRNA levels (% of WT)
n.s.
Gapdh
Bace1
I
BACE1/LC3b
J
eGFP-RAB7/ BACE1
K
LAMP2a
L
MAP2
M
CHX (12h)
CHX+BafA (12h)
N
BACE1-mKeima at 550/ Lysotracker
O
% of acidc BACE1-mKeima-Red confined to lysosomes
P
BACE1-mKeima
Acidic pH/Neutral pH

Figure EV2.

Figure EV3. Functionality of AP-2μ-mCherry and HA-BACE1-eGFP constructs.

- A, B Overexpressed AP-2μ-mCherry restores clathrin-mediated endocytosis of transferrin in HEK cells depleted of endogenous AP-2μ ($mCherry^{scr}$: 0.19 ± 0.02, $mCherry^{AP-2\mu\text{-}siRNA}$: 0.09 ± 0.01, AP-2μ-$mCherry^{scr}$: 0.23 ± 0.01, AP-2μ-$mCherry^{AP-2\mu\text{-}siRNA}$: 0.25 ± 0.01, $p^{mCherry\text{-}scr}$ versus $_{p}{}^{mCherry\text{-}AP-2\mu\text{-}siRNA}$ < 0.000; $p^{mCherry\text{-}scr}$ versus $p^{AP-2\mu\text{-}mCherry\text{-}scr}$ = 0.289, $p^{mCherry\text{-}AP-2\mu\text{-}siRNA}$ versus $p^{AP-2\mu\text{-}mCherry\text{-}AP-2\mu\text{-}siRNA}$ < 0.000, $p^{AP-2\mu\text{-}mCherry\text{-}scr\text{-}siRNA}$ versus $p^{AP-2\mu\text{-}mCherry\text{-}AP-2\mu\text{-}siRNA}$ = 0.690, 26 cells for each condition, *N* = 1 biological replicates). Scale bars: 10 μm.
- C, D Large portion (81.8 ± 3.37%) of overexpressed AP-2μ-mCherry associates with endogenous AP-2α, 20 axons from 10 neurons. Scale bar: 2 μm.
- E, F $A\beta_{1-42}$ levels are upregulated in neurons overexpressing the HA-BACE1-eGFP, comparing to cells expressing the eGFP only (GFP: 0.24 ± 0.8, BACE1: 0.79 ± 0.16, *P* = 0.006). Eleven eGFP- and 12 HA-BACE1-eGFP-expressing neurons, *N* = 1 biological replicates. Scale bars: 20 μm.
- G Histogram representation of BACE1 retrograde velocity in control neurons overexpressing either the HA-BACE1-eGFP or the BACE1-eGFP (axonal fragments from 28 to 31 neurons, *N* = 3 biological replicates).
- H Percentage of BACE1 puncta colocalizing with AP-2μ in control neurons (74.22 ± 2.09%), 48 neurons, *N* = 4 biological replicates.
- I Percentage of AP-2μ puncta colocalizing with BACE1 in control neurons (83.35 ± 2.56%), 46 neurons, *N* = 4.
- J, K Co-localization of endogenous BACE1 and AP-2μ, measured using Pearson's co-localization coefficient (0.88 ± 0.01, 36 neurons, *N* = 3 biological replicates). Scale bars, 20 μm (left panel), 5 μm (zoomed images).
- L Percentage of mobile BACE1 carriers positive for AP-2μ in control neurons, normalized to total double-labeled puncta set to 100% (20.12 ± 1.95%, 48 neurons, *N* = 4 biological replicates).
- M Representative fluorescence images and corresponding kymographs from time-lapse videos of WT and AP-2μ KO neurons transfected with BACE1-eGFP. Scale bars: 2 μm top panels, x = 2 μm, y = 5s bottom panels.
- N Retrograde velocity of BACE1-eGFP carriers is significantly reduced in AP-2μ KO neurons (WT^{Retro}: 0.37 ± 0.03, KO^{Retro} = 0.25 ± 0.03, *P* = 0.002, 29 WT and 30 KO neurons, *N* = 3 biological replicates). In these experiments, live-cell imaging was performed 24 h post-transfection to exclude the overexpression artifact.
- O Retrograde mobility of autophagic BACE1 in WT and AP-2μ KO neurons (WT: 18.74 ± 2.92%, KO: 6.60 ± 1.6%, *P* = 0.000), 36 WT and 36 KO neurons, *N* = 4 biological replicates.
- P, Q Significantly decreased retrograde mobility of BACE1 in HA-BACE1-eGFP-expressing ATG5 KO neurons comparing to the WT (WT: 48.63 ± 3.94%, KO^{ATG5}: 34.96 ± 3.22%, *P* = 0.008, 30 WT and 35 KO neurons, *N* = 3 biological replicates). Scale bars: 2 μm top panels, x = 2 μm, y = 5 s bottom panels.
- R, S Increased recycling of BACE1-LL/AA when compared to WT BACE1 (WT: 0.05 ± 0.00, LL/AA: 0.09 ± 0.01, *P* = 0.003, 25–26 neurons per condition, *N* = 4 biological replicates). Scale bar, 5 μm.
- T Representative images of neurons expressing either HA-BACE1-GFP or HA-BACE1-LL/AA-GFP and immunostained for Cathepsin D (CTSD). Scale bar 5 μm.
- U Significantly decreased percentage of HA-BACE1-LL/AA puncta found in lysosomes compared to WT BACE1 (WT: 34.99 ± 5.22%, KO: 19.98 ± 3.49%, *P* = 0.019, 22-24 neurons per group, *N* = 3 biological replicates.

Data information: All graphs show mean ± SEM; statistical analysis was performed by unpaired two-tailed Student's *t*-test in (F, N, O, Q, S, U) and two-way ANOVA in (B). n.s.—non-significant.* indicates $P \leq 0.05$; ** indicates $P \leq 0.01$; *** indicates $P \leq 0.001$.

 © 2020 The Authors

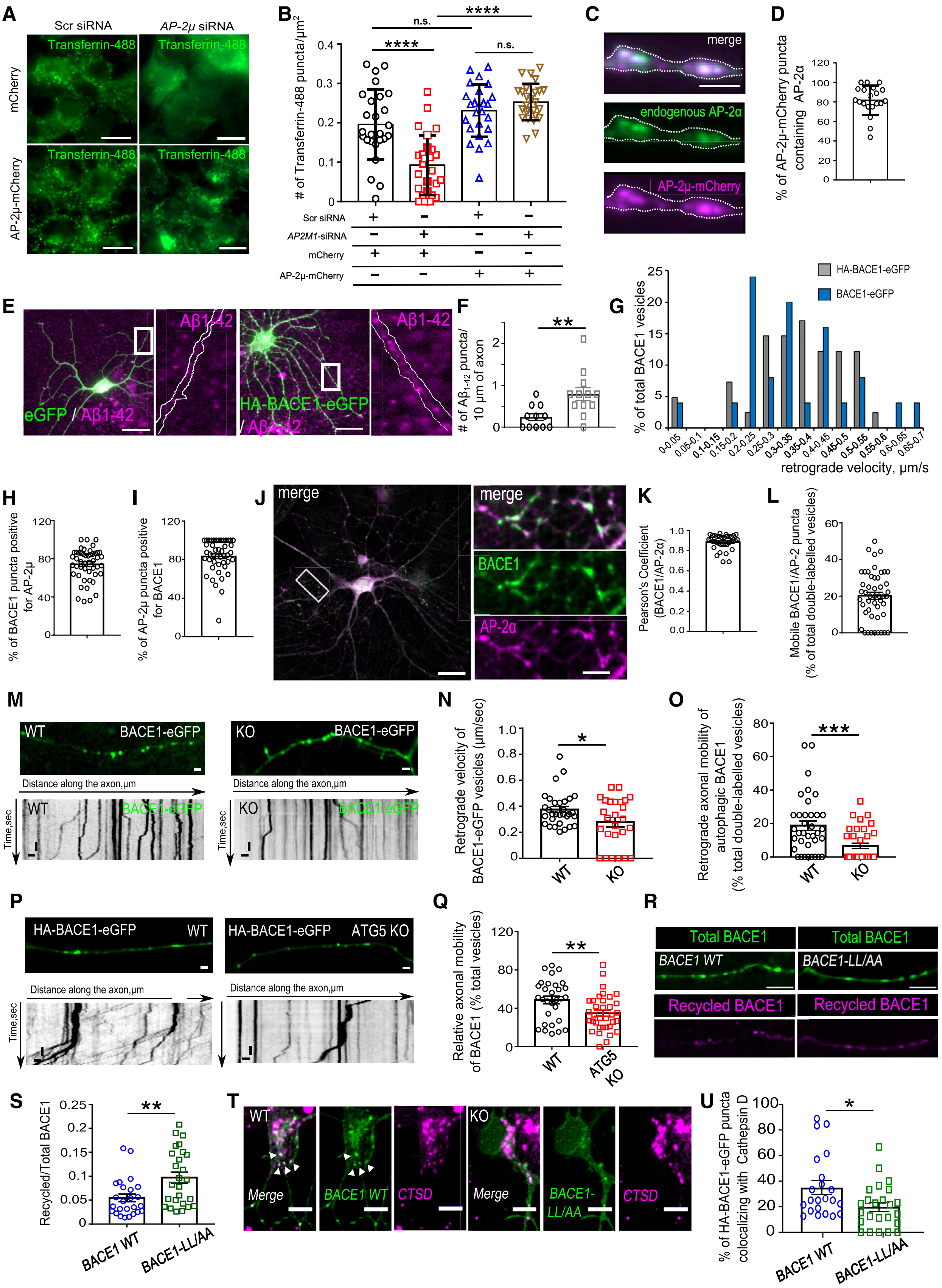
A
Scr siRNA
AP-2μ siRNA
mCherry
AP-2μ-mCherry
Transferrin-488
B
# of Transferrin-488 puncta/μm2
n.s.
****
Scr siRNA
AP2M1-siRNA
mCherry
AP-2μ-mCherry
C
merge
endogenous AP-2α
AP-2μ-mCherry
D
% of AP-2μ-mCherry puncta containing AP-2α
E
eGFP / Aβ1-42
HA-BACE1-eGFP / Aβ1-42
Aβ1-42
F
# of Aβ1-42 puncta/ 10 μm of axon
**
G
% of total BACE1 vesicles
HA-BACE1-eGFP
BACE1-eGFP
retrograde velocity, μm/s
H
% of BACE1 puncta positive for AP-2μ
I
% of AP-2μ puncta positive for BACE1
J
merge
BACE1
AP-2α
K
Pearson's Coefficient (BACE1/AP-2α)
L
Mobile BACE1/AP-2 puncta (% of total double-labelled vesicles)
M
WT
KO
BACE1-eGFP
Distance along the axon,μm
Time,sec
N
Retrograde velocity of BACE1-eGFP vesicles (μm/sec)
*
O
Retrograde axonal mobility of autophagic BACE1 (% total double-labelled vesicles)
***
P
HA-BACE1-eGFP
WT
ATG5 KO
Distance along the axon,μm
Time,sec
Q
Relative axonal mobility of BACE1 (% total vesicles)
**
ATG5 KO
R
Total BACE1
BACE1 WT
BACE1-LL/AA
Recycled BACE1
S
Recycled/Total BACE1
**
BACE1 WT
BACE1-LL/AA
T
WT
KO
Merge
BACE1 WT
CTSD
BACE1-LL/AA
U
% of HA-BACE1-eGFP puncta colocalizing with Cathepsin D
*

Figure EV3.

Figure EV4. Regulation of APP processing by the AP-2μ in neurons.

- A Schematic illustration of BACE1 activity monitored using the APP Lys612Val mutant (mCherry-APP-P1-eGFP).
- B Representative fluorescence images of mCherry-APP-P1-eGFP-expressing WT and AP-2μ KO neurons, immunostained for mCherry and GFP. Scale bar, 10 μm.
- C Representative fluorescence images of HA-BACE1-eGFP-expressing WT and AP-2μ KO neurons, immunostained for APP. Scale bars: 5 μm (upper panels), 2 μm (lower panels).
- D Percentage of BACE1 puncta colocalizing with APP in WT and AP-2μ KO neurons (WT: 17.27 ± 1.66%, KO: 29.19 ± 3.57%, P = 0.003, 40 WT and 40 KO neurons, N = 4 biological replicates).
- E Percentage of APP puncta colocalizing with BACE1 in WT and AP-2μ KO neurons (WT: 22.78 ± 2.52%, KO: 31.78 ± 3.18%, P = 0.030, 36 WT and 30 KO neurons, N = 4 biological replicates).
- F WT and AP-2μ KO entorhinal cortex of mice at p21 immunostained for Reelin. Scale bars: 50 μm.
- G, H Levels of L1-CTFβ are significantly increased in AP-2μ KO cortex compared to the WT set to 100% (KO^{C99}: 359.87 ± 95.77%, P = 0.015, N = 4 biological replicates).
- I, J The mCherry (surface APP)/eGFP (total APP) signal intensity ratio is significantly increased in AP-2μ KO condition compared to the WT (WT: 1.52 ± 0.19, KO: 2.43 ± 0.36, P = 0.028, 30 WT and 29 KO neurons, N = 3 biological replicates). Scale bars: 10 μm.
- K Unaltered levels of Nicastrin in cortical lysates from AP-2μ KO mice. Protein levels in KO condition were normalized to the WT set to 100% (KO: 105.03 ± 9.02%, P = 0.300, N = 6 biological replicates).
- L Representative fluorescence images of axons from RFP-LC3B-expressing WT and AP-2μ KO neurons immunostained for $A\beta_{1-42}$. Scale bar: 4 μm. Arrowheads indicate localization of $A\beta_{1-42}$ puncta to LC3B-positive autophagosomes.
- M Autophagosomal levels of $A\beta_{1-42}$ are increased in AP-2μ KO neurons (WT: 1299.58 ± 166.36, KO: 2002.6 ± 185.72, P = 0.006, 41 WT and 41 KO neurons, N = 3 biological replicates). Scale bar: 5 μm.
- N, O Levels of secreted $A\beta_{1-40}$ (N) and $A\beta_{1-42}$ (O) are significantly decreased in the media of AP-2μ KO neurons compared to the WT ($WT^{A\beta1-40}$: 7.85 ± 1.33, $KO^{A\beta1-40}$: 5.35 ± 0.80, P = 0.011, N = 6 biological replicates; $WT^{A\beta1-42}$: 8.69 ± 0.45, $KO^{A\beta1-42}$: 7.26 ± 0.29, P = 0.037, N = 6 biological replicates). Values represent pmol/l raw data, which were square-root-transformed due to the lack of normality.
- P–R Levels of BACE1 are increased in control neurons overexpressing the HA-AP2Mut compared to HA-AP2α WT-expressing cells (WT: 0.22 ± 0.03, Mut: 0.44 ± 0.04, P < 0.000, 25 WT and 28 Mut neurons, N = 3 biological replicates). Rectangles in (P) indicate the area magnified in (Q). Scale bars, 10 μm in (P), 2.5 μm in (Q).
- S, T Analysis of BACE1 KD efficiency in HEK cells, expressing the mouse FLAG-BACE1. *Bace1* shRNA significantly reduces BACE1 levels compared to scr controls set to 100% ($Bace1^{shRNA}$: 7.86 ± 1.78%, P < 0.000, N = 4 biological replicates).
- U, V Number of $A\beta_{1-42}$ puncta is reduced in AP-2μ KO neurons overexpressing the AP-2μ-mRFP, when compared to KO neurons expressing the mCherry (WT^{mChery}: 0.49 ± 0.07, $KO^{mCherry}$: 0.79 ± 0.10, $WT^{AP-2\mu\text{-}mRFP}$: 0.54 ± 0.07, $KO^{AP-2\mu\text{-}mRFP}$: 0.46 ± 0.06, $pWT^{mCherry}$ versus $pKO^{mCherry}$ = 0.026, $pWT^{mCherry}$ versus $pWT^{AP-2\mu\text{-}mRFP}$ = 0.963, $pKO^{mCherry}$ versus $pKO^{AP-2\mu\text{-}mRFP}$ = 0.008, $pWT^{AP-2\mu\text{-}mRFP}$ versus $pKO^{AP-2\mu\text{-}mRFP}$ = 0.840, 38-40 neurons per condition, N = 4 biological replicates). Scale bar: 2 μm.

Data information: All graphs show mean ± SEM; statistical analysis was performed by unpaired two-tailed Student's *t*-test in (D, E, J, M, R), two-way ANOVA in (V), and one-sample Student's *t*-test in (H, K, T). Non-normally distributed data (N,O) were transformed using square root transformation and analyzed using paired two-tailed Student's *t*-test. n.s.—non-significant. * indicates $P \leq 0.05$; ** indicates $P \leq 0.01$; *** indicates $P \leq 0.001$.

© 2020 The Authors

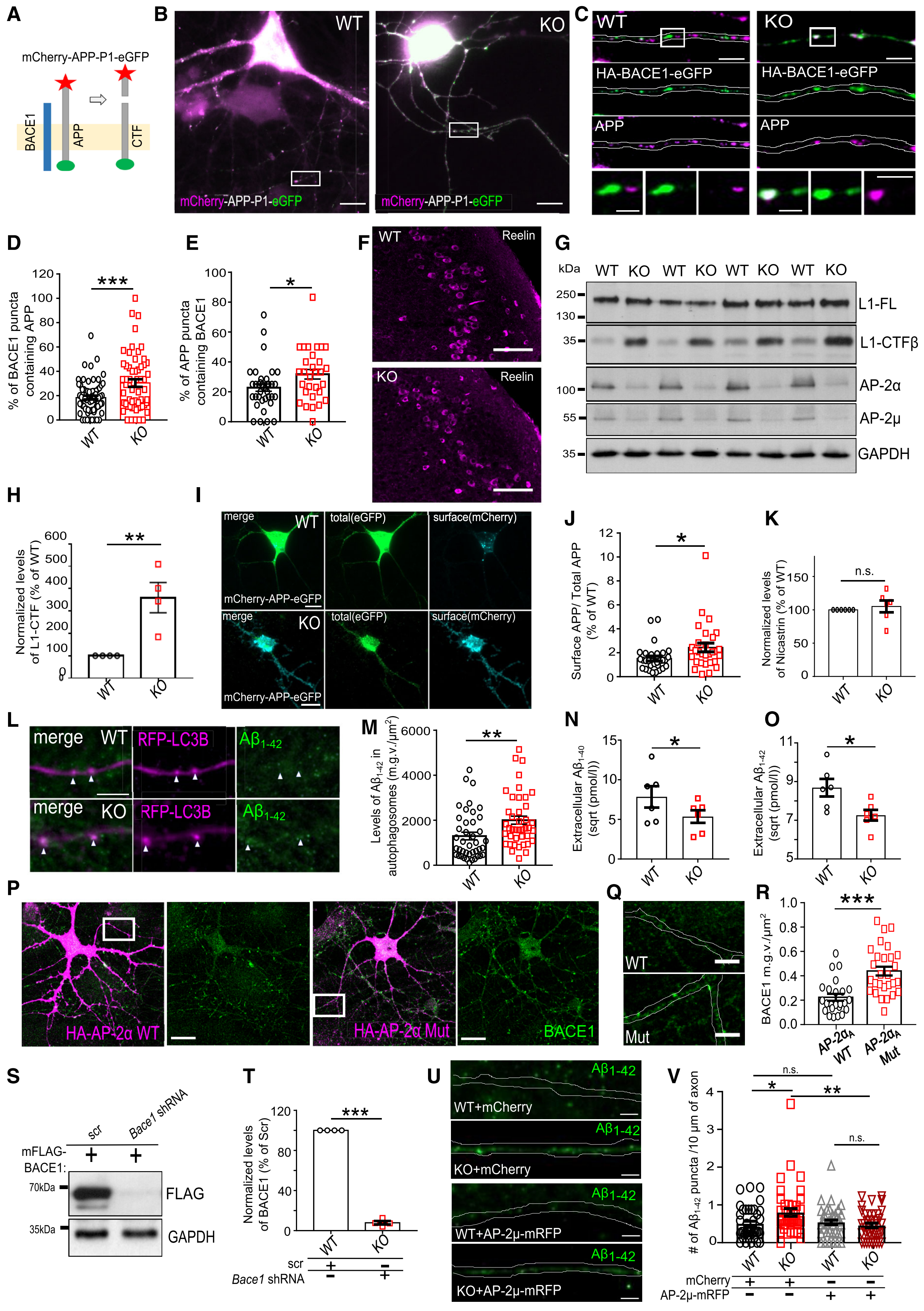
A
mCherry-APP-P1-eGFP
BACE1
APP
CTF
B
WT
KO
mCherry-APP-P1-eGFP
C
HA-BACE1-eGFP
APP
D
% of BACE1 puncta containing APP
E
% of APP puncta containing BACE1
F
Reelin
G
kDa
L1-FL
L1-CTFβ
AP-2α
AP-2μ
GAPDH
H
Normalized levels of L1-CTF (% of WT)
I
merge
total(eGFP)
surface(mCherry)
mCherry-APP-eGFP
J
Surface APP/ Total APP (% of WT)
K
Normalized levels of Nicastrin (% of WT)
n.s.
L
RFP-LC3B
Aβ1-42
M
Levels of Aβ1-42 in autophagosomes (m.g.v./μm²)
N
Extracellular Aβ1-40 (sqrt (pmol/l))
O
Extracellular Aβ1-42 (sqrt (pmol/l))
P
HA-AP-2α WT
HA-AP-2α Mut
BACE1
Q
Mut
R
BACE1 m.g.v./μm²
AP-2αA WT
AP-2αA Mut
S
scr
Bace1 shRNA
mFLAG-BACE1:
70kDa
35kDa
FLAG
GAPDH
T
Normalized levels of BACE1 (% of Scr)
U
WT+mCherry
KO+mCherry
WT+AP-2μ-mRFP
KO+AP-2μ-mRFP
V
# of Aβ1-42 puncta /10 μm of axon
mCherry
AP-2μ-mRFP

Figure EV4.

Figure EV5. AP-2-dependent BACE1 trafficking is required to prevent the neurodegeneration in the brain.

A AP-1γ1 levels in lysates of iPSC-derived neurons from late-onset AD patients carrying the *TREM2 p.R47H* (AD-*TREM2*-2 and AD-*TREM2*-4) variant compared to healthy controls (CON8 and CON9).

B Confocal images of AAV-GFP$^{CamKII\alpha}$-labeled WT and AP-2μ KO dentate gyrus (DG) granule cells. Scale bars: 20 and 5 μm.

C, D Dendritic spine number is significantly reduced in eGFP-expressing AP-2μ KO-cultured neurons when compared to the WT (WT: 4.08 ± 0.30, KO: 2.36 ± 0.38, P = 0.000), 26 WT and 24 KO neurons, N = 3 biological replicates. Scale bar: 2 μm.

E Overview images of WT and AP-2μ KO entorhinal cortex shown in Fig 5E. Scale bar: 5 μm.

F Representative images of dendritic fragments of WT and AP-2μ KO neurons either expressing mCherry or AP-2μ-mRFP and immunostained for PSD95 and Bassoon. Scale bar, 5 μm.

G Overlap coefficient between Bassoon and PSD95 is increased in AP-2μ KO neurons expressing the AP-2μ-mRFP, compared to KO neurons expressing the mCherry (WT^{mChery}: 0.63 ± 0.02, $KO^{mCherry}$: 0.52 ± 0.02, $WT^{AP-2-\mu\text{-}mRFP}$: 0.63 ± 0.02, $KO^{AP-2-\mu\text{-}mRFP}$: 0.60 ± 0.02, $pWT^{mCherry}$ versus $pKO^{mCherry}$ = 0.0003, $pWT^{mCherry}$ versus $pWT^{AP-2-\mu\text{-}mRFP}$>0.999, $pKO^{mCherry}$ versus $pKO^{AP-2-\mu\text{-}mRFP}$ = 0.015, $pWT^{AP-2-\mu\text{-}mRFP}$ versus $pKO^{AP-2-\mu\text{-}mRFP}$ = 0.609, 26–27 neurons per condition, N = 4 biological replicates).

H Representative heatmaps, showing the position of the WT and AP-2μ KO mouse in the experimental arena in the NOR training session.

I No significant difference in the exploration time toward neither object 1 (Obj 1) nor Obj 2 was observed for WT and AP-2μ KO mice during the training period (WT^{obj1}: 50.71 ± 7.34, WT^{obj2}: 49.29 ± 7.85, pWT^{obj1} versus WT^{obj2} = 0.901; KO^{obj1}: 38.48 ± 8.12, KO^{obj2}: 61.52 ± 8.68, pKO^{obj1} versus KO^{obj2} = 0.065; 7 WT and 8 KO mice).

J Total exploration time in NOR test is not altered in AP-2μ KO mice (WT: 45.55 ± 7.24%, KO: 39.63 ± 14.48%, P = 0.733, 7 WT and 8 KO mice).

K Nissl-stained brain sections of WT and AP-2μ KO mice shown in Fig 5M and N. Scale bars: 500 μm. White boxes indicate the area magnified in Fig 5M and N.

L Representative confocal images of WT and AP-2μ KO brains haploinsufficient for BACE1 immunostained for activated caspase-3 and NeuN. Scale bar: 200 μm.

M The number of apoptotic cells is reduced in the hilus of AP-2μ KO mice haploinsufficient for BACE1 ($AP\text{-}2^{WT}/BACE^{WT}$: 1.00 ± 0.58, $AP\text{-}2^{KO}/BACE^{WT}$: 17.67 ± 3.28, $AP\text{-}2^{WT}/BACE^{HET}$: 2.00 ± 0, $AP\text{-}2^{KO}/BACE^{HET}$: 8.00 ± 1.54; $p^{AP-2WT/BACE1WT}$ versus $p^{AP-2KO/BACE1WT}$ = 0.000, $p^{AP-2KO/BACE1WT}$ versus $p^{AP-2KO/BACE1HET}$ = 0.020; $p^{AP-2WT/BACE1HET}$ versus $^{AP-2KO/BACE1HET}$ = 0.153). Three sections from N = 3 mice for each genotype.

Data information: All graphs show mean ± SEM; statistical analysis was performed by unpaired two-tailed Student's t-test in (D, J, I) and two-way ANOVA in (G, M). n.s.—non-significant. * indicates $P \leq 0.05$; ** indicates $P \leq 0.01$; *** indicates $P \leq 0.001$.

 © 2020 The Authors

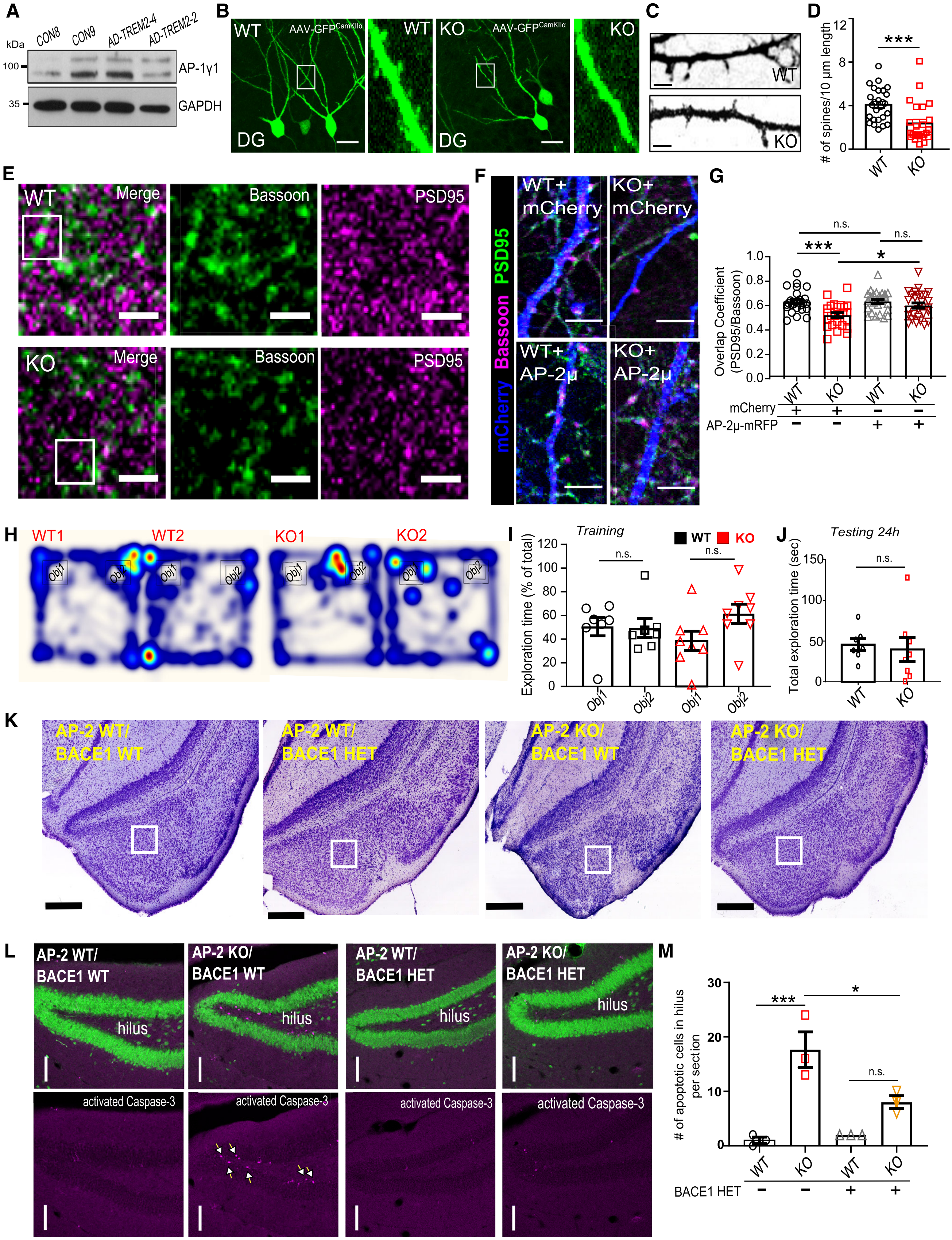
A
kDa
CON8
CON9
AD-TREM2-4
AD-TREM2-2
100
35
AP-1γ1
GAPDH
B
WT
KO
DG
AAV-GFP CamKIIα
C
WT
KO
D
# of spines/10 µm length
***
WT
KO
E
Merge
Bassoon
PSD95
F
WT+ mCherry
KO+ mCherry
WT+ AP-2µ
KO+ AP-2µ
mCherry Bassoon PSD95
G
Overlap Coefficient (PSD95/Bassoon)
n.s.
***
*
mCherry
AP-2µ-mRFP
H
WT1
WT2
KO1
KO2
Obj1
Obj2
I
Training
Exploration time (% of total)
n.s.
Obj1
Obj2
J
Testing 24h
Total exploration time (sec)
K
AP-2 WT/ BACE1 WT
AP-2 WT/ BACE1 HET
AP-2 KO/ BACE1 WT
AP-2 KO/ BACE1 HET
L
hilus
activated Caspase-3
M
# of apoptotic cells in hilus per section
BACE1 HET

Figure EV5.
