# Supplementary material for: AP‐2 reduces amyloidogenesis by promoting BACE1 trafficking and degradation in neurons
Source: EMBO Rep. 2020 Apr 23;21(6):e47954. doi: 10.15252/embr.201947954 (PMC7271323; doi:10.15252/embr.201947954)
Supplement: Supplementary file 2 — Table EV1 [file EMBR-21-e47954-s002.docx]

**Table EV1. Primary and secondary antibodies used in the current study**

| ANTIBODIES | | | | | SOURCE | IDENTIFIER |
| --- | --- | --- | --- | --- | --- | --- |
|  | WB | ICC | IHC |  | |  |
| Mouse monoclonal (AP6)  Anti-AP2α | - | 1:300 | - | Abcam | | Ab2730 |
| Mouse monoclonal (8)  Anti-AP2α | 1:1000 | - | - | BD Transduction | | Cat# 610501 |
| Mouse monoclonal (31)  Anti AP2µ | 1:1000 | - | - | BD Transduction | | Cat# 611350 |
| Mouse monoclonal (88)  Anti AP1γ | 1:1000 | - | - | BD Transduction | | Cat#610385 |
| Mouse monoclonal (Ab40.1)  Anti-Aβ 1-40 | - | 1:100 | - | QED Bioscience Inc | | Cat# 57002 |
| Mouse monoclonal (12F4)  Anti-Aβ 1-42 | - | 1:100 | 1:100 | EMD, Millipore | | Cat# 05-831-I |
| Rabbit polyclonal  Anti-APP | 1:1000 | 1:300 | 1:250 | Sigma-Aldrich | | Cat# A8717 |
| Rabbit polyclonal  Anti-BACE1 | 1:1000 | 1:300 | 1:500 | Thermo Fischer | | Cat# PA1-757 |
| Mouse monoclonal (SAP7F407)  Anti-Bassoon | - | 1:300 | 1:800 | Abcam | | Cat# ab82958 |
| Rabbit polyclonal  Anti-Bassoon | - | 1:300 | - | Synaptic systems | | Cat# 141002 |
| Rabbit polyclonal  Anti-Caspase-3 cleaved | - | - | 1:200 | Cell signaling | | Cat# 9661 |
| Goat polyclonal  Anti-Cathepsin D | - | 1:300 | - | R & D Systems | | Cat# AF1029 |
| Mouse monoclonal (71.1)  Anti-GAPDH | 1:3000 | - | - | Sigma-Aldrich | | Cat# G8795 |
| Rabbit polyclonal  Anti-GABARB3  N-terminal | - | 1:200 | - | Abcam | | Cat#66430 |
| Mouse monoclonal  Anti-GABARB3 (N87/25)  C-terminal | - | 1:200 | - | Abcam | | Cat#ab98968 |
| Chicken polyclonal anti-GFP | - | 1:5000 | - | Abcam | | Cat# ab13970 |
| Mouse monoclonal (16B12) Anti-HA | - | 1:300 | - | Biolegend | | Cat# MMS-101P |
| Rabbit polyclonal  Anti-L1 | 1:1000 | - | - | Aviva Systems Biology | | Cat#OAAB11621 |
| Mouse monoclonal (4E12)  Anti-LC3 | - | 1:300 | - | MBL | | Cat# M152-3B |
| Rabbit polyclonal  Anti-LAMP2a | - | - | 1:500 | Abcam | | Cat# 18528 |
| Rabbit monoclonal (D38F9) Anti-Nicastrin | 1:1000 | - | - | Cell Signaling | | Cat# 5665 |
| Rabbit polyclonal  Anti-PSD95 | - | 1:300 | 1:200 | Protein-Tech  Europe | | Cat# 20665-1-AP |
| Mouse monoclonal (108E10)  Anti-PSD95 | - | 1:300 | - | Synaptic systems | | Cat# 124011 |
| Goat polyclonal  Anti-RAB7A | - | - | 1:250 | Origene | | Cat#AB0033-200 |
| Mouse monoclonal (G10)  Anti-Reelin | - | - | 1:500 | Abcam | | Cat#ab78540 |
| Mouse monoclonal (621.3)  Anti-RAB5 | - | 1:300 | - | SySy | | Cat#108 011 |
| Mouse monoclonal (1C51) Anti-mCherry | - | 1:2000 | - | Novus  Biologicals | | Cat# NBP1-  96752SS |
| Rabbit polyclonal  Anti-mCherry | - | 1:1000 |  | Abcam | | Cat#ab167453 |
| Mouse monoclonal Anti-FLAG | 1:1000 | - | - | Sigma-Aldrich | | Cat# F3165 |
| Goat anti-Mouse IgG (H+L) peroxidase-conjugated | 1:5000 | - | - | Jackson ImmunoResearch | | Cat# 115-035-  003 |
| Goat anti-Rabbit IgG (H+L) peroxidase-conjugated | 1:5000 | - | - | Jackson ImmunoResearch | | Cat# 111-035-  003 |
| Alexa Fluor 488 Goat anti-Mouse IgG | - | 1:500 | 1:500 | Thermo Fisher | | Cat# A-11029 |
| Alexa Fluor 488 Goat anti-Rabbit IgG | - | 1:500 | 1:500 | Thermo Fisher | | Cat# A-11034 |
| Alexa Fluor 488 Goat anti-Chicken IgG | - | 1:500 | - | Thermo Fisher | | Cat# A-11039 |
| Alexa Fluor 568 Goat anti-Mouse IgG | - | 1:500 | 1:500 | Thermo Fisher | | Cat# A-11031 |
| Alexa Fluor 568 Goat anti-Rabbit IgG | - | 1:500 | 1:500 | Thermo Fisher | | Cat# A-11036 |
| Alexa Fluor 647 Goat anti-Mouse IgG | - | 1:500 | - | Thermo Fisher | | Cat# A-21236 |
| Alexa Fluor 647 Goat anti-Rabbit IgG | - | 1:500 | - | Thermo Fisher | | Cat# A-21245 |
| Normal Rabbit IgG | - | - | - | Cell Signalling | | Cat# 2729 |
| Normal Mouse IgG | - | - | - | Merck Millipore | | Cat# 12-371 |
