# Supplementary material for: AP‐2 reduces amyloidogenesis by promoting BACE1 trafficking and degradation in neurons
Source: EMBO Rep. 2020 Apr 23;21(6):e47954. doi: 10.15252/embr.201947954 (PMC7271323; doi:10.15252/embr.201947954)
Supplement: Supplementary file 3 — Table EV2 [file EMBR-21-e47954-s003.docx]

**Table EV2. Vectors used in the current study**

| Recombinant DNA | | SOURCE | IDENTIFIER | |
| --- | --- | --- | --- | --- |
| Plasmids | | | | |
| pEGFP-N | Kind gift from Dr. M. Kreutz | | | N/A |
| pmCherry-N | Kind gift from Dr. M. Kreutz | | | N/A |
| mKeima-Red | Addgene | | | #54597 |
| eBFP2-N1 | Kind gift from Dr. S. Iden | | | N/A |
| pTagRFP-C | Kind gift from Dr. M. Kreutz | | | N/A |
| pTagRFP-C-LC3BG120 | This paper | | | N/A |
| HA-BACE1-eGFP | Kind gift from Prof. Juan Bonifacino | | | N/A |
| HA-BACE1-LL/AA-eGFP | Kind gift from Prof. Juan Bonifacino | | | N/A |
| BACE1-eGFP | Origene | | | RG209115 |
| HA-BACE1-mCherry | This paper | | | N/A |
| BACE1-mKeima-Red | This paper | | | N/A |
| FLAG-BACE1 | Sino Biological, Inc | | | #MG50002-NF |
| AP2-µ-mRFP | (Kononenko et al, 2017) | | | N/A |
| AP2-µ-mCherry | (Kononenko et al, 2017) | | | N/A |
| HA-AP-2α_A_ | (Kononenko et al., 2017) | | | N/A |
| HA-AP-2α_A_ Mut | (Kononenko et al., 2017) | | | N/A |
| eGFP-RAB4 | Addgene | | | #49468 |
| eGFP-RAB4-S22N | Addgene | | | #49887 |
| eGFP-RAB11A | Kind gift from Prof. Volker Haucke | | | N/A |
| eGFP- RAB11A-S25N | This paper | | | N/A |
| eGFP-RAB5-Q79L | This paper | | | N/A |
| mCherry-APP-P1-eGFP | Kind gift from Dr. Martino Calamai | | | N/A |
| mCherry-APP-eGFP | Kind gift from Dr. Martino Calamai | | | N/A |
| AAV9-*CamKIIα*-eGFP | Penn Vector Core. University of Pennsylvania School of Medicine | | | AV-9-pV1917 |
| sh-NLS-RFP-BACE1 | This paper | | | N/A |
| scr-NLS-RFP-BACE1 | This paper | | | N/A |
