# Supplementary material for: AP‐2 reduces amyloidogenesis by promoting BACE1 trafficking and degradation in neurons
Source: EMBO Rep. 2020 Apr 23;21(6):e47954. doi: 10.15252/embr.201947954 (PMC7271323; doi:10.15252/embr.201947954)
Supplement: Supplementary file 4 — Table EV3 [file EMBR-21-e47954-s004.docx]

**Table EV3. Oligonucleotides and shRNA sequences used in the current study**

| Oligonucleotides | SOURCE | IDENTIFIER | |
| --- | --- | --- | --- |
| Scr siRNA:  5´-GUAACUGUCGGCUCGUGGU(dTdT)-3` | (Diril et al., 2009) | | N/A |
| siRNA *AP-2μ*:  5`-GUGGAUGCCUUUCGGGUCA (dTdT)-3` | (Janvier and Bonifacino, 2005) | | N/A |
| *Bace1* shRNA: TGGACTGCAAGGAGTACAA | (Singer et al, 2005) | | N/A |
| scrambled shRNA: CGTACATGGAACTGAAGAG | (Singer et al, 2005) | | N/A |
| Primer: *Bace1* Forward: TTGTAGGGCTAGGGATGGTC | This paper | | N/A |
| Primer: *Bace1* Reverse: CCTAACCCTGCTGGATGATA | This paper | | N/A |
| Primer: *Gapdh* Forward: AACTTTGGCATTGTGGAAGG | (Kye et al, 2011) | | N/A |
| Primer: *Gapdh* Reverse: ACACATTGGGGGTAGGAACA | (Kye et al, 2011) | | N/A |
