# Supplementary material for: AP‐2 reduces amyloidogenesis by promoting BACE1 trafficking and degradation in neurons
Source: EMBO Rep. 2020 Apr 23;21(6):e47954. doi: 10.15252/embr.201947954 (PMC7271323; doi:10.15252/embr.201947954)
Supplement: Supplementary file 5 — Table EV4 [file EMBR-21-e47954-s005.docx]

**Table EV4. Summary of the Healthy Controls and AD iPSC lines used in this study**

| **iPSCs name** | **Status** | **AD risk variant** | **Age** | **Age**  **at onset** | **Gender** | **APOE genotype** | **Reference** |
| --- | --- | --- | --- | --- | --- | --- | --- |
| CON8 | Control individual | Control | 69 | - | M | 3/4 | (Schröter et al, 2016a) |
| CON9 | Control individual | Control | 75 | - | F | 3/3 | (Martins et al, 2018) |
| AD-TREM2-2 | AD patient | TREM2 p.R47H heterozygous | 65 | 60 | M | 4/4 | (Schröter et al, 2016c) |
| AD-TREM2-4 | AD patient | TREM2 p.R47H heterozygous | 67 | 64 | F | 2/4 | (Schröter et al, 2016e) |
