# Supplementary material for: AP‐2 reduces amyloidogenesis by promoting BACE1 trafficking and degradation in neurons
Source: EMBO Rep. 2020 Apr 23;21(6):e47954. doi: 10.15252/embr.201947954 (PMC7271323; doi:10.15252/embr.201947954)
Supplement: Supplementary file 6 — Movie EV1 [file EMBR-21-e47954-s006.zip › EMBOR-2019-47954V2_MovieEV1/EMBOR-2019-47954V2_Movie EV1_Legend.docx]

**Movie S1.** Representative movie illustrating the co-trafficking of AP-2μ-mCherry (red) and HA-BACE1-eGFP (green) in axons. Resolution: 6 frames per second.
