# Supplementary material for: AP‐2 reduces amyloidogenesis by promoting BACE1 trafficking and degradation in neurons
Source: EMBO Rep. 2020 Apr 23;21(6):e47954. doi: 10.15252/embr.201947954 (PMC7271323; doi:10.15252/embr.201947954)
Supplement: Supplementary file 7 — Movie EV2 [file EMBR-21-e47954-s007.zip › EMBOR-2019-47954V2_MovieEV2/EMBOR-2019-47954V2_MovieEV2_Legend.docx]

**Movies S2.** Representative movie illustrating the dynamics of HA-BACE1-eGFP-positive carriers in the axons of WT (upper panel) and AP-2µ KO (lower panel) neurons. Resolution: 10 frames per second.
