# Supplementary material for: AP‐2 reduces amyloidogenesis by promoting BACE1 trafficking and degradation in neurons
Source: EMBO Rep. 2020 Apr 23;21(6):e47954. doi: 10.15252/embr.201947954 (PMC7271323; doi:10.15252/embr.201947954)
Supplement: Supplementary file 9 — Review Process File [file EMBR-21-e47954-s014.pdf]

EMBO
reports

AP-2 reduces amyloidogenesis by promoting BACE1 trafficking and degradation in neurons

Sujoy Bera, Santiago Camblor-Perujo, Elena Calleja Barca, Albert Negrete-Hurtado, Julia Racho, Elodie De Bruyckere, Christoph Wittich, Nina Ellrich, Soraia Martins, James Adjaye & Natalia L. Kononenko

Review timeline:

Submission date:	22 August 2019
Editorial Decision:	14 October 2019
Revision received:	10 February 2020
Editorial Decision:	5 March 2020
Revision received:	11 March 2020
Accepted:	13 March 2020

Editor: Esther Schnapp

Transaction Report:

(Note: With the exception of the correction of typographical or spelling errors that could be a source of ambiguity, letters and reports are not edited. The original formatting of letters and referee reports may not be reflected in this compilation.)

1st Editorial Decision 14 October 2019

Thank you for the submission of your manuscript to EMBO reports. It was sent to three referees, but unfortunately, only 2 of them sent us their comments that are pasted below. I will make a decision now based on these 2 reports in order to save you from losing more time.

As you will see, both referees acknowledge that the findings are potentially interesting. However, both also point out that significant revisions will be required to strengthen the data for publication here. As I said, it will be important to clarify whether the observed AP-2 KO phenotypes are due to global effects on trafficking, or to specific defects in BACE1 trafficking, as both referees mention. All other concerns will also need to be addressed. Please let me know if you feel that any of the referee concerns are not directly relevant for the main focus of this study.

I would thus like to invite you to revise your manuscript with the understanding that the referee concerns must be fully addressed and their suggestions taken on board. Please address all referee concerns in a complete point-by-point response. Acceptance of the manuscript will depend on a positive outcome of a second round of review. It is EMBO reports policy to allow a single round of major revision only and acceptance or rejection of the manuscript will therefore depend on the completeness of your responses included in the next, final version of the manuscript.

Revised manuscripts should be submitted within three months of a request for revision; they will otherwise be treated as new submissions. Please contact us if a 3-months time frame is not sufficient for the revisions so that we can discuss this further. Given the 6 main figures, I suggest that you layout the manuscript as a full article.

Regarding data quantification, please specify the number "n" for how many independent experiments were performed, the bars and error bars (e.g. SEM, SD) and the test used to calculate p-values in the respective figure legends. This information must be provided in the figure legends. Please also include scale bars in all microscopy images.

When submitting your revised manuscript, please carefully review the instructions that follow below. Failure to include requested items will delay the evaluation of your revision.

1) a .docx formatted version of the manuscript text (including legends for main figures, EV figures and tables). Please make sure that the changes are highlighted to be clearly visible.

2) individual production quality figure files as .eps, .tif, .jpg (one file per figure).
See https://wol-prod-cdn.literatumonline.com/pb-assets/embo-site/EMBOPress_Figure_Guidelines_061115-1561436025777.pdf for more info on how to prepare your figures.

3) We replaced Supplementary Information with Expanded View (EV) Figures and Tables that are collapsible/expandable online. A maximum of 5 EV Figures can be typeset. EV Figures should be cited as 'Figure EV1, Figure EV2" etc... in the text and their respective legends should be included in the main text after the legends of regular figures.

- For the figures that you do NOT wish to display as Expanded View figures, they should be bundled together with their legends in a single PDF file called *Appendix*, which should start with a short Table of Content. Appendix figures should be referred to in the main text as: "Appendix Figure S1, Appendix Figure S2" etc. See detailed instructions regarding expanded view here: <https://www.embopress.org/page/journal/14693178/authorguide#expandedview>

- Additional Tables/Datasets should be labeled and referred to as Table EV1, Dataset EV1, etc. Legends have to be provided in a separate tab in case of .xls files. Alternatively, the legend can be supplied as a separate text file (README) and zipped together with the Table/Dataset file.

4) a .docx formatted letter INCLUDING the reviewers' reports and your detailed point-by-point responses to their comments. As part of the EMBO Press transparent editorial process, the point-by-point response is part of the Review Process File (RPF), which will be published alongside your paper.

5) a complete author checklist, which you can download from our author guidelines <https://www.embopress.org/page/journal/14693178/authorguide>. Please insert information in the checklist that is also reflected in the manuscript. The completed author checklist will also be part of the RPF.

6) Please note that all corresponding authors are required to supply an ORCID ID for their name upon submission of a revised manuscript (<https://orcid.org/>). Please find instructions on how to link your ORCID ID to your account in our manuscript tracking system in our Author guidelines <https://www.embopress.org/page/journal/14693178/authorguide#authorshipguidelines>

7) We would also encourage you to include the source data for figure panels that show essential data. Numerical data should be provided as individual .xls or .csv files (including a tab describing the data). For blots or microscopy, uncropped images should be submitted (using a zip archive if multiple images need to be supplied for one panel). Additional information on source data and instruction on how to label the files are available at <https://www.embopress.org/page/journal/14693178/authorguide#sourcedata>.

8) Our journal also encourages inclusion of *data citations in the reference list* to directly cite datasets that were re-used and obtained from public databases. Data citations in the article text are distinct from normal bibliographical citations and should directly link to the database records from which the data can be accessed. In the main text, data citations are formatted as follows: "Data ref: Smith et al, 2001" or "Data ref: NCBI Sequence Read Archive PRJNA342805, 2017". In the Reference list, data citations must be labeled with "[DATASET]". A data reference must provide the database name, accession number/identifiers and a resolvable link to the landing page from which the data can be accessed at the end of the reference. Further instructions are available at https://www.embopress.org/page/journal/14693178/authorguide#referencesformat

We would also welcome the submission of cover suggestions, or motifs to be used by our Graphics

Illustrator in designing a cover.

As part of the EMBO publication's Transparent Editorial Process, EMBO reports publishes online a Review Process File (RPF) to accompany accepted manuscripts. This File will be published in conjunction with your paper and will include the referee reports, your point-by-point response and all pertinent correspondence relating to the manuscript.

You are able to opt out of this by letting the editorial office know (emboreports@embo.org). If you do opt out, the Review Process File link will point to the following statement: "No Review Process File is available with this article, as the authors have chosen not to make the review process public in this case."

I look forward to seeing a revised version of your manuscript when it is ready. Please let me know if you have questions or comments regarding the revision.

REFEREE REPORTS

Referee #1:

Endocytic regulation of amyloidogenesis is a highly investigated area given that its dysfunction may be a primary event in Alzheimer's disease. The adaptor protein 2 is a clathrin adaptor protein and a central player in the mechanism of endocytosis dependent on clathrin for the formation of endocytic vesicles. APP has been described to endocytosed via clathrin-mediated endocytosis, while BACE1 endocytosis may be independent of clathrin although this remains controversial. APP and BACE1 endocytosis are required for beta-amyloid production. Thus, knowing the role of AP-2 in APP and BACE1 trafficking is crucial to identify novel therapeutic targets to control amyloidogenesis.
In this paper, the authors use a strategy of conditional KO for AP2 to provide evidence for BACE1 endocytosis being independent of AP2, supporting a mechanism of BACE1 internalization independent of clathrin that was previously proposed by Wim Annaert lab. For the first time AP2 is implicated in the trafficking of BACE1 to the lysosome, possibly through regulating BACE1 retrograde transport from axonal terminal to the soma where most degradative lysosomes are located. The lack of AP2 induces the distal accumulation of BACE1 in late-endosomes/autophagosomes where it encounters APP potentiating amyloid beta accumulation.
The first general concern relates with the experimental design regarding the use of conditional KO for AP2. Tamoxifen treatment lasted 11-12 days starting immediately after plating, or for 5-7 days in p14 mice. During this period neurons are differentiating axons, and dendrites, axons grow, and synapses start to form. Given these determinant events it is surprising that the neuronal morphology was generally unaffected by AP2 KO given the important role of CME. Could there be a potential compensatory expression of other proteins of the same family such as AP1 and AP3? AP1 and AP3 levels should be controlled for. Or even better a more acute inhibition of AP2 could be achieved by using an AP2 interfering peptide as described previously by Morgan Sheng lab and others (for ex https://www.sciencedirect.com/science/article/pii/S0896627302010243).
The second general concern refers to the indication in the methods that the neurons die after 11-12 days in vitro. This could indicate that the authophagosome accumulation in distal neurons and the loss of synapses could be related to a more general degenerating process than to a specific defect in BACE1 trafficking. These defects could be mediated by amyloid beta increased production in stressed cells. To rule these doubts I would suggest a rescue of amyloid accumulation and of synapse loss by re-expressing AP2 as done for transferrin endocytosis in Fig.S3A,B.
Structure: The paper is well structured although the emphasis on a possible role for AP2 in BACE1 recycling in figure 1 misleads the reader in face of the more striking phenotype of BACE1 accumulation in AP2 KO neurons that is only presented in figure 2 and explored in the remaining figures.

Abstract: In the abstract the authors extrapolate that AP2 is largely dispensable for BACE1 endocytosis but there is only one experiment supporting this statement and the images presented do not allow for a proper evaluation of the phenotype. Also, AP2 likely plays a role in amyloidosis by controlling APP endocytosis. Without the controls mentioned in general concern I would recommend toning down this affirmation.
What is better supported by the data is the role of AP2 beyond the internalization step during

endosomal maturation and delivery of BACE1 to the lysosome. That lack of AP2 induces BACE1 accumulation in late endosomes and autophagosomes which potentiates amyloid beta production.

Introduction:
It misses some background on AP2, on its isoforms, known functions in the endocytic pathway after internalization, known specific functions in neurons...

Results:
1. P5 l 110 "WT and KO neurons transiently expressing the HA-BACE1-eGFP that has a localization similar to endogenous BACE1 (Fig. 112 S1C) ...." This is an overinterpretation, since the anti-BACE1 antibody will recognize primarily exogenous BACE1 in addition to endogenous BACE1. Thus, I suggest that the distribution of exogenous HA-BACE1-GFP alone be compared with that of endogenous BACE1 alone. Including in a separate panel an image of a neuron with a similar morphology immunolabeled with anti-BACE1.
2. P5 l 118 "Surprisingly, AP-2μ was not required for BACE1 endocytosis 5 min after the beginning of the assay, suggesting that BACE1 endosomal recycling, but not endocytosis, might be AP-2-dependent." This is an important statement that needs to be better supported. It should be included in figure 1 the representative images for time point 5 as done for time point 20 min, with the endocytosed signal shown alone in a panel with the magnification of the axonal segment.
3. P6 l131 "...indicating that in the absence of AP-2 BACE1 is mistrafficked between the cell surface and the endosomal system ..." This sentence would be clearer if it read that BACE1 is mistrafficked between endosomes and the plasma membrane..."
4. P6 l 133 "To directly test whether AP-2μ loss facilitates endosomal recycling of BACE1,..." the authors directly test if blocking BACE1 recycling corrects the potentiating effect of AP2 KO on BACE1 recycling. This should be clarified.
5. P10 l228 "Since axonal BACE1 is retrogradely transported in autophagosomes (Feng et al., 2017)..." This is an overstatement that needs to be toned down. The lab of Rajendran and Thinakaran have clearly shown that the majority of BACE1 is transported in recycling carriers positive for rab11. Feng et al, show that in wt neurons a small fraction of BACE1 carriers are autophagosomes.
6. Moreover, the authors show a generalized decrease in transport and not only in retrograde transport. This should be interpreted.
7. P11l237 "Together, these data suggest that AP-2 regulates BACE1 protein levels via mediating autophagosomal transport of BACE1 in axons." This is an overstatement because in AP2 KO neurons autophagosomes may form due to BACE1 accumulation in axons due to defects in transport while in and not due to AP2 mediation of autophagossomal transport. Moreover, in wt cells (panel 3H) one appreciates that only a fraction of bace1 vesicles is positive for lc3, even with lc3 being overexpressed, which may on its own increase the number of autophagosomes.
8. L286 typo BACE1-dependent
9. L303 please clarify why TREM2 mutant lines? Do the authors link the mutation to AP2 reduction?
10. L337 "...and requires its association with LC3-containing autophagosomes,..." the authors should explain which experiment shows the requirement of the association of AP2 with LC3 autophagosomes.
Discussion:
It would be important to discuss what happens to CME in neurons when AP2 is KO.

Figures:
Fig1F and I the channel for rab should be shown alone and merged in order to evaluate the presence of rab4 and rab4SN in BACE1 vesicles.
Figure 1H, the authors used eGFP to control Rab11-S25N but an additional control should be rab11 wild type.
figure 1 K-M, the authors should also present the total BACE1 quantification, since BACE1 total levels were found increased in vivo (Fig.2A-B).
Fig2Q-S The data show nicely a decrease of BACE1 in acidic compartments (red) but not the increase in non-acidic compartments (green). Can you explain why not? Or find a better image to represent the data.
FigS3E the levels of beta-amyloid need to be quantified.
Fig3A,B show an important colocalization between exogenous ap2 and bace1, this should be complemented with the colocalization between endogenous proteins.

Fig.4D Label panel D with "APP plus CTFs". Or clarify that the antibody used recognizes exclusively APP CTFs but not the full length.
Fig.4T Although the representative images show reduction in ab42 signal when BACE1 is KD in the graph there is no significant difference in wt neurons KD for BACE1. How do the authors explain this inconsistency?
Fig5C Misses the quantification of number of spines.
Fig.5K Please indicate that panel K refers to BACE1 KD

Referee #2:

This manuscript reports a detailed investigation of the role of AP-2 in the trafficking of BACE1 and its downstream consequences on APP processing and neuronal function. The work is thorough and overall is well performed and controlled. The manuscript contains a considerable amount of data within it, which in places makes it fairly dense and difficult to read. Positive aspects include a series of detailed and complementary assays to conclusively demonstrate the role of AP-2 in intracellular BACE1 trafficking. Weaker points include the lack of demonstration of specificity in terms of BACE1 trafficking. Also in a few places, the experimental n that has been chosen to show significance should be reassessed.

Major points
1) The authors convincingly show that reduction of BACE1 levels can correct the observed dysfunction in the AP-2 knockout mice and neurons. However, an unaddressed question is whether the effects are due to global effects on post-endocytosis trafficking to lysosomes in AP-2 knockout mice, or whether it is specific to disrupted BACE1 traffic. The di-leucine mutant mentioned in the manuscript could address this point, since AP-2 is dispensable for BACE1 endocytosis (Fig 1A,B). Use of this mutant therefore should reveal whether the altered trafficking and subsequent downstream effects were BACE1-specific and or a generalised defect in post-endocytosis trafficking with altered BACE1 trafficking a consequence of that. This mutant could be employed in trafficking assays such as in Figures 1-3, and APP processing experiments in Figure 4 to address this point.

2) Related to the point above, are the total protein levels, internalisation kinetics or surface fraction of other cargo molecules unaltered by the absence of AP-2?

3) Again related to specificity, are other BACE1 substrates similarly affected in the absence of AP-2, or is this defect specific to APP cleavage?

4) Many of the experiments presented in this manuscript are display relatively modest effects, which are significant due to the high power provided by using either individual neurons or axonal lengths as experimental n. Individual neurons could be viewed as being the major source of variation in this type of analysis and therefore acceptable as experimental n. However, I do not really think the same can be said for axonal lengths (and definitely not puncta, such as used in Figure 4B). The authors should try to present their data using at least the neuron as the basic unit for statistical analysis wherever possible.

Minor points
1) Fig 1A suggests that BACE1 is internalised in an AP-2 independent manner. Can the authors speculate as to how BACE1 internalisation (and downstream effects) will be altered during neuronal activity? For example, would any effect be exacerbated, since there will be greater BACE1 turnover and hence more Abeta production (leading to a positive feedback loop of increased hyperexcitability and further BACE1 internalisation)?

2) Figure 1P is not referenced in the text.

3) How specific is the decrease in AP-2 in human patient iPSCs? Are other adaptor protein complexes decreased too?

1st Revision - authors' response 10 February 2020

Reviewer #1 (Remarks to the Author):

Endocytic regulation of amyloidogenesis is a highly investigated area given that its dysfunction may be a primary event in Alzheimer's disease. The adaptor protein 2 is a clathrin adaptor protein and a central player in the mechanism of endocytosis dependent on clathrin for the formation of endocytic vesicles. APP has been described to endocytosed via clathrin-mediated endocytosis, while BACE1 endocytosis may be independent of clathrin although this remains controversial. APP and BACE1 endocytosis are required for beta-amyloid production. Thus, knowing the role of AP-2 in APP and BACE1 trafficking is crucial to identify novel therapeutic targets to control amyloidogenesis.
In this paper, the authors use a strategy of conditional KO for AP2 to provide evidence for BACE1 endocytosis being independent of AP2, supporting a mechanism of BACE1 internalization independent of clathrin that was previously proposed by Wim Annaert lab. For the first time AP2 is implicated in the trafficking of BACE1 to the lysosome, possibly through regulating BACE1 retrograde transport from axonal terminal to the soma where most degradative lysosomes are located. The lack of AP2 induces the distal accumulation of BACE1 in late-endosomes/autophagosomes where it encounters APP potentiating amyloid beta accumulation.

Response: We thank the Reviewer 1 for this positive comment on the novelty of our work.

The first general concern relates with the experimental design regarding the use of conditional KO for AP2. Tamoxifen treatment lasted 11-12 days starting immediately after plating, or for 5-7 days in p14 mice. During this period neurons are differentiating axons, and dendrites, axons grow, and synapses start to form. Given these determinant events it is surprising that the neuronal morphology was generally unaffected by AP2 KO given the important role of CME.

Response: We understand and appreciate the reviewer's concern regarding the role of AP-2 in neuronal morphology. Current study is a follow-up on two studies published in Neuron (Kononenko et al., 2014) and Nat. Commun. 2017 (Kononenko et al., 2017), where we characterized in detail the requirement of AP-2 for synaptic vesicles endocytosis and neuronal morphology. We have found that AP-2 is not required for CME at the synapse (Kononenko et al., 2014), but regulates neuronal complexity in-vitro and in-vivo (Kononenko et al., 2017). In Kononenko et al, 2017 we have compared the effects of AP-2 loss-of-function on neuronal complexity by applying Tamoxifen either immediately after plating or at DIV8, when neuronal differentiation is mostly completed (Kononenko et al., 2017). Conditional loss of AP-2µ, induced either at DIV8 or at DIV0, significantly impaired the neuronal branching complexity of neurons (Fig. S6j-l). Subsequently, we have found that the effect on neuronal morphology is not due to a developmental delay/defect, but a result of neurodegeneration (Kononenko et al., 2017). We apologize for not making this clear in the introduction part of current study. We have now inserted this information in the manuscript pages 4-5.
Additionally, to exclude the role of AP-2 in development and acutely measure the effect of AP-2µ loss on neuronal function, we have introduced a completely new experiment, where we delivered Cre under CamKIIalpha promoter to the dentate gyrus of the hippocampus of AP-2flox/flox mice at 9 weeks of age. As indicated in Fig.5C,D, KO of AP-2 in mature brain significantly decreases neuronal spine density, a phenotype which is in line with other data presented here.

Could there be a potential compensatory expression of other proteins of the same family such as AP1 and AP3? AP1 and AP3 levels should be controlled for.

Response: We understand and appreciate the reviewer's concern regarding the potential compensatory expression of other endocytic adaptors. This was analyzed in detail in Kononenko et al., 2014 (Fig. S2I) and Kononenko et al., 2017 (Fig. S3c,d). The levels of AP-1 and AP-3 are not altered in AP-2 KO neurons. In Kononenko et al., 2017 we have also analyzed the levels of other endocytic proteins such as CHC, ITSN1, Amph1, Dyn1, Stn2 upon loss of AP-2. For none of mentioned proteins we detected significant changes in protein expression. We apologize for not making this clear in the manuscript. We have now inserted this information in the manuscript, pages 5 and 8.

© European Molecular Biology Organization

Or even better a more acute inhibition of AP2 could be achieved by using an AP2 interfering peptide as described previously by Morgan Sheng lab and others (for ex https://www.sciencedirect.com/science/article/pii/S0896627302010243).

Response: We thank the Reviewer 1 for this suggestion. As stated above, in Kononenko et al, 2017 we have already compared the effects of AP-2 loss on neuronal complexity by applying Tamoxifen either immediately after plating or at DIV8, when neuronal differentiation is mostly completed (Kononenko et al., 2017). Conditional loss of AP-2µ induced either at DIV8 or at DIV0 significantly impaired the neuronal branching complexity of neurons (Fig. S6j-l).

To address the reviewer's concern in detail, we initially teamed up together with Prof. Ines Neundorf (Inst. of Biochemistry, Cologne, Horn & Neundorf, 2018), who is an expert in cell permeable peptide synthesis. However, we experienced a trouble with the peptide production and could only obtain a small amount of interfering peptide and a scramble control, which was enough for one experiment. To test if interfering with BACE1 binding to AP-2 will inhibit BACE1 transport kinetics, we treated DIV12 control neurons overexpressing HA-BACE1-eGFP with GLRKRLRKFRNKDDISLL peptide, which will block AP-2 interaction (100µM for 20 min), and afterwards performed the live-cell imaging. As the Reviewer can appreciate from the figure below, we could obtain a significant decrease in BACE1 retrograde (but not anterograde) velocity. We feel however that it will be not proper to include the data from N=1 biological replicates in the manuscript.

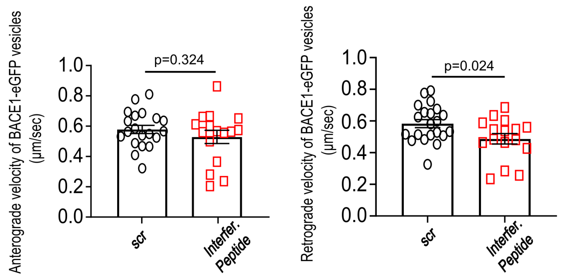

Thus, to reliably show that AP-2 is required for BACE1 trafficking, we followed an alternative strategy (also suggested by the Reviewer 2). We have acutely interfered with AP-2-dependent BACE1 trafficking in control neurons by taking advantage of BACE1 di-leucine mutant plasmid. We overexpressed this mutant in wild type neurons and found that BACE1 requires the interaction with AP-2 for its axonal trafficking (Fig. 3L-N), endosomal recycling and delivery to lysosomes (Fig. S3R-U). Furthermore, we now show that APP processing (Fig. 4U,V) is severely altered in neurons overexpressing the BACE1 di-leucine mutant.

The second general concern refers to the indication in the methods that the neurons die after 11-12 days in vitro. This could indicate that the authophagosome accumulation in distal neurons and the loss of synapses could be related to a more general degenerating process than to a specific defect in BACE1 trafficking. These defects could be mediated by amyloid beta increased production in stressed cells. To rule these doubts I would suggest a rescue of amyloid accumulation and of synapse loss by re-expressing AP2 as done for transferrin endocytosis in Fig.S3A,B.

Response: We thank the Reviewer 1 for this comment. We have gladly followed his/her suggestion and performed rescue experiments of amyloid beta accumulation and synapse loss upon AP-2 re-expression. The data, illustrated in Figs. S4U,V and S5F-I, reveal that both phenotypes are rescued upon reintroducing of AP-2 into neurons. This indicates that AP-2 is directly responsible for synapse maintenance and functions to prevent the amyloidogenesis.

Structure: The paper is well structured although the emphasis on a possible role for AP2 in BACE1 recycling in figure 1 misleads the reader in face of the more striking phenotype of BACE1 accumulation in AP2 KO neurons that is only presented in figure 2 and explored in the remaining figures.

© European Molecular Biology Organization

Response: We thank the Reviewer 1 for this positive comment on our manuscript. We structured the paper in this way, since (as also mentioned by the reviewer him/herself) AP-2 has a prominent role in cargo endocytosis and recycling.

Abstract: In the abstract the authors extrapolate that AP2 is largely dispensable for BACE1 endocytosis but there is only one experiment supporting this statement and the images presented do not allow for a proper evaluation of the phenotype. Also, AP2 likely plays a role in amyloidosis by controlling APP endocytosis. Without the controls mentioned in general concern I would recommend toning down this affirmation.
What is better supported by the data is the role of AP2 beyond the internalization step during endosomal maturation and delivery of BACE1 to the lysosome. That lack of AP2 induces BACE1 accumulation in late endosomes and autophagosomes which potentiates amyloid beta production.

Response: We thank the Reviewer 1 for this comment and have modified the abstract accordingly and it reads now: "AP-2 prevents amyloidogenesis by functioning downstream of BACE1 endocytosis, regulating BACE1 endosomal trafficking and its delivery to lysosomes."

Introduction:
It misses some background on AP2, on its isoforms, known functions in the endocytic pathway after internalization, known specific functions in neurons...

Response: We have now included a paragraph describing the function of AP-2 in CME, as well as described AP-2 isoforms and its specific requirement in neurons (page 5).

Results:
1. P5 l 110 "WT and KO neurons transiently expressing the HA-BACE1-eGFP that has a localization similar to endogenous BACE1 (Fig. 112 S1C) ...." This is an overinterpretation, since the anti-BACE1 antibody will recognize primarily exogenous BACE1 in addition to endogenous BACE1. Thus, I suggest that the distribution of exogenous HA-BACE1-GFP alone be compared with that of endogenous BACE1 alone. Including in a separate panel an image of a neuron with a similar morphology immunolabeled with anti-BACE1.

Response: We thank the reviewer for this constructive suggestion. We have now introduced a separate panel in Fig. S1D, where the expression of endogenous BACE1 is compared to HA-BACE1-eGFP alone.

2. P5 l 118 "Surprisingly, AP-2μ was not required for BACE1 endocytosis 5 min after the beginning of the assay, suggesting that BACE1 endosomal recycling, but not endocytosis, might be AP-2-dependent." This is an important statement that needs to be better supported. It should be included in figure 1 the representative images for time point 5 as done for time point 20 min, with the endocytosed signal shown alone in a panel with the magnification of the axonal segment.

Response: We have now introduced the images for 5min of BACE1 internalization, as suggested by the reviewer. These images are now shown in Fig. 1B.

3. P6 l131 "...indicating that in the absence of AP-2 BACE1 is mistrafficked between the cell surface and the endosomal system ..." This sentence would be clearer if it read that BACE1 is mistrafficked between endosomes and the plasma membrane..."

Response: We thank the reviewer for this constructive suggestion. We adjusted the sentence accordingly and the sentence reads now as following: " indicating that in the absence of AP-2 BACE1 is mistrafficked between endosomes and the plasma membrane..".

4. P6 l 133 "To directly test whether AP-2μ loss facilitates endosomal recycling of BACE1,..." the authors directly test if blocking BACE1 recycling corrects the potentiating effect of AP2 KO on BACE1 recycling. This should be clarified.

Response: We thank the reviewer for this suggestion. We adjusted the sentence accordingly and the sentence reads now as following: "To directly test whether blocking endosomal recycling in neurons lacking AP-2 restores BACE1 trafficking"

5. P10 l228 "Since axonal BACE1 is retrogradely transported in autophagosomes (Feng et al., 2017)..." This is an overstatement that needs to be toned down. The lab of Rajendran and Thinakaran have clearly shown that the majority of BACE1 is transported in recycling carriers positive for rab11. Feng et al, show that in wt neurons a small fraction of BACE1 carriers are autophagosomes.

Response: We thank the reviewer for this suggestion. We adjusted the sentence, which reads now as following: "Since a fraction of axonal BACE1 is known to be retrogradely transported in autophagosomes".

6. Moreover, the authors show a generalized decrease in transport and not only in retrograde transport. This should be interpreted.

Response: We thank the reviewer for this comment. We believe that our data are in line with recent studies in C. elegans, which have shown the defect in anterograde delivery of Glutamate receptor GLR1 to the plasma membrane (Garafalo et al, 2015). To illustrate this we have now introduced the following sentence in the discussion, page 20: "In fact, since in C. elegans AP-2 has been reported to control the anterograde delivery of glutamate receptor GLR1 to the plasma membrane (Garafalo et al, 2015), our data suggest that the generalized defect of AP-2 loss on BACE1 trafficking might be due to additional, yet undescribed AP-2 functions in the secretory pathway in mammalian neurons"

7. P11l237 "Together, these data suggest that AP-2 regulates BACE1 protein levels via mediating autophagosomal transport of BACE1 in axons." This is an overstatement because in AP2 KO neurons autophagosomes may form due to BACE1 accumulation in axons due to defects in transport while in and not due to AP2 mediation of autophagossomal transport. Moreover, in wt cells (panel 3H) one appreciates that only a fraction of bace1 vesicles is positive for lc3, even with lc3 being overexpressed, which may on its own increase the number of autophagosomes.

Response: We thank the reviewer for the constructive comment. In our previous work (Kononenko et al., 2017) we have shown that AP-2 mediates axonal transport of autophagosomes from distal axonal terminals to the cell soma by interacting with autophagy modifier LC3 and Dynactin/p150-Glued subunit of dynein retrograde transport machinery. We believe that these published data, taken together with the fact that LC3-binding deficient mutant of AP-2 mimics BACE1 trafficking defect (Fig. 3K) suggest that AP-2-dependent autophagosome transport regulates BACE1 trafficking.

However, we fully agree with the reviewer that only a fraction of BACE1 is localized within the autophagosomes, whose transport is affected in AP2-KO neurons. We believe that AP2 might also be involved in regulation of BACE1 trafficking in RAB7 positive late endosomal compartments, independently of autophagosomes. Thus, we have reframed the sentences, which now reads as "Together, these data suggest that AP-2 regulates BACE1 protein levels via mediating its intracellular transport in axons".

8. L286 typo BACE1-dependent

Response: We thank the reviewer for this comment. We apologize for the typo and corrected it now.

9. L303 please clarify why TREM2 mutant lines? Do the authors link the mutation to AP2 reduction?

Response: We thank the reviewer for the comments. One of the major goals of our study was to understand the pathophysiological mechanism of AD beyond the autosomal dominant mutations causing AD (i.e. in APP, PSEN1/2). TREM2 is one of the strongest single allele genetic risk factor for sporadic late-onset AD. Thus, we selected TREM2 R47H mutant lines as a model for sporadic late-onset AD. However, at this point, how precisely TREM2 variant could affect AP-2 protein levels is not known. Our prediction is that inflammatory response in microglial cells caused by TREM2 mutation could transcriptionally regulate AP-2 levels in neurons. In fact, recent RNA expression analysis in patients carrying the TREM2 R47H variant identified endocytosis among seven most down-regulated gene sets (Korvatska et al, 2018).

We have now included this hypothesis in the discussion part, page 18: "We show that endocytic adaptor AP-2 is downregulated in iPSC-derived neurons from AD patients with carrying the TREM2^{R47H} risk variant, which is one of the strongest single allele genetic risk factor for sporadic late-onset AD (Korvatska et al, 2015). These results taken together with a recent finding, revealing endocytosis among seven most down-regulated gene sets in the hippocampus of AD TREM2^{R47H} patients (Korvatska et al, 2018), suggest an existing hypothesis that TREM2^{R47H} –induced immune response in microglial cells might transcriptionally regulate AP-2 levels in brains of AD patients".

10. L337 "...and requires its association with LC3-containing autophagosomes,..." the authors should explain which experiment shows the requirement of the association of AP2 with LC3 autophagosomes.

Response: We thank the reviewer for this comment. Current study is a follow-up on the study published in Nat. Commun. 2017 (Kononenko et al., 2017), where we characterized in detail the association of AP-2 with LC3 and introduced LIR mutant of AP-2, which is not able to bind LC3. We apologize for not making this clear in the last version of the manuscript. This is now clarified this on page 11 lines 240-241.

Discussion:
It would be important to discuss what happens to CME in neurons when AP2 is KO.

Response: We thank the Reviewer 1 for this comment. We have now introduced a following sentence in the Discussion: "We find that the endocytic complex AP-2, a clathrin adaptor, previously shown to be not essential for CME in neurons (Kononenko et al, 2014; Soykan et al, 2017), but to function in synaptic vesicles reformation (Kononenko et al, 2014) and autophagosome transport (Kononenko et al, 2017), regulates intracellular trafficking of BACE1".

Figures:
Fig1F and I the channel for rab should be shown alone and merged in order to evaluate the presence of rab4 and rab4SN in BACE1 vesicles.

Response: We have now introduced the overlay images for rab4 and BACE1 colocalizaltion. These images are now shown in Fig. S1K and S1N, due to the lack of space in Fig. 1.

Figure 1H, the authors used eGFP to control Rab11-S25N but an additional control should be rab11 wild type.

Response: We have now introduced eGFP-Rab11 as an additional control, which is presented in Fig.1I. We have moved the graph with eGFP as a control to the Supplementary Fig. S1L.

figure 1 K-M, the authors should also present the total BACE1 quantification, since BACE1 total levels were found increased in vivo (Fig.2A-B).

Response: We would like to note that the total levels of BACE1 in cultured neurons are already shown in Figs. S2F,G. We also now present total BACE1 levels for surface biotinylation experiments (Fig. 1K-M) in Fig. S1P.

Fig2Q-S The data show nicely a decrease of BACE1 in acidic compartments (red) but not the increase in non-acidic compartments (green). Can you explain why not? Or find a better image to represent the data.

Response: We would like to refer to the Fig. S2P, which was the part of the original submission. In this figure, one can appreciate an accumulation of BACE1 in non-acidic compartments in distal neurites (arrows). This phenotype is not obvious due to the absence of distal processes in Fig. 2R. We agree that we did not clearly state this phenomenon in the previous version. On the page 10 (lines 213-214) we now state: "Interestingly, in the absence of AP-2 the majority of BACE1-mKeima organelles with neutral pH was confined to distal processes (Fig. S2P)".

FigS3E the levels of beta-amyloid need to be quantified.

Response: The levels of Amyloidβ1-42 upon BACE1 overexpression are now quantified in Fig. S3F.

Fig3A,B show an important colocalization between exogenous ap2 and bace1, this should be complemented with the colocalization between endogenous proteins.

Response: We have now analyzed Pearson's colocalization coefficient in neurons immunostained with AP-2α and BACE1 antibodies. We found a very strong colocalization between these two proteins (around 0.9). These data are now shown in Fig. S3J-K.

Fig.4D Label panel D with "APP plus CTFs". Or clarify that the antibody used recognizes exclusively APP CTFs but not the full length.

Response: We thank the Reviewer 1 for this comment. We have introduced now the following labelling: APP full length + CTFs.

Fig.4T Although the representative images show reduction in ab42 signal when BACE1 is KD in the graph there is no significant difference in wt neurons KD for BACE1. How do the authors explain this inconsistency?

Response: We understand and appreciate the reviewer's concern. We believe that the absence of the effect of BACE1 shRNA knockdown in WT neurons is explained by very low levels of endogenous Aβ42 in these cells, which contributes to data variability.

We have already discussed this effect on page 14, lines 314-318: "Of note, $Aβ_{1-42}$ peptide levels were not significantly altered by BACE1 KD in WT neurons. This is in agreement with a small effect of BACE1 KO on CTF99 levels (Ou-Yang et al, 2018), likely due to insensitivity of conventional protein detection techniques in analyzing the $Aβ_{1-42}$ picogram range changes in the control condition (Liebsch et al, 2019)".

Fig5C Misses the quantification of number of spines.

Response: As mentioned above, we have now performed a completely new experiment to exclude the role of AP-2 in development and acutely measure the effect of AP-2μ loss on spine morphology in mature brain. To this aim, we delivered either Cre-GFP or control GFP vector under CamKIIalpha promoter to the dentate gyrus of the hippocampus of AP-2flox/flox mice at 9 weeks of age. For N=4 animals we have quantified the spine density and found a significant decrease. These data are now shown in Fig.5C,D.

Fig.5K Please indicate that panel K refers to BACE1 KD

Response: We thank the Reviewer 1 for this comment. We have now added BACE1 next to shRNA.

Referee #2:

This manuscript reports a detailed investigation of the role of AP-2 in the trafficking of BACE1 and its downstream consequences on APP processing and neuronal function. The work is thorough and overall is well performed and controlled.

Response: We thank the Reviewer 1 for this positive comment on our work.

The manuscript contains a considerable amount of data within it, which in places makes it fairly dense and difficult to read. Positive aspects include a series of detailed and complementary assays to conclusively demonstrate the role of AP-2 in intracellular BACE1 trafficking. Weaker points include the lack of demonstration of specificity in terms of BACE1 trafficking. Also in a few places, the experimental n that has been chosen to show significance should be reassessed.

Major points

1) The authors convincingly show that reduction of BACE1 levels can correct the observed dysfunction in the AP-2 knockout mice and neurons. However, an unaddressed question is whether the effects are due to global effects on post-endocytosis trafficking to lysosomes in AP-2 knockout mice, or whether it is specific to disrupted BACE1 traffic. The di-leucine mutant mentioned in the manuscript could address this point, since AP-2 is dispensable for BACE1 endocytosis (Fig 1A,B). Use of this mutant therefore should reveal whether the altered trafficking and subsequent downstream effects were BACE1-specific and or a generalised defect in post-endocytosis trafficking with altered BACE1 trafficking a consequence of that. This mutant could be employed in trafficking assays such as in Figures 1-3, and APP processing experiments in Figure 4 to address this point.

Response: We thank the Reviewer 2 for this positive comment and constructive suggestions. We have gladly followed her/his suggestion and performed a series of experiments, where by using the di-leucine BACE1 mutant we show that:

1. Mutation of BACE1 binding to AP-2 reduces its mobility and axonal transport (Fig. 3L-N)
2. Delayed delivery to lysosomes and recycling defect of BACE1 in AP-2 KO neurons can be recapitulated by using BACE1 LL/AA mutant (Fig. S3R-U)
3. APP processing towards Aβ42 is increased in neurons overexpressing BACE1 LL/AA mutant (Fig. 4U,V).

Taken together, our new data indicate that BACE1 trafficking defects and the downstream effect on amyloidogenesis in AP-2 KO neurons are specific to BACE1 and require the interaction with the AP-2.

2) Related to the point above, are the total protein levels, internalisation kinetics or surface fraction of other cargo molecules unaltered by the absence of AP-2?

Response: We would like to note that in our previous study (Kononenko et al., 2017(Fig. S3c,d)) we have already analyzed the total levels of several endocytic proteins (CHC, ITSN1, Amph1, Dyn1, Stn2), as well as the levels of CME cargo, such as Syt1 and Syph in AP-2 KO neurons. For none of mentioned proteins we detected significant changes in total protein levels. We apologize for not making this clear in the manuscript. We have now inserted this information in the manuscript page 8 (lines 179-181).

Additionally, we have now analyzed the surface fraction of GABARB3 receptor, a known cargo of CME in neurons. We found that the surface fraction of GABARB3 receptors is significantly increased in neurons lacking AP-2 (Fig. S1N,O). Taken together, our data indicate a cargo-specific requirement for AP-2 in endocytosis in neurons.

3) Again related to specificity, are other BACE1 substrates similarly affected in the absence of AP-2, or is this defect specific to APP cleavage?

Response: We have now analyzed the processing of another BACE1 substrate, L1. Strikingly, we found that L1 processing is increased in AP-2 KO brains (Fig. S4G,H). This data are in line with our hypothesis that BACE1 is overactive in AP-2 KO neurons, due its stalled delivery to lysosomes for degradation.

4) Many of the experiments presented in this manuscript are display relatively modest effects, which are significant due to the high power provided by using either individual neurons or axonal lengths as experimental n. Individual neurons could be viewed as being the major source of variation in this type of analysis and therefore acceptable as experimental n. However, I do not really think the same can be said for axonal lengths (and definitely not puncta, such as used in Figure 4B). The authors should try to present their data using at least the neuron as the basic unit for statistical analysis wherever possible.

Response: We thank the Reviewer 2 for this constructive suggestion. We agree with the reviewer and have now re-plotted and statistically re-probed all data sets. Statistical analysis for all in-vitro data was now performed with individual neurons from N independent experiments.

Minor points
1) Fig 1A suggests that BACE1 is internalised in an AP-2 independent manner. Can the authors speculate as to how BACE1 internalisation (and downstream effects) will be altered during neuronal

activity? For example, would any effect be exacerbated, since there will be greater BACE1 turnover and hence more Abeta production (leading to a positive feedback loop of increased hyperexcitability and further BACE1 internalisation)?

Response: We thank the Reviewer 2 for this excellent suggestion. Indeed the lack of AP-2 would not prevent the internalization of BACE1 during neuronal activity. In contrary, it will facilitate Abeta generation due defective BACE1 degradation, causing deleterious effects on neuronal function, including the change in excitability.

We have now added this hypothesis in the discussion, page 20:
"In fact, recent data highlight BACE1 enrichment in synaptic vesicles (Lundgren et al, 2015), a presynaptic organelle, which is regenerated upon neuronal activity in AP-2 dependent manner (Kononenko et al, 2014). Thus, our data propose a model where in neurons AP-2 functions at presynaptic membrane compartments to sort BACE1 to late endosomes and autophagosomes, thus regulating its degradation during neuronal activity. Lack of AP-2 could trigger a positive feedback loop between BACE1 internalization and Aβ production in highly active neurons, which in turn might lead to hyperexcitability due to intrinsic Aβ effect on expression of voltage-gated sodium channels (Wang et al, 2016).

2) Figure 1P is not referenced in the text.

Response: We thank the Reviewer 2 for this comment. The reference for Fig.1P is now introduced on page 8

3) How specific is the decrease in AP-2 in human patient iPSCs? Are other adaptor protein complexes decreased too?

Response: We have now analyzed the levels of gamma Adaptin (AP-1γ1) in iPSCs lysates. We have not detected any changes in the levels of this adaptor protein complex (Fig. S5A).

References:

Garafalo SD, Luth ES, Moss BJ, Monteiro MI, Malkin E, Juo P (2015) The AP2 clathrin adaptor protein complex regulates the abundance of GLR-1 glutamate receptors in the ventral nerve cord of Caenorhabditis elegans. Molecular Biology of the Cell 26: 1887-1900

Kononenko NL, Claßen GA, Kuijpers M, Puchkov D, Maritzen T, Tempes A, Malik AR, Skalecka A, Bera S, Jaworski J, Haucke V (2017) Retrograde transport of TrkB-containing autophagosomes via the adaptor AP-2 mediates neuronal complexity and prevents neurodegeneration. Nature Communications 8: 1-16

Kononenko NL, Puchkov D, Classen GA, Walter AM, Pechstein A, Sawade L, Kaempf N, Trimbuch T, Lorenz D, Rosenmund C, Maritzen T, Haucke V (2014) Clathrin/AP-2 Mediate Synaptic Vesicle Reformation from Endosome-like Vacuoles but Are Not Essential for Membrane Retrieval at Central Synapses. Neuron 82: 981-988

Korvatska O, Kiianitsa K, Ratushny A, Matsushita M, Chien W-M, Dorschner MO, Keene CD, Bammler TK, Bird TD, Raskind WH (2018) TREM2 R47H exacerbates immune response in Alzheimer's disease brain. bioRxiv: 499319

Korvatska O, Leverenz JB, Jayadev S, McMillan P, Kurtz I, Guo X, Rumbaugh M, Matsushita M, Girirajan S, Dorschner MO, Kiianitsa K, Yu C-E, Brkanac Z, Garden GA, Raskind WH, Bird TD (2015) R47H Variant of TREM2 Associated With Alzheimer Disease in a Large Late-Onset Family: Clinical, Genetic, and Neuropathological Study. JAMA neurology 72: 920-927

Liebsch F, Kulic L, Teunissen C, Shobo A, Ulku I, Engelschalt V, Hancock MA, van der Flier WM, Kunach P, Rosa-Neto P, Scheltens P, Poirier J, Saftig P, Bateman RJ, Breitner J, Hock C, Multhaup

G (2019) Aβ34 is a BACE1-derived degradation intermediate associated with amyloid clearance and Alzheimer's disease progression. Nature Communications 10: 2240

Lundgren JL, Ahmed S, Schedin-Weiss S, Gouras GK, Winblad B, Tjernberg LO, Frykman S (2015) ADAM10 and BACE1 are localized to synaptic vesicles. Journal of Neurochemistry 135: 606-615

Ou-Yang M-H, Kurz JE, Nomura T, Popovic J, Rajapaksha TW, Dong H, Contractor A, Chetkovich DM, Tourtellotte WG, Vassar R (2018) Axonal organization defects in the hippocampus of adult conditional BACE1 knockout mice. Science Translational Medicine 10: eaao5620

Soykan T, Kaempf N, Sakaba T, Vollweiter D, Goerdeler F, Puchkov D, Kononenko NL, Haucke V (2017) Synaptic Vesicle Endocytosis Occurs on Multiple Timescales and Is Mediated by Formin-Dependent Actin Assembly. Neuron 93: 854-866.e854

Wang X, Zhang X-G, Zhou T-T, Li N, Jang C-Y, Xiao Z-C, Ma Q-H, Li S (2016) Elevated Neuronal Excitability Due to Modulation of the Voltage-Gated Sodium Channel Nav1.6 by Aβ1−42. Frontiers in Neuroscience 10

2nd Editorial Decision 5 March 2020

Thank you for the submission of your revised manuscript. We have now received the enclosed comments from the referees who were asked to assess it. I am happy to say that both referees support the publication of your revised study. Please address all remaining referee concerns in the final manuscript file.

A few other changes will also be required:

The image in Fig 2A is over-contrasted, please replace it with a better image.

The Fig EV5L second panel from left on the bottom has a random letter "K" on top of the image, please correct.

Figures 1B, 3C and F top panels, EV3M and P top panels, and EV4P are missing scale bars, please add.

Please add up to 5 keywords for your manuscript.

In the author contributions SN should be SM.

The reference format is incorrect. Please correct to the numbered EMBO reports style that can be found in EndNnote.

The manuscript has callouts to Table S2 + S3. These tables need to be uploaded as individual excel or word files and need to be called Table EV1 and Table EV2.
If there are more than 2 tables please label accordingly.

The movie files need to be ZIPed with their legends and uploaded as one file per movie, and the legends need to be removed from the article file.

Please delete the Data Availability Section from the methods if you have not deposited any novel data in public databases.

I attach to this email a related manuscript file with comments by our data editors. Please address all comments in the final manuscript file.

I would like to suggest a change to the manuscript title. Please let me know if you agree with the following:

"AP-2 prevents amyloidogenesis by promoting BACE1 trafficking in neurons"

EMBO press papers are accompanied online by A) a short (1-2 sentences) summary of the findings and their significance, B) 2-3 bullet points highlighting key results and C) a synopsis image that is 550x200-400 pixels large (the height is variable). You can either show a model or key data in the synopsis image. Please note that text needs to be readable at the final size. Please send us this information along with the revised manuscript.

I look forward to seeing a final version of your manuscript as soon as possible. Please let me know if you have any comments or questions.

REFEREE REPORTS

Referee #1:

I want to thank the authors for addressing most of the points I raised. The paper is now excellent and almost ready for publication. I only have two critical concerns and one minor one.

1. In the abstract, it reads "Here we demonstrate that AP-2 affects APP processing and Aβ production in neurons via the regulation of BACE1 intracellular trafficking"

However, the authors need to consider that APP endocytosis is also required for APP processing and Abeta generation. This abstract sentence implies that AP-2 does not contribute to APP endocytosis. But, evidence indicates that APP endocytosis is clathrin- and AP2-dependent. Thus it is recommended that it is experimentally determined the impact of AP2 KO on APP endocytosis. This experiment would strengthen your findings since your data for BACE1 endocytosis does completely replicate other group's results. Otherwise, it would be better to rephrase it to something along the lines "AP-2 regulation of BACE1 intracellular trafficking contributes to APP processing and Abeta production..."

2. Regarding the use of BACE1 di-leucine mutant to block the interaction with AP-2. I appreciate the effort to try to support your AP2 KO data with a mutant of BACE1 that does not bind AP2. However, previous work showed strong evidence for this mutation to block BACE1 endocytosis:
- "...the disruption of the di-leucine motif (BACE1LLAA) greatly impairs BACE1 endocytosis..." (Kang EL Et al 2012; doi: 10.1074/jbc.M112.407072.)
- "Here we show that the DISLL sequence is embedded within a longer [DE]XXXL[LI]-motif sequence, DDISLL, which mediates internalization from the plasma membrane by interaction with the clathrin-associated, heterotetrameric adaptor protein 2 (AP-2) complex. Mutation of this signal or knockdown of either AP-2 or clathrin decreases endosomal localization and increases plasma membrane localization of BACE1. "(Prabhu, Y et al. 2012; https://doi.org/10.1091/mbc.e11-11-0944)
Or to block BACE1 delivery to early endosomes:
- "... dileucine motif is required for sorting of BACE1 from a transient preendosomal compartment to early endosomes" (Sannerud et al 2011; https://doi.org/10.1073/pnas.1100745108)

Thus, it is recommended that BACE1 dileucine mutant endocytosis should be done in neurons. The result will strengthen the current interpretation of the results.

3. The minor concern refers to the color used for most figures. It is recommended to use a grayscale whenever there is only one channel. The use of red is highly discouraged. There is a large percentage of color-blind people and because it is difficult to see with the reduced contrast against the black background. The exceptions are when merged channels are necessary for identification of colocalization or juxtaposition; in that case, magenta is better than red.

Referee #2:

The authors have addressed my concerns regarding the original submission and I am happy to recommend the revised version for publication.

2nd Revision - authors' response 11 March 2020

Reviewer #1 (Remarks to the Author):

I want to thank the authors for addressing most of the points I raised. The paper is now excellent and almost ready for publication.

Response: We thank the Reviewer 1 for this positive comment on our work.

I only have two critical concerns and one minor one.

1. In the abstract, it reads "Here we demonstrate that AP-2 affects APP processing and Aβ production in neurons via the regulation of BACE1 intracellular trafficking"

However, the authors need to consider that APP endocytosis is also required for APP processing and Abeta generation. This abstract sentence implies that AP-2 does not contribute to APP endocytosis. But, evidence indicates that APP endocytosis is clathrin- and AP2-dependent. Thus it is recommended that it is experimentally determined the impact of AP2 KO on APP endocytosis. This experiment would strengthen your findings since your data for BACE1 endocytosis does completely replicate other group's results. Otherwise, it would be better to rephrase it to something along the lines "AP-2 regulation of BACE1 intracellular trafficking contributes to APP processing and Abeta production..."

Response: We thank the Reviewer 1 for this comment. We completely agree with the reviewer. In fact, the endocytosis of APP is slightly decreased in AP-2μ KO neurons. These data are already shown in Fig. EV4I,J and discussed on the page 13, lines 300-301. To clarify this phenomenon in the abstract, we have now modified the text accordingly:

"Here, we show that in neurons APP processing and Aβ production are controlled by the protein complex-2 (AP-2), an endocytic adaptor known to be required for APP endocytosis. Now we find that AP-2 prevents amyloidogenesis by additionally functioning downstream of BACE1 endocytosis, regulating BACE1 endosomal trafficking and its delivery to lysosomes."

2. Regarding the use of BACE1 di-leucine mutant to block the interaction with AP-2. I appreciate the effort to try to support your AP2 KO data with a mutant of BACE1 that does not bind AP2. However, previous work showed strong evidence for this mutation to block BACE1 endocytosis:
- "...the disruption of the di-leucine motif (BACE1LLAA) greatly impairs BACE1 endocytosis..." (Kang EL Et al 2012; doi: 10.1074/jbc.M112.407072.)
- "Here we show that the DISLL sequence is embedded within a longer [DE]XXXL[LI]-motif sequence, DDISLL, which mediates internalization from the plasma membrane by interaction with the clathrin-associated, heterotetrameric adaptor protein 2 (AP-2) complex. Mutation of this signal or knockdown of either AP-2 or clathrin decreases endosomal localization and increases plasma membrane localization of BACE1. "(Prabhu, Y et al. 2012; https://doi.org/10.1091/mbc.e11-11-0944)
Or to block BACE1 delivery to early endosomes:
- "... dileucine motif is required for sorting of BACE1 from a transient preendosomal compartment to early endosomes" (Sannerud et al 2011; https://doi.org/10.1073/pnas.1100745108)

Thus, it is recommended that BACE1 dileucine mutant endocytosis should be done in neurons. The result will strengthen the current interpretation of the results.

Response: We understand and appreciate the Reviewer 1 concern on endocytosis of BACE1 di-leucine mutant. We would like to note that the experiment presented in Fig. EV3 R,S already addresses the BACE1 LL/AA endocytosis in neurons. In this assay, the neurons overexpressing the

HA-BACE1Ll/AA-GFP were fed with the HA antibody and the amount of the HA-bound BACE1, which is taken up and recycled back to the plasma membrane is measured after surface stripping. Our results indicate that the di-leucine mutant is endocytosed, but then it is more rapidly recycled. In fact, these data are in strong agreement with Sannerud et al., 2011 cited by the Reviewer 1 above, which shows that "BACE1-AA is still endocytosed and remains responsive to EGF (Fig. 6 F). The lower extent of internalized BACE1-AA could be explained by the high recycling kinetics of this mutant to the surface as demonstrated by Huse et al. (35)".

Additionally, impaired delivery of BACE1 LL/AA to lysosomes and its accumulation in early endosomes is also in agreement with the Kang et al., 2012, page 42872 "Instead, BACE1KR and BACE1LLAA accumulate in early endosomes (EEA1-positive compartments) (Fig. 4). These data indicate that both ubiquitination at Lys-501 and the di-leucine motif regulate the delivery of BACE1 to the lysosomes".

It is possible, if not very likely that decreased endocytosis kinetics of BACE1 LL/AA reported previously and delayed internalization of BACE1 in AP-2 KO neurons reported in this work (Fig. 1A,B) are due to increased recycling kinetics of BACE1 without AP-2. Furthermore, the nature of pre-endosomal compartment, where BACE1 LL/AA is sequestered might be different in neurons versus HeLA cells (or other cell lines). Irrespective of this, our data indicate that post-endocytosis BACE1 trafficking is regulated by the AP-2 and that this function of AP-2 is crucial for amyloidogenesis in neurons.

To make this point clear in the manuscript, we have now modified the text accordingly (page 12):

"Furthermore, this defect was accompanied by increased recycling of endocytosed BACE1 to the plasma membrane, in agreement with (Sannerud et al., 2011) and (Huse et al., 2000) and its impaired delivery to lysosomes, similar to the phenotype described previously in neuroglioma cells (Kang et al, 2012) (Figure EV3R-U). Together, these data suggest that AP-2 regulates BACE1 protein levels in primary neurons via functioning downstream of BACE1 endocytosis, regulating BACE1 endosomal trafficking and its delivery to lysosomes".

3. The minor concern refers to the color used for most figures. It is recommended to use a grayscale whenever there is only one channel. The use of red is highly discouraged. There is a large percentage of color-blind people and because it is difficult to see with the reduced contrast against the black background. The exceptions are when merged channels are necessary for identification of colocalization or juxtaposition; in that case, magenta is better than red.

Response: We thank the Reviewer 1 for this comment. We have now modified the color-code for all figures, which are now represented as green/magenta.

Referee #2:

The authors have addressed my concerns regarding the original submission and I am happy to recommend the revised version for publication.

Response: We thank the Reviewer 2 for this positive comment on our work.

Accepted 13 March 2020

I am very pleased to accept your manuscript for publication in the next available issue of EMBO reports. Thank you for your contribution to our journal.

EMBO PRESS

YOU MUST COMPLETE ALL CELLS WITH A PINK BACKGROUND ↓

PLEASE NOTE THAT THIS CHECKLIST WILL BE PUBLISHED ALONGSIDE YOUR PAPER

Corresponding Author Name: Natalia Kononenko

Journal Submitted to: EMBO Reports

Manuscript Number: EMBOR-2019-47954

USEFUL LINKS FOR COMPLETING THIS FORM

http://www.antibodypedia.com	Antibodypedia
http://1degreebio.org	1DegreeBio
http://www.equator-network.org/reporting-guidelines/improving-bioscience-research-repor	ARRIVE Guidelines
http://grants.nih.gov/grants/olaw/olaw.htm	NIH Guidelines in animal use
http://www.mrc.ac.uk/Ourresearch/Ethicsresearchguidance/Useofanimals/index.htm	MRC Guidelines on animal use
http://ClinicalTrials.gov	Clinical Trial registration
http://www.consort-statement.org	CONSORT Flow Diagram
http://www.consort-statement.org/checklists/view/32-consort/66-title	CONSORT Check List
http://www.equator-network.org/reporting-guidelines/reporting-recommendations-for-tumo	REMARK Reporting Guidelines (marker prognostic studies)
http://datadryad.org	Dryad
http://figshare.com	Figshare
http://www.ncbi.nlm.nih.gov/gap	dbGAP
http://www.ebi.ac.uk/ega	EGA
http://biomodels.net/	Biomodels Database
http://biomodels.net/miriam/	MIRIAM Guidelines
http://jjj.biochem.sun.ac.za	JWS Online
http://oba.od.nih.gov/biosecurity/biosecurity_documents.html	Biosecurity Documents from NIH
http://www.selectagents.gov/	List of Select Agents

Reporting Checklist For Life Sciences Articles (Rev. June 2017)

This checklist is used to ensure good reporting standards and to improve the reproducibility of published results. These guidelines are consistent with the Principles and Guidelines for Reporting Preclinical Research issued by the NIH in 2014. Please follow the journal's authorship guidelines in preparing your manuscript.

A- Figures

1. Data

The data shown in figures should satisfy the following conditions:

- ➔ the data were obtained and processed according to the field's best practice and are presented to reflect the results of the experiments in an accurate and unbiased manner.
- ➔ figure panels include only data points, measurements or observations that can be compared to each other in a scientifically meaningful way.
- ➔ graphs include clearly labeled error bars for independent experiments and sample sizes. Unless justified, error bars should not be shown for technical replicates.
- ➔ if n< 5, the individual data points from each experiment should be plotted and any statistical test employed should be justified
- ➔ Source Data should be included to report the data underlying graphs. Please follow the guidelines set out in the author ship guidelines on Data Presentation.

2. Captions

Each figure caption should contain the following information, for each panel where they are relevant:

- ➔ a specification of the experimental system investigated (eg cell line, species name).
- ➔ the assay(s) and method(s) used to carry out the reported observations and measurements
- ➔ an explicit mention of the biological and chemical entity(ies) that are being measured.
- ➔ an explicit mention of the biological and chemical entity(ies) that are altered/varied/perturbed in a controlled manner.
- ➔ the exact sample size (n) for each experimental group/condition, given as a number, not a range;
- ➔ a description of the sample collection allowing the reader to understand whether the samples represent technical or biological replicates (including how many animals, litters, cultures, etc.).
- ➔ a statement of how many times the experiment shown was independently replicated in the laboratory.
- ➔ definitions of statistical methods and measures:
  - common tests, such as t-test (please specify whether paired vs. unpaired), simple $\chi 2$ tests, Wilcoxon and Mann-Whitney tests, can be unambiguously identified by name only, but more complex techniques should be described in the methods section;
  - are tests one-sided or two-sided?
  - are there adjustments for multiple comparisons?
  - exact statistical test results, e.g., P values = x but not P values < x;
  - definition of 'center values' as median or average;
  - definition of error bars as s.d. or s.e.m.

Any descriptions too long for the figure legend should be included in the methods section and/or with the source data.

In the pink boxes below, please ensure that the answers to the following questions are reported in the manuscript itself. Every question should be answered. If the question is not relevant to your research, please write NA (non applicable). We encourage you to include a specific subsection in the methods section for statistics, reagents, animal models and human subjects.

B- Statistics and general methods

Please fill out these boxes ↓ (Do not worry if you cannot see all your text once you press return)

1.a. How was the sample size chosen to ensure adequate power to detect a pre-specified effect size?	Sample sizes were not chosen based on pre-specified effect size. Instead, multiple independent experiments were carried out using several sample replicates as detailed in the figure legends. For all experiments there was enough statistical power to detect the corresponding effect size.
1.b. For animal studies, include a statement about sample size estimate even if no statistical methods were used.	Minimum N=3 animals were used as independent experiments
2. Describe inclusion/exclusion criteria if samples or animals were excluded from the analysis. Were the criteria pre-established?	Data was only excluded when the quality of the sample was not optimal. Predefined quality criteria were: in living samples- vacuolization or other signs of cellular degeneration, normal cell morphology and protein transport (which is usually hampered in unhealthy cells) and image streams that are in focus; proper sample preparation and mounting in fixed samples.
3. Were any steps taken to minimize the effects of subjective bias when allocating animals/samples to treatment (e.g. randomization procedure)? If yes, please describe.	Data were acquired and analyzed based on the standards in the field, however, no method of randomization was used to determine how sample were allocated to experimental groups and processed.
For animal studies, include a statement about randomization even if no randomization was used.	No randomization was used, since mice were genotypes prior to experiments and the KO mice wre visually different from the WT
4.a. Were any steps taken to minimize the effects of subjective bias during group allocation or/and when assessing results (e.g. blinding of the investigator)? If yes please describe.	N/A
4.b. For animal studies, include a statement about blinding even if no blinding was done	No bldning was used, since mice were genotypes prior to experiments and the KO mice wre visually different from the WT
5. For every figure, are statistical tests justified as appropriate?	Yes
Do the data meet the assumptions of the tests (e.g., normal distribution)? Describe any methods used to assess it.	Normality test using GraphPad. Non-normally distributed data (as in Figure EV4N,O) were transformed using square root transformation and analyzed using paired two-tailed t test.
Is there an estimate of variation within each group of data?	N/A
Is the variance similar between the groups that are being statistically compared?	Each experiment was replicated a minimum of three times and data was reliably reproduced with each replication attempt.

C- Reagents

6. To show that antibodies were profiled for use in the system under study (assay and species), provide a citation, catalog number and/or clone number, supplementary information or reference to an antibody validation profile. e.g., Antibodypedia (see link list at top right), 1DegreeBio (see link list at top right).	Mouse monoclonal (AP6) Anti-AP2-alpha - 1:300 - Abcam Ab2730 (Loss of signal in the KO mouse (this paper))<br>Mouse monoclonal (8) Anti-AP2-alpha 1:1000 - - BD Transduction Cat# 610501 (Loss of signal in the KO mouse (this paper))<br>Mouse monoclonal (31) Anti AP2-mu 1:1000 - - BD Transduction Cat# 611350 (Loss of signal in the KO mouse (this paper))<br>Mouse monoclonal (88) Anti AP1-gamma 1:1000 - - BD Transduction Cat#610385 (Robinson MS, JCB, 1990)<br>Mouse monoclonal (Ab40.1) Anti-Aβ 1-40 - 1:100 - QED Bioscience Inc Cat# 57002 (Levites Y et al., JCI, 2006)<br>Mouse monoclonal (12F4) Anti-Aβ 1-42 - 1:100 1:100 EMD, Millipore Cat# 05-831-I (Parvathy et al., JAMA Neurology, 2001)<br>Rabbit polyclonal Anti-APP 1:1000 1:300 1:250 Sigma-Aldrich Cat# A8717 (Tanzi, R.E., et al.,<br>Nature, 1988)<br>Rabbit polyclonal Anti-BACE1 1:1000 1:300 1:500 Thermo Fischer Cat# PA1-757 (Ubelmann et al., EMBO Rep 2016)<br>Mouse monoclonal (SAP7F407) Anti-Bassoon - 1:300 1:800 Abcam Cat# ab82958 (Neef et al., Nat Commun, 2018)<br>Rabbit polyclonal Anti-Bassoon - 1:300 - Synaptic systems Cat# 141002 (Blanco-Suarez et al., Neuron, 2018)<br>Rabbit polyclonal Anti-Caspase-3 cleaved - 1:200 Cell signaling Cat# 9661 (Wu, Young, et al., Nat Commun, 2019)<br>Goat polyclonal Anti-Cathepsin D - 1:300 - R & D Systems Cat# AF1029 (Atiskova et al., Sci Rep, 2019)<br>Mouse monoclonal (71.1) Anti-GAPDH 1:3000 - - Sigma-Aldrich Cat# G8795 (Bieri, Brahic, et al., Acta Neuropathologica, 2019)<br>Rabbit polyclonal Anti-GABARB3 N-terminal - 1:200 - Abcam Cat#66430 (Desrues et al., Plos One, 2012)<br>Mouse monoclonal Anti-GABARB3 (N87/25) C-terminal - 1:200 - Abcam Cat#ab98968 (Urraca et al., Mol Autism, 2018)<br>Chicken polyclonal anti-GFP - 1:5000 - Abcam Cat# ab13970 (Al-Moyed et al., EMBO Mol Med, 2019)<br>Mouse monoclonal (16B12) Anti-HA - 1:300 - Biolegend Cat# MMS-101P (Bennett BD, et al. J Biol Chem. 2000)<br>Rabbit polyclonal Anti-L1 1:1000 - - Aviva Systems Biology Cat#OAAB11621 (Verification in mouse cerebellum is presented on manufacture website)<br>Mouse monoclonal (4E12) Anti-LC3 - 1:300 - MBL Cat# M152-3B (Saiki et al., Autophagy, 2011)<br>Rabbit polyclonal Anti-LAMP2a - - 1:500 Abcam Cat# 18528 (Perera et al., Autophagy, 2018)<br>Rabbit monoclonal (D38F9) Anti-Nicastrin 1:1000 - - Cell Signaling Cat# 5665 (Chai, Jovasevic et al., Nat Commun, 2017)<br>Rabbit polyclonal Anti-PSD95 - 1:300 1:200 Protein-Tech Europe Cat# 20665-1-AP (Verification in mouse cerebellum is presented on manufacture website)
	Mouse monoclonal (108E10) Anti-PSD95 - 1:300 - Synaptic systems Cat# 124011 (Emperador-Melero et al., Nat. Commun, 2018)<br>Goat polyclonal Anti-RAB7A - - 1:250 Origene Cat#AB0033-200 (Verification is presented on manufacture website)<br>Mouse monoclonal (G10) Anti-Reelin - - 1:500 Abcam Cat#ab78540 (Murthy et al., Nat Commun, 2014)<br>Mouse monoclonal (621.3) Anti-RAB5 - 1:300 - SySy Cat#108 011 (Ackermann et al., eLife, 2019)<br>Mouse monoclonal (1C51) Anti-mCherry - 1:2000 - Novus Biologicals Cat# NBP1-96752SS (Ruas et al., EMBO J, 2015)<br>Rabbit polyclonal Anti-mCherry - 1:1000 Abcam Cat#ab167453 (Tapia et al., Nat Commun, 2019)<br>Mouse monoclonal Anti-FLAG 1:1000 - - Sigma-Aldrich Cat# F3165 (Pajak et al., PLOS Genetics, 2019)<br>Goat anti-Mouse IgG (H+L) peroxidase-conjugated 1:5000 - - Jackson ImmunoResearch Cat# 115-035-003 (Djeungoue-Petga et al., Cell Death & Disease, 2019)<br>Goat anti-Rabbit IgG (H+L) peroxidase-conjugated 1:5000 - - Jackson ImmunoResearch Cat# 111-035-003 (Eisenberg-Lerner et al., Nat Commun, 2020)<br>Alexa Fluor 488 Goat anti-Mouse IgG - 1:500 1:500 Thermo Fisher Cat# A-11029 (Feng et al., Autophagy, 2017)<br>Alexa Fluor 488 Goat anti-Rabbit IgG - 1:500 1:500 Thermo Fisher Cat# A-11034 (Lopez et al., Nat Medicine, 2014)<br>Alexa Fluor 488 Goat anti-Chicken IgG - 1:500 - Thermo Fisher Cat# A-11039 (Quadrato et al., Nature, 2017)<br>Alexa Fluor 568 Goat anti-Mouse IgG - 1:500 1:500 Thermo Fisher Cat# A-11031 (van de Ven et al., Nat Commun, 2016)<br>Alexa Fluor 568 Goat anti-Rabbit IgG - 1:500 1:500 Thermo Fisher Cat# A-11036 (Feng et al., Autophagy, 2017)<br>Alexa Fluor 647 Goat anti-Mouse IgG - 1:500 - Thermo Fisher Cat# A-21236 (Nassal et al., eLife, 2017)<br>Alexa Fluor 647 Goat anti-Rabbit IgG - 1:500 - Thermo Fisher Cat# A-21245 (Inoue et al., 2017, Nat Commun)<br>Normal Rabbit IgG - Cell Signalling Cat# 2729 (Smith et al., Nat Commun, 2019)<br>Normal Mouse IgG - Merck Millipore Cat# 12-371 (Verification is provided on manufacture website)
7. Identify the source of cell lines and report if they were recently authenticated (e.g., by STR profiling) and tested for mycoplasma contamination.	Human embryonic kidney (HEK) 293T were kindly provided by Prof Dr Thomas Langer. Cell lines were not tested for mycoplasma contamination

* for all hyperlinks, please see the table at the top right of the document

D- Animal Models

8. Report species, strain, gender, age of animals and genetic modification status where applicable. Please detail housing and husbandry conditions and the source of animals.	C57/BL/6 mice were housed in polycarbonate cages at standard 12/12 day-night cycles and water and food were provided ad libitum. Conditional tamoxifen-inducible (Ap-2µlox/lox x inducible CAG-Cre) and neuron-confined (Ap-2µlox/lox x Tubulinα1α-Cre) AP-2µ KO mice, as well as conditional tamoxifen-inducible ATG5 KO (Atg5flox/flox: B6.Cg-Tg(CAG-Cre/Esr1*)5Amc/J) were described previously (Kononenko et al, 2017). Neuron-confined AP-2µ KO mice were crossbred with BACE1 KO mice (Bace1tm1Pcw, Jackson Laboratory, Stock #: 004714) to obtain the AP-2µ KO: BACE1 HET mice.
9. For experiments involving live vertebrates, include a statement of compliance with ethical regulations and identify the committee(s) approving the experiments.	All animal experiments were approved by the ethics committee of LANUV Cologne and were conducted according to the committee's guidelines.
10. We recommend consulting the ARRIVE guidelines (see link list at top right) (PLoS Biol. 8(6), e1000412, 2010) to ensure that other relevant aspects of animal studies are adequately reported. See author guidelines, under 'Reporting Guidelines'. See also: NIH (see link list at top right) and MRC (see link list at top right) recommendations. Please confirm compliance.	N/A

E- Human Subjects

11. Identify the committee(s) approving the study protocol.	We used previously published iPSC lines derived from AD patients, as well as control individuals without dementia for the current study (Martins et al, 2018; Schröter et al, 2016a; Schröter et al, 2016c; Schröter et al, 2016e).
12. Include a statement confirming that informed consent was obtained from all subjects and that the experiments conformed to the principles set out in the WMA Declaration of Helsinki and the Department of Health and Human Services Belmont Report.	N/A
13. For publication of patient photos, include a statement confirming that consent to publish was obtained.	N/A
14. Report any restrictions on the availability (and/or on the use) of human data or samples.	N/A
15. Report the clinical trial registration number (at ClinicalTrials.gov or equivalent), where applicable.	N/A
16. For phase II and III randomized controlled trials, please refer to the CONSORT flow diagram (see link list at top right) and submit the CONSORT checklist (see link list at top right) with your submission. See author guidelines, under 'Reporting Guidelines'. Please confirm you have submitted this list.	N/A
17. For tumor marker prognostic studies, we recommend that you follow the REMARK reporting guidelines (see link list at top right). See author guidelines, under 'Reporting Guidelines'. Please confirm you have followed these guidelines.	N/A

F- Data Accessibility

18: Provide a "Data Availability" section at the end of the Materials & Methods, listing the accession codes for data generated in this study and deposited in a public database (e.g. RNA-Seq data: Gene Expression Omnibus GSE39462, Proteomics data: PRIDE PXD000208 etc.) Please refer to our author guidelines for 'Data Deposition'.<br><br>Data deposition in a public repository is mandatory for:<br>a. Protein, DNA and RNA sequences<br>b. Macromolecular structures<br>c. Crystallographic data for small molecules<br>d. Functional genomics data<br>e. Proteomics and molecular interactions	N/A
19. Deposition is strongly recommended for any datasets that are central and integral to the study; please consider the journal's data policy. If no structured public repository exists for a given data type, we encourage the provision of datasets in the manuscript as a Supplementary Document (see author guidelines under 'Expanded View' or in unstructured repositories such as Dryad (see link list at top right) or Figshare (see link list at top right).	N/A
20. Access to human clinical and genomic datasets should be provided with as few restrictions as possible while respecting ethical obligations to the patients and relevant medical and legal issues. If practically possible and compatible with the individual consent agreement used in the study, such data should be deposited in one of the major public access-controlled repositories such as dbGAP (see link list at top right) or EGA (see link list at top right).	N/A
21. Computational models that are central and integral to a study should be shared without restrictions and provided in a machine-readable form. The relevant accession numbers or links should be provided. When possible, standardized format (SBML, CellML) should be used instead of scripts (e.g. MATLAB). Authors are strongly encouraged to follow the MIRIAM guidelines (see link list at top right) and deposit their model in a public database such as Biomodels (see link list at top right) or JWS Online (see link list at top right). If computer source code is provided with the paper, it should be deposited in a public repository or included in supplementary information.	N/A

G- Dual use research of concern

22. Could your study fall under dual use research restrictions? Please check biosecurity documents (see link list at top right) and list of select agents and toxins (APHIS/CDC) (see link list at top right). According to our biosecurity guidelines, provide a statement only if it could.	No
